# Eye-tracking control of an adjustable electric bed: construction and validation by immobile patients with multiple sclerosis

**DOI:** 10.1186/s12984-023-01193-w

**Published:** 2023-06-09

**Authors:** Martin Kopecek, Jan Kremlacek

**Affiliations:** Department of Medical Biophysics, Faculty of Medicine in Hradec Kralove, Charles University, Simkova 870, Hradec Kralove, Czech Republic

**Keywords:** Fully adjustable electric bed, Multiple sclerosis, Eye-tracking, BCI, Assistive technologies, EDSS

## Abstract

**Background:**

In severe conditions of limited motor abilities, frequent position changes for work or passive and active rest are essential bedside activities to prevent further health complications. We aimed to develop a system using eye movements for bed positioning and to verify its functionality in a control group and a group of patients with significant motor limitation caused by multiple sclerosis.

**Methods:**

The eye-tracking system utilized an innovative digital-to-analog converter module to control the positioning bed via a novel graphical user interface. We verified the ergonomics and usability of the system by performing a fixed sequence of positioning tasks, in which the leg and head support was repeatedly raised and then lowered. Fifteen women and eleven men aged 42.7 ± 15.9 years in the control group and nine women and eight men aged 60.3 ± 9.14 years in the patient group participated in the experiment. The degree of disability, according to the Expanded Disability Status Scale (EDSS), ranged from 7 to 9.5 points in the patients. We assessed the speed and efficiency of the bed control and the improvement during testing. In a questionnaire, we evaluated satisfaction with the system.

**Results:**

The control group mastered the task in 40.2 s (median) with an interquartile interval from 34.5 to 45.5 s, and patients mastered the task in in 56.5 (median) with an interquartile interval from 46.5 to 64.9 s. The efficiency of solving the task (100% corresponds to an optimal performance) was 86.3 (81.6; 91.0) % for the control group and 72.1 (63.0; 75.2) % for the patient group. Throughout testing, the patients learned to communicate with the system, and their efficiency and task time improved. A correlation analysis showed a negative relationship (rho = − 0.587) between efficiency improvement and the degree of impairment (EDSS). In the control group, the learning was not significant. On the questionnaire survey, sixteen patients reported gaining confidence in bed control. Seven patients preferred the offered form of bed control, and in six cases, they would choose another form of interface.

**Conclusions:**

The proposed system and communication through eye movements are reliable for positioning the bed in people affected by advanced multiple sclerosis. Seven of 17 patients indicated that they would choose this system for bed control and wished to extend it for another application.

**Supplementary Information:**

The online version contains supplementary material available at 10.1186/s12984-023-01193-w.

## Background

Global estimates from 2010 show that more than one billion people suffer from some form of disability, equivalent to approximately 15% of the population. Of these people, 2–4% have significant difficulties in functioning [1]. In 2021, 53,700 patients in the Czech Republic were at the highest level IV (complete dependence on care) [2]. The last decade has seen an alarming increase in patients with upper limb disabilities [3].

Despite being bedbound, the cognitive functions of such people are generally preserved. They can perform PC-based work and manage some of their daily needs, thus improving their self-sufficiency and reducing the burden placed on families or assistants by advanced technologies [4–6]. Independence in the operation of an electrically powered and controlled positioning bed creates the opportunity to actively engage in communication and expand interaction with the environment, creating a comfortable position for rest and work. Therefore, research in this area is highly desirable. Different designs of electric actuators with varying functions of control meet the needs of a wide range of medical sectors [7]. For electric reclining beds, control is performed by a push-button controller [8].

However, muscle weakness or upper limb immobility rules out the push buttons as a suitable human–computer interface. In such cases, eye movements can be a communication tool [9], especially when a loss of speech (connected to pulmonary ventilation) significantly limits the use of voice [10] or gesture [6] control. Those who may benefit from an eye-controlled reclining bed can be found among patients with partial or complete spinal cord injury, amyotrophic lateral sclerosis, strokes, various muscular dystrophies, etc. [11], and also among people diagnosed with Multiple Sclerosis (MS). About 2.8 million people worldwide have this disease. The incidence of MS, according to the international project Atlas of MS (covers 115 countries of the world and approximately 87% of the world population), increased by 30% compared to 2013. In the Czech Republic, the number of MS patients is estimated at 23 thousand, and around 700 new patients have diagnosed annually [12–14].

This manuscript presents a solution for patients with partial to complete upper limb motor limitations. Using an eye-tracking and electromechanical device, we turned a button-operated reclining bed into a bed position control system using eye-tracking technology (BCET). We verified its functionality in a control group and then evaluated its usability in patients with multiple sclerosis who had significantly impaired upper limb motor skills.

## Methods

### Description of materials

#### Bed control system

**Fig. 1:**
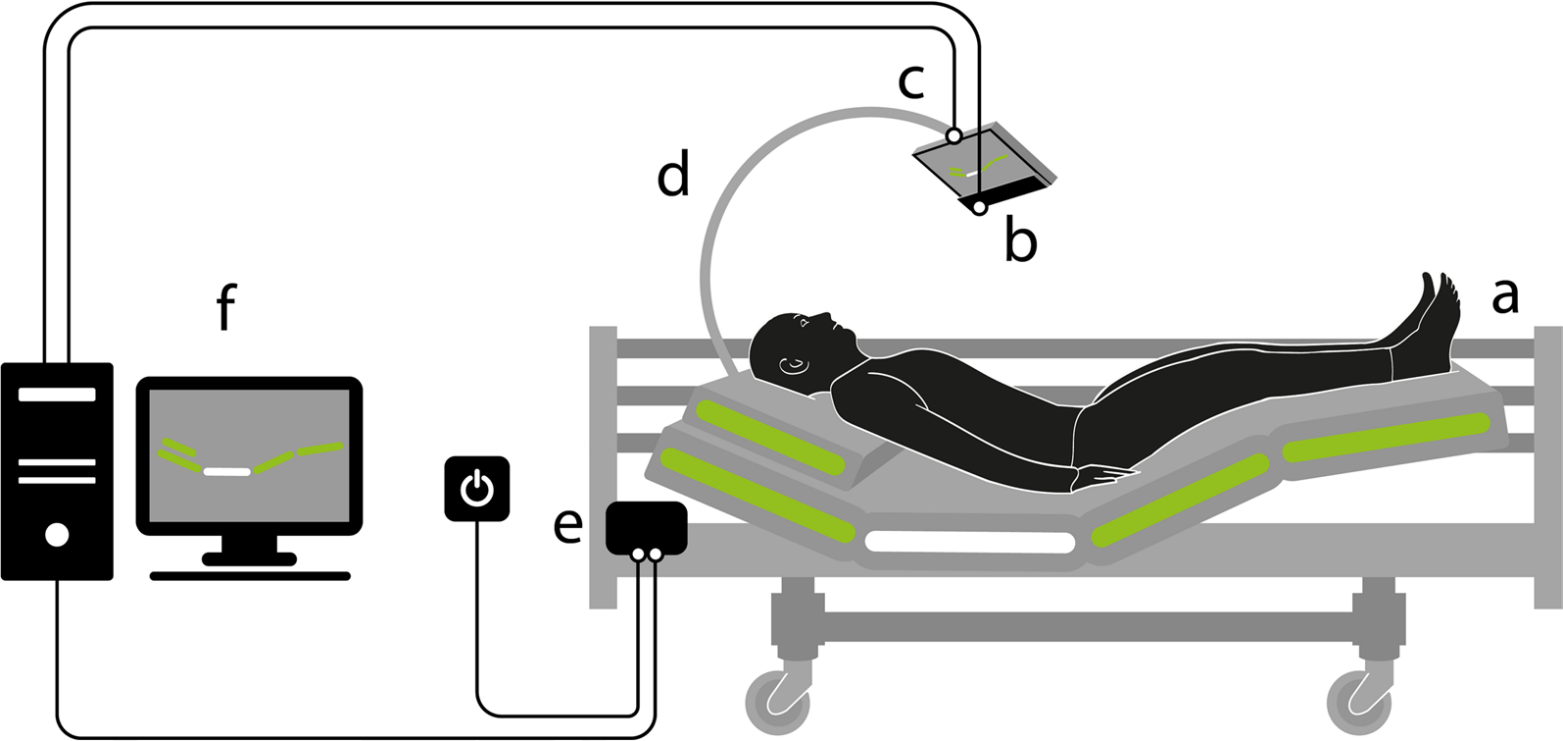
BCET consists of **a** positioning bed, **b** eye tracker, **c** control monitor, **d** flexible arm, **e** control unit and **f** desktop computer with operator monitor. The system is under patent protection CZ 309229

For this study, we selected the Latera commercial, electrically reclining hospital bed from LINET, Ltd. The bed had to be equipped with a display holder and an eye movement detector, as shown in Fig. 1, depicting the design of the BCET system. At the top of the headrest frame, a flexible mount (Fig. 1, Part d) was attached to the socket intended for a satellite panel. The flexible mount was a 150 cm (60 inches) long rod in which the metal core was encased in a plastic coating. This composite material allowed flexible position adjustment between the user and the display for the correct viewing distance and angle (from 20° to 90°) and bed transport.

The monitor (Fig. 1, Part c) with the eye tracker (Fig. 1, Part b) was attached to the bracket using a fixed axis rotation end joint fabricated using fused deposition modeling (FDM) 3D printing technology. We utilized a lightweight, 0.8 kg matte, 15.6″ ASUS MB168B LCD monitor with a resolution of 1366 × 768, an aspect ratio of 16:9, a refresh rate of 60 Hz, and a brightness of 200 cd/m^2^.

#### Control unit (hardware and software)

**Fig. 2:**
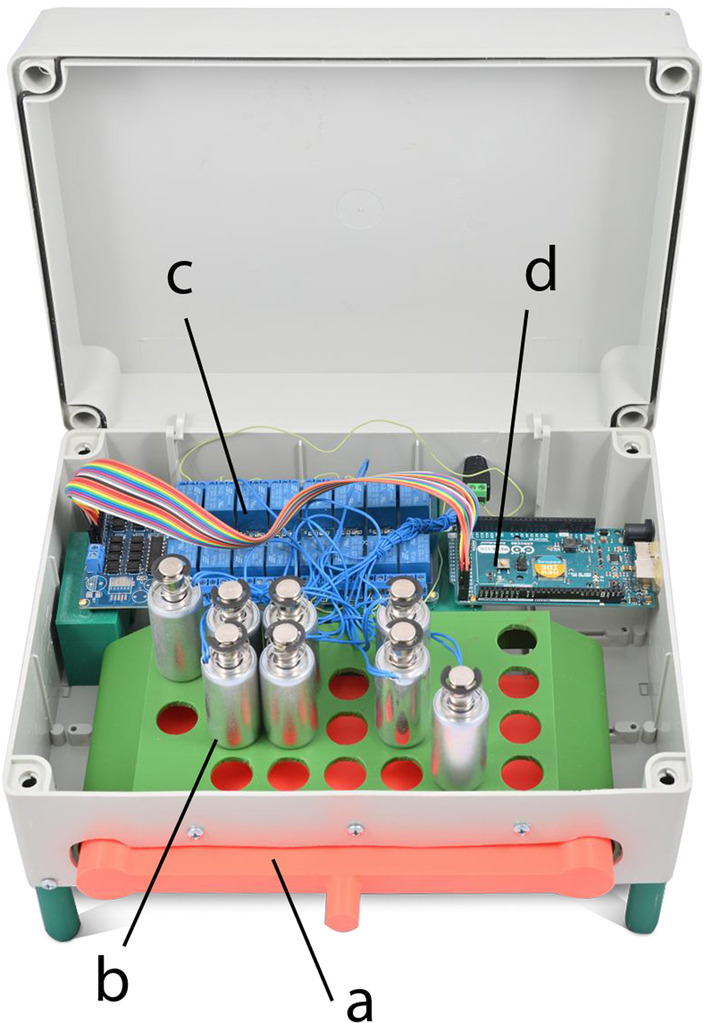
Control unit—an interface that pushes buttons of the remote control with **a** model of the inserted controller, **b** D/A converter module with solenoids, **c** relay systems, and **d** Arduino board

**Fig. 3:**
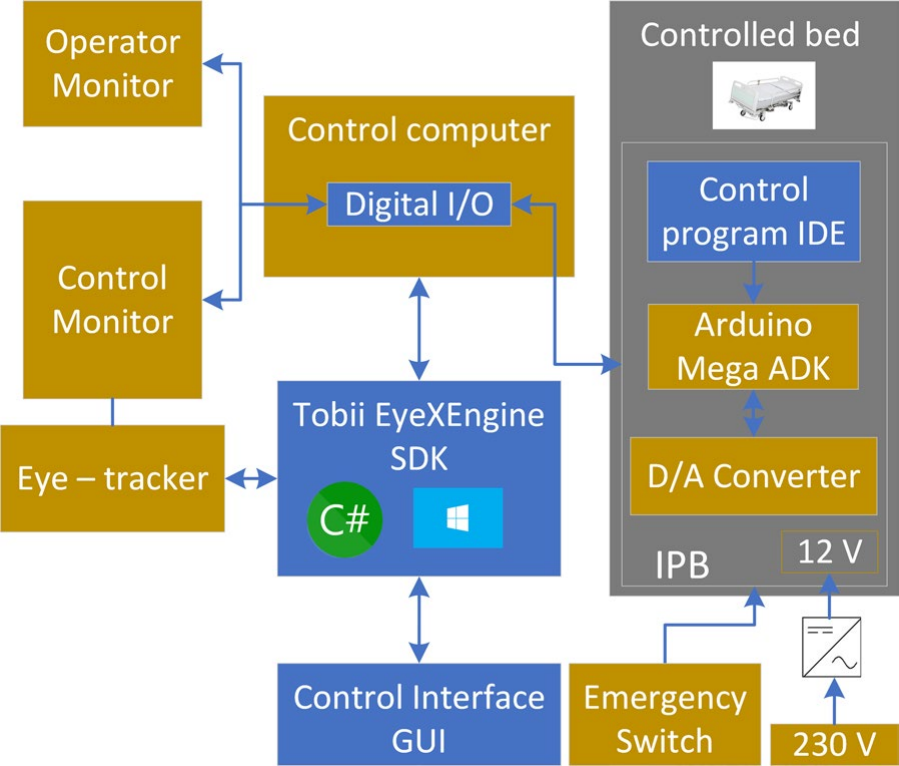
Block diagram of the experimental setup of BCET

The patient can position the bed using a handheld remote. The staff can use the Supervisor Panel, Foot Control, and an optional external Satellite Panel. It was impossible to change the bed control unit to implement BCET because of medical regulations. We therefore prepared an interface that pushes the buttons of the remote control. A large part of the interface was designed using the CAD program Autodesk Inventor Professional and was produced by 3D printing (Delta Q, TriLab) using FDM technology. The interface was fixed on the Supervisor Panel (Fig. 1, Part e). The digital signal to control the bed was processed by an Arduino Mega ADK (Fig. 2, Part d) and controlled an array of 16-channel relays (Fig. 2, Part c) and actuators in the form of push–pull solenoids (DC 12 V) with a maximum core extension of 10 mm and a push-end force of 21 N (Fig. 2, Part b). After a control pulse, these precisely centered actuators press buttons on the bed controller like a human operator. A force gauge experimentally measured the minimum pushing force to press the center button of the actuator to be 10 N. We chose to implement the system with twice the pushing force because of minor variations caused by the printing of the interface components and especially because the distance of the solenoid core from the actuator may reduce the reliability of transmitted commands. The BCET control architecture is shown in Fig. 3.

#### Eye-tracker

**Fig. 4:**
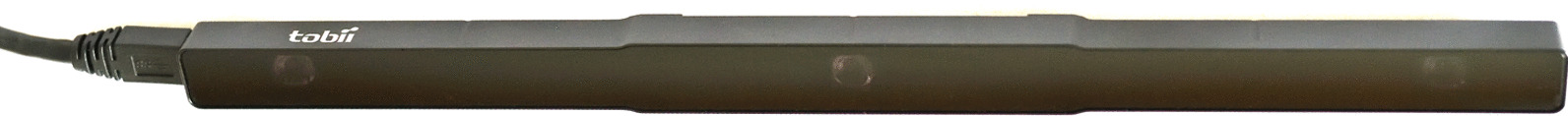
EyeX eye tracker

For eye-tracking, we chose the Tobii EyeX Dev Kit (Fig. 4), a developer’s binocular eye tracker with a basic set of software libraries designed for game and consumer application developers. Several techniques have been used to detect and track eye movements. One of the most common approaches is the pupil center/corneal reflection method [15]. The corneal reflection, produced by a near-infrared illumination source, and the pupil center are the input variables for estimating the gaze. The method assumes that the line of sight connects the center of rotation of the eyeball and the center of the pupil. In this method, the corneal reflex image center and pupil position move in tandem with head movement, so it is less susceptible to head movement. The EyeX device uses dark pupil tracking to locate the center of the user’s pupils and then calculates the gaze using the standard pupil center/corneal reflection method. Corneal reflection is produced by near-infrared illumination. EyeX has an accuracy < 0.6°, precision < 0.25°, latency < 50 ms, and a sampling rate of 60 Hz. These parameters were sufficient for our application [16]. The viewing distance of EyeX can be selected in the range of 450–800 mm (18 to 32 inches). The tracker allows free head movements. Depending on the user’s distance from the screen, the maximum allowed horizontal and vertical head movements are varied. The distance of the device from the observer in our experiment was approximately 750 mm (30 inches), and the maximum viewing angle was [− 18°, 18°] on the x-axis and [− 10°, 10°] on the y-axis, which fit within the limits given by the manufacturer. At this distance, we performed calibration, practice, and subsequent testing. The Tobii EyeX Engine performed gaze coordinate calculations on a PC (Windows 10 (64 bit) with an Intel Core i3-6100 processor with 8 GB RAM, an integrated graphics card, and a separate power supply). The tracker was connected to the PC via a USB 3.0 interface and mounted on the display. The Tobii EyeX Engine calibration procedure with or without glasses was used to increase accuracy with each new user [17]. During calibration, the user followed a calibration point on the screen.

#### Graphical visualization

We developed a custom graphical user interface (GUI) with virtual buttons to evaluate user commands and control the bed. In the following section, the GUI will always refer to the bed control environment and not to the operator environment. Due to the availability of libraries from the Tobii Software Development Kit (SDK) and the features needed to access data from the eye tracker, we chose the manufacturer-supported C# language to program the application. The application has three basic modes: standby mode, selection mode, and execution mode. The graphics are based on simple pictograms, and their size is maximized to be usable for lower visual acuity and less accuracy in guiding eye movements. Fresh green was chosen for active segments and the confirmation element. The inactive parts were white. The background was dark blue, and the outlines of the auxiliary lines were gray.

Eye-Tracking for control requires that the commands the person wants to execute are correctly identified in a continuous stream of gaze direction. The possibility that a gaze will cause unintended activations is called the Midas touch problem [18]. Usually, attempts are made to prevent these involuntary activations by using a blink sequence, dwelling the gaze in one place, or selecting a type of fixation [19]. Given patients’ varying degrees of visual impairment, we used a strategy of large control areas and relatively long dwellings in identifying their intentions when controlling the bed to prevent unwanted commands and injury. In our case, the smallest area of interest was 69 × 433 arc min with the white guided dot 8.59 arc min to select the positioning segment. The largest area (232 arc min) covered the central part of the display and served as the confirmation element. The minimum dwell time of the gaze on the element was 500 ms for the selection and 2000 ms for the confirmation. Because the time for confirmation was long, there was a possibility to deviate the gaze direction from the element for 0.5 s without interrupting the confirmation process, as described below.

**Fig. 5:**
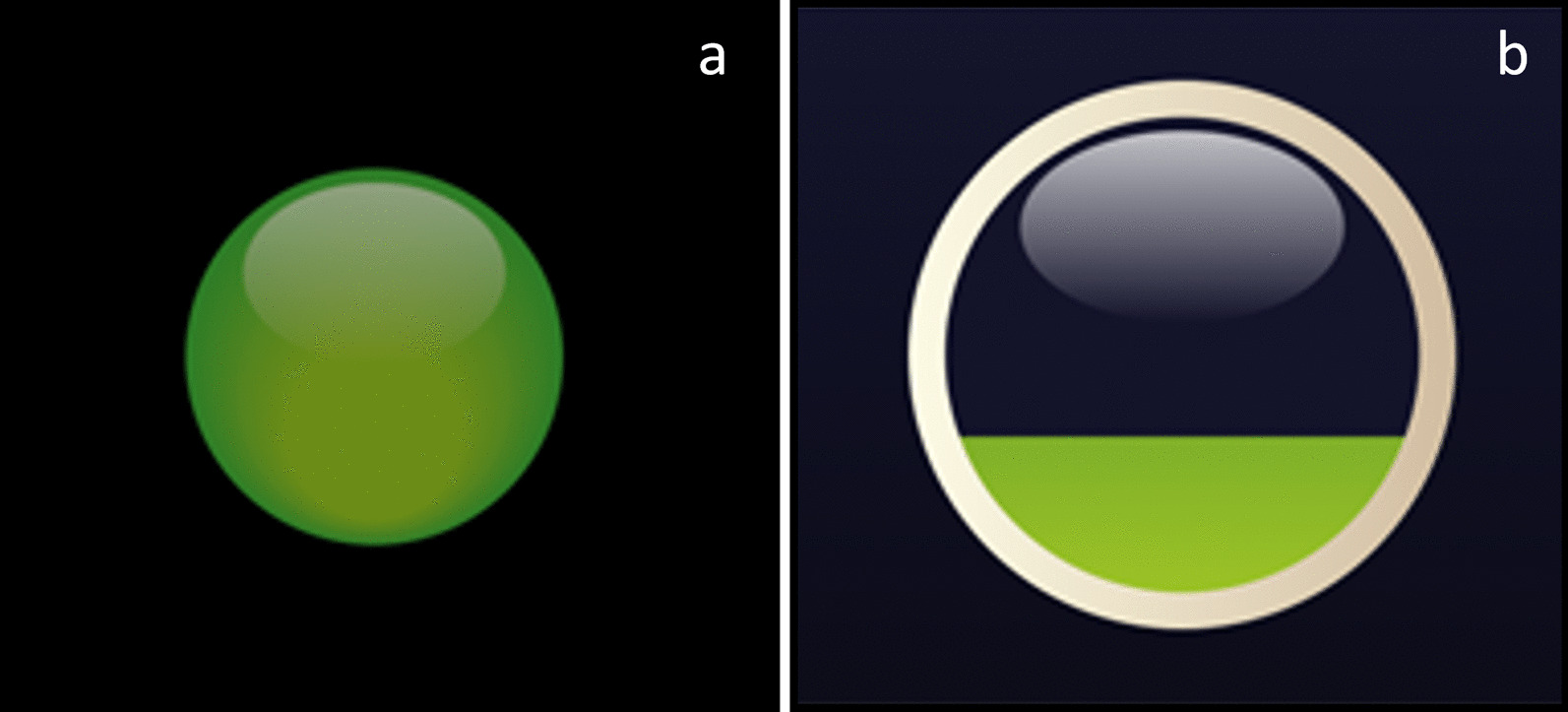
Initialization of the system. From the left, **a** the application is initiated by looking at the stand-by element (green pictogram) and **b** confirmation element

The standby mode is employed for long-term monitoring if there is an eye interaction with the display, i.e., it detects the user’s interest in working with the application. The central graphic element is a green shaded circle, resembling an LED periodically appearing on the GUI. During each period (11 s), the element gradually lights up (4 s) and off (4 s). Then, it is followed by an adjustable interval of a blank screen (3 s in our case). The application detects eye contact if the eye is fixed on the diode. Then, the application is converted to a confirmation element (Figs. 5b and 8)—an open circle. When the element is displayed, its background is grayed, and the user can focus solely on the element. Keeping the gaze in the circle, a green “liquid” gradually fills the element. Fixating the element for 2 s completes the confirmation process. If the user loses contact with the element for less than 0.5 s and manages to return the gaze to the element, the confirmation action continues. In our application, this element approves a selected action and substitutes the Enter key or the left mouse button.

The bed allows 14 positions. However, for safety, we chose to control the positions of the upper segment (headrest), lower segment (leg rest), and combined lower and upper segments (both headrest and leg rest). Additionally, we included a return of the bed to the position for cardiopulmonary resuscitation.

**Fig. 6:**
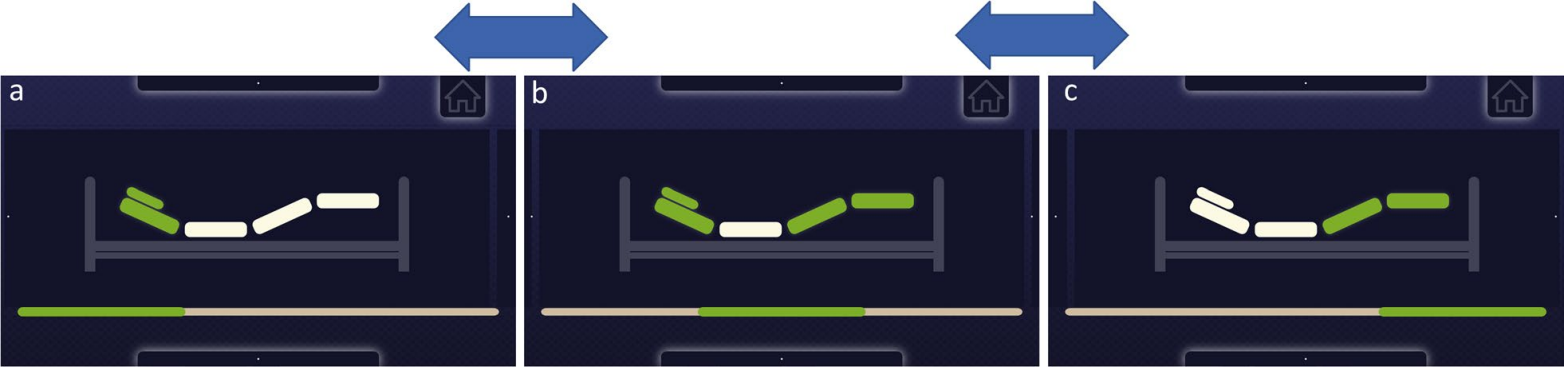
Control and selection of three position segments from the left: **a** headrest, **b** headrest and leg rest, and **c** leg rest

In the selection mode, the bed is symbolized by three horizontal segments (refer to Figs. 1 and 6). After exiting standby mode, the entry active position is the middle segment (Fig. 6b). Viewing the left panel with a dot changes the active position to the headrest (Fig. 6a), and viewing the right panel shifts activation to the leg rest (Fig. 6c). Scrolling the view to the opposite side returns to the previously selected segment. We set a minimum fixation time of 0.2 s for segment selection to minimize unwanted commands.

**Fig. 7:**
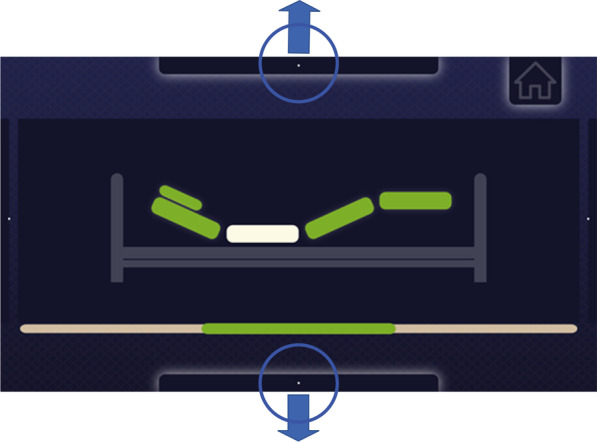
Raising/lowering the selected bed segment (green) to the desired angle, using the view of the selected bar with a guide dot

Looking at the bar with the white dot on top of the GUI activates the bed lift mode. The gaze on the bottom element of the GUI selects the downward motion—refer to Fig. 7.

**Fig. 8:**
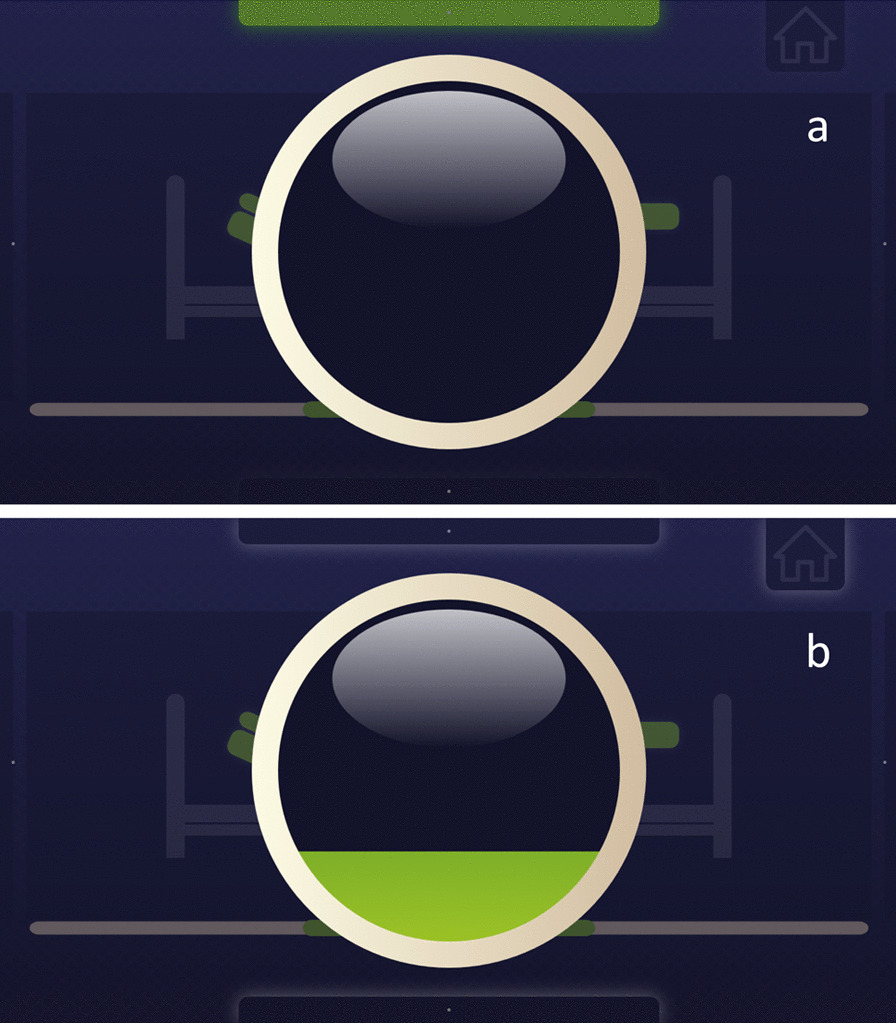
Simultaneous lifting of the headrest and leg rest using the confirmation element: **a** start and **b** lifting process (filling circle). The controlled bed part and direction are highlighted (green)

When the position and upward or downward change are selected, the execution mode is initiated, and the confirmation element is displayed. To raise the position, the element is filled from its bottom upward. To lower the position, the element is filled from its top downward. During the confirmation, the bed continuously changes in the selected position and direction. The positioning time depends on the will of the user. For safety reasons, a single positioning is limited to 3 s, which roughly corresponds to 30° of the bed segment change. After this time, the interface is automatically interrupted, and the bed movement stops. Additionally, when eye contact is lost, the activation or position change process is stopped (Fig. 8).

The Home button, located in the upper right corner of the GUI (Fig. 7), is part of the selection mode. When the user fixates the home button, the bed repositions to the lowest position and straightens for cardiopulmonary resuscitation. This selection mode was part of the experiment only during free practice and for transferring patients to a bed.

#### Settings of the study

##### Control group

Fifteen women and eleven men were recruited, and the mean age of the participants was 42.7 ± 15.9 years (SD). Testing took place in the medical biophysics laboratory, Faculty of Medicine in Hradec Kralove, Czech Republic. Selection criteria included physical and mental health.

##### Patient group

Nine females and eight males were selected. The mean age of the patients was 60.3 ± 9.14 years. Patients had to have preserved vision, the ability to move their eyes, and preserved cognitive function. Therefore, we selected clients of the facility who understood the experiment but had poor or no hand-motor skills, i.e., they were unable to operate a handheld remote or any conventional hospital bed positioner to control a bed with sufficient precision. A neurological scale of the EDSS [20] had to be greater than seven (ten means death due to MS).

These patients were in a wheelchair. To transfer them to a bed, they required an assistant, and in some cases, a lifting device. The patients had considerable difficulty with daily living activities and had no experience with the BCET system or similar types of control.

Therefore, the testing took place in a sanatorium specialized for people with MS in the St. Joseph’s Home in Žireč near Dvůr Králové, which is the only inpatient facility of its kind in the Czech Republic. The experiment was conducted in a 4 × 3 m rehabilitation room in the presence of 2 trained staff and one experimenter. The experiment could be interrupted at any time either by the operator using a safety button or PC or by the patient terminating the interaction with the display.

In either group, wearing glasses was not an exclusion criterion. The age and sex of the participants are listed in Table 1.

**Table 1:** Profile description of participants

Age					
Group	Sex	N	Median (years)	Low quartile (years)	Upper quartile (years)
Controls	F	15	34.0	21.0	49.0
	M	11	52.0	43.0	61.0
Patients	F	9	60.0	54.0	65.0
	M	8	63.5	61.5	67.3

Unfortunately, the groups are not age-comparable, and the entire BCET is no longer in use. We had to return the reclining bed, and we could not measure additional participants at this time. For this reason, we present descriptive characteristics and training/learning effects on all groups (26 control participants and 16 patients). To compare the results between the group of controls and patients, we defined subgroups so that their age was not significantly different, and the number of observations was sufficient to assess the difference. We achieved these requirements by restricting the age of the groups to the interval from 40 to 65 years. Subgroups comprised eleven patients (five men and six women, mean age 51 years) and twelve controls (six men and six women, mean age 56 years).

#### Experimental design

The experimental design was the same for the control and patient groups. Participants read the informed consent form, which was supplemented with a pictorial manual (see Additional file 1). Any ambiguities were answered by the experimenter (MK). Only participants who signed the informed consent form were included in the experiment.

At first, all participants underwent eye tracker calibration (it could be repeated three times). Participants who successfully passed the calibration were familiarized with the application under the operator’s (MK) guidance (approximately 10 min). The practice was followed by the test, which consisted of executing a sequence of commands: (a) initiate eye contact with the application; (b) raise the leg rest (for 2–3 s); (c) raise the headrest (for 2–3 s); (d) lower the leg rest (for 2–3 s); (e) lower the headrest (for 2–3 s); and (f) break eye contact with the application. Each test was repeated thrice with short pauses to lower task-solving variability and allow for learning or fatigue assessment.

**Fig. 9:**
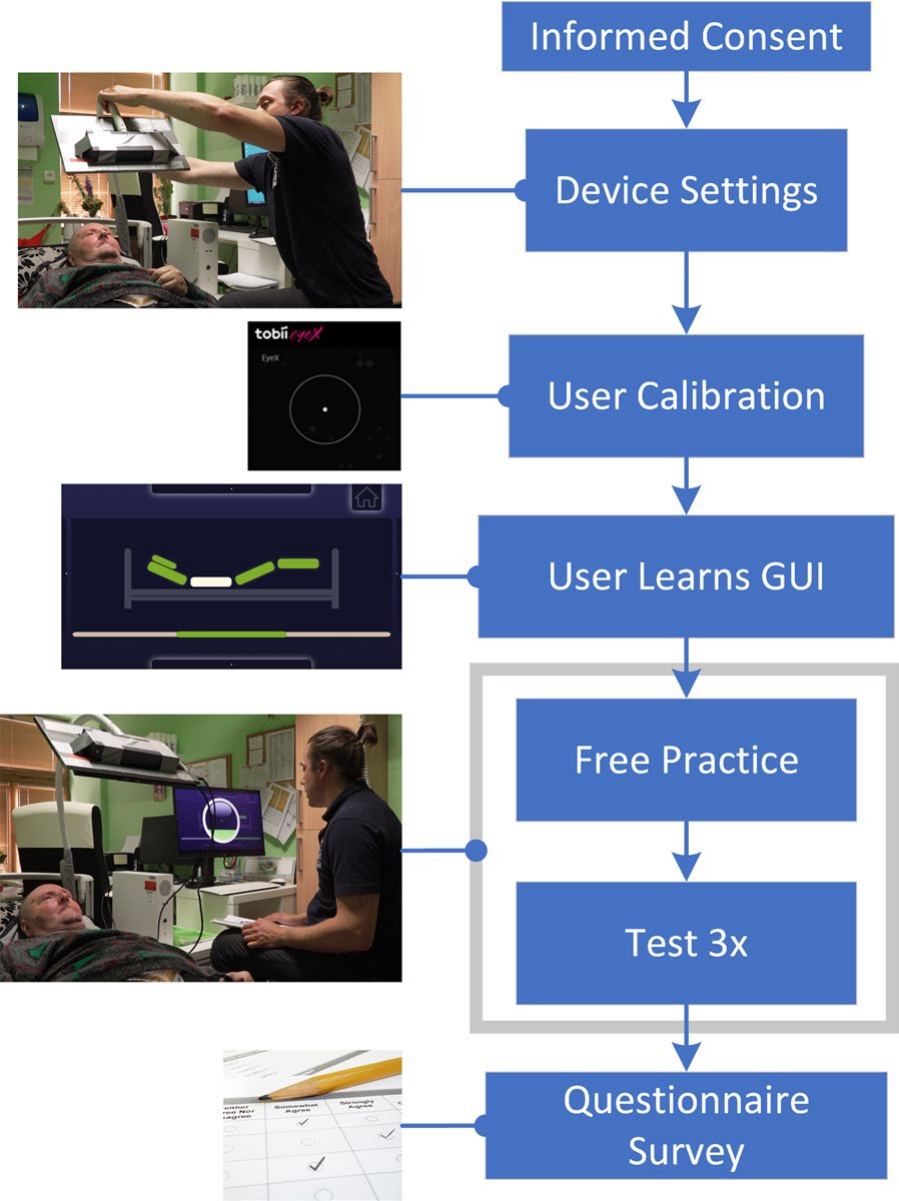
Sequence of the experiment from the user’s perspective. The free practice and the test accompanied by a photo of a workplace at St. Joseph’s Home in Žireč with the ongoing experiment. The photo depicts the patient, the experimenter, and the BCET

The duration of each test was approximately 60 s. The operator led participants through the test announcing the upcoming step of the sequence. Three individuals refused the support and performed the test without the operator leading. A questionnaire survey immediately followed completion of the tests. The entire session was approximately 30 min (including experimental setup and breaks). A schematic of the experiment is shown in Fig. 9.

#### Analysis

To evaluate the test solving, we used the event log recorded by the application. The test log contained timestamps for each operation. From the log, we determined the total number of steps, time to complete the test, and time from the first contact to the first positioning (time to first positioning). For complete log, see Additional file 2. The critical parameter for measuring the BCET utility was the time to complete the test. However, this time included positioning (up/down), the duration of which was individually determined by the volunteer and was thus random. We subtracted the positioning time from the total time to minimize the random part. This adjusted time (task time*) was employed for the following analyses.

To evaluate the information data rate (ITR) of BCET, we applied a similar approach as used for the brain-computer interface (BCI) [21, 22]. For each test step, we described the number of states the volunteer could choose from and converted them to bit form. We divided the sum of all bits for a given test by the difference in the time spent in the decision sequence without security delays. The difficulty of the decision process varied across steps because the interface had different complexities. Table 2 details the sequence and the number of states from which the volunteer could choose. During one test, the volunteer could choose from 91 states, which corresponds to 51 bits to transfer.

**Table 2:** Optimal method of solving the test with the enumeration of decision levels at each step

1.	Start of communication with confirmed choice (2 states/1 bit)
2.	Selection mode—headrest, both, leg rest, up, down, reset, end (6 states/3 bits)
3.	Selection mode—both, leg rest, up, down, reset, end (5 states/3 bits)
4.	Selection mode—up, down, both, reset, end = (5 states/3 bits)
5.	Execution mode (2 states/1 bit)
6.	Selection mode—both, leg rest, up, down, reset, end (5 states/3 bits)
7.	Selection mode—headrest, both, leg rest, up, down, reset, end (6 states/3 bits)
8.	Selection mode—headrest, both, up, down, reset, end (5 states/3 bits)
9.	Selection mode—up, down, both, reset, end = (5 states/3 bits)
10.	execution mode (2 states/1 bit)
11.	Selection mode—headrest, both, leg rest, up, down, reset, end (5 states/3 bits)
12.	Selection mode—headrest, both, leg rest, up, down, reset, end (6 states/3 bits)
13.	Selection mode—both, leg rest, up, down, reset, end (5 states/3 bits)
14.	Selection mode—up, down, both, reset, end = (5 states/3 bits)
15.	Execution mode (2 states/1 bit)
16.	Selection mode—both, leg rest, up, down, reset, end (5 states/3 bits)
17.	Selection mode—headrest, both, leg rest, up, down, reset, end (6 states/3 bits)
18.	Selection mode—headrest, both, up, down, reset, end (5 states/3 bits)
19.	Selection mode—up, down, both, reset, end = (5 states/3 bits)
20.	Execution mode (2 states/1 bit)
21.	Return to stand-by mode (2 states/1 bit)

The optimal solution of the test required 21 steps. The number of extra steps required by the participants reduced the solution efficiency, which we calculated as the optimal number of steps times a hundredth divided by the number of steps completed by the proband. If the number of steps for the solution was equal to 21, the efficiency was 100%.

We analyzed whether there was a learning effect during task repetition in the test execution time and efficiency. We calculated the slope of the regression line, which we hereafter refer to as the trend among three values for each volunteer. The execution time or efficiency represented the dependent variable, and the test order represented the independent variable. The trends were then compared by a one-sample test against zero.

Subjective experience with the system and test flow was obtained from a questionnaire survey focusing on confidence in control, satisfaction with system activation, understanding of how the bed segments were symbolized, clarity of control, and preference of BCET to another imaginary control. Responses were on a 5-level scale from strongly disagree/dislike to strongly agree/like. On a 6-point scale, participants expressed their pain intensity during the task. Habitual visual attention was assessed by asking about the ability to watch a movie. For all questionnaires, see Additional file 3.

The Shapiro‒Wilk test for normality of data distribution was performed before statistical comparison. As the test rejected a normal distribution in a range of parameters, the Mann‒Whitney test was performed to compare groups. We employed Spearman’s correlation test to assess the degree of association among the parameters of interest. The significance level was 5% in the tests performed. For statistical processing and evaluation of the measured data, we selected the Jamovi tool [23] and Microsoft Office 365.

## Results

The study was approved by the local ethics committee of the University Hospital Hradec Králové, Czech Republic (201411 S20P) and conducted in accordance with the Declaration of Helsinki. A total of 43 participants took part in the experiment and performed 126 tests.

In one patient, no follow-up testing was performed due to the absence of gaze detection. Three patients and nine participants in the control group completed the test with glasses or contact lenses. The minimum task time* for passing the individual test with the optimal strategy (21 steps) was 23.9 s, and the longest individual time was 187.4 s (41 steps). No volunteer completed all three tests without redundant interactions, and only a few controls achieved an efficiency of 100% in single tests. For descriptions and comparisons of performance between the groups, we calculated the average of each volunteer’s three repeated tests. The results of the full groups are shown in Table 3.

**Table 3:** Descriptive characteristics of monitored variables for all participants divided into patients and controls

	Group	N	Percentiles				
			Median	25th	75th	Min	Max
Task time* (s)	Controls	26	40.2	34.5	45.5	25.7	66.7
	Patients	16	56.5	46.5	64.9	38.2	134.3
Efficiency (%)	Controls	26	86.3	81.6	91.0	67.0	96.9
	Patients	16	72.1	63.0	75.2	57.3	84.0
EDSS (–)	Controls	0					
	Patients	17	9.0	8.5	9.0	7.0	9.5
Time to first positioning (s)	Controls	26	11.5	10.2	14.1	9.6	20.6
	Patients	16	13.9	12.4	16.5	10.9	23.3
Age (years)	Controls	26	46.5	30.3	55.0	19.0	72.0
	Patients	17	63.0	57.0	67.0	42.0	73.0
ITR (bits/s)	Controls	26	1.5	1.3	1.8	0.8	2.5
	Patients	17	1.0	0.9	1.2	0.4	1.6

The distribution of the observed parameters for age-matched control and patient groups is described by the median, lower and upper quartiles, and minimum and maximum. Task time* excluded the rising and falling passages in the bed setting, which were not constrained by the test and could be performed differently by each person

Controls mastered the task in 40.2 (34.5 and 45.5) s [median (lower quartile and upper quartile), respectively], and the results were relatively consistent. In the patient group, the total time of 56.5 (46.5; 64.9) s had considerable variability: from a minimum time of 38.2 s to 134.3 s. In the patient group, the task solving efficiency 72.1 (63. 0; 75.2)% was lower than that in the control group, 86.3 (81.6; 91.0)%. The controls managed the BCET system initialization and the first positioning in 11.5 (10.2; 14.1) s, and the patients managed those in 13.9 (12.4; 16.5) s. This measurement indicates that establishing interaction with the system, confirming safety procedures, and opening the function selection were seamless for both groups.

In age-matched subgroups, the controls had a task time* of 17.7 s shorter (p = 0.009) and had 12.1% higher efficiency (p = 0.001) than the patients, and the time from making contact with the system to the first positioning was shorter by 1.7 s (p = 0.050). The ITR was significantly (p = 0.009) higher by 0.5 bit per second in the healthy volunteer group than in the patients, which directly corresponds to the task time*. The details are listed in Table 4.

**Table 4:** Descriptive characteristics of monitored variables for age-matched control and patient groups

	Group	N	Percentiles					
			Median	25th	75th	Min	Max	p
Task time* (s)	Controls	12	40.5	37.5	44.5	25.7	66.7	**0.009**
	Patients	10	58.2	45.9	65.1	38.2	134.3	
Efficiency (%)	Controls	12	84.6	79.0	90.3	75.0	92.7	**0.001**
	Patients	10	72.5	64.6	75.7	61.2	84.0	
EDSS (–)	Controls	0						
	Patients	11	9.0	9.0	9.0	7.5	9.0	
Time to first positioning (s)	Controls	12	12.4	10.4	14.0	9.7	20.6	**0.05**
	Patients	10	14.1	12.5	18.5	12.0	23.3	
Age (years)	Controls	12	50.0	48.8	56.0	41.0	59.0	0.1
	Patients	11	58.0	50.5	63.0	42.0	64.0	
ITR (bits/s)	Controls	12	1.5	1.3	1.6	0.8	2.5	**0.009**
	Patients	10	1.0	0.9	1.3	0.4	1.6	

The significant p-values are printed in bold

The parameters and their descriptions are analogous to those in Table 2. The p value of the nonparametric Mann‒Whitney for the independent observations test compares both groups and indicates a deficiency in the task time*, time to first positioning, efficiency of task solving, and the ITR for the patient group

We analyzed whether there was a learning effect when the task was repeated. We chose the slope of the regression line for the evaluation (see “Methods”). Learning was evident in the patient group for task solving time* (p = 0.021) and for efficiency (p = 0.006). Patients improved on average by 7.5 s (95% CI − 15.7, − 0.3 s) and 5.4% (95% CI 1.7, 9.5%) on each test. Participants in the control group did not show this significant trend. The time from task opening to first positioning did not change with repetition for neither group (refer to Table 5).

**Table 5:** Trends for measured variables in patients and controls

	p	Slope	Lower 95% CI	Upper 95% CI
Patients				
trend_Time to first positioning	0.404	− 0.658	− 6.590	0.621
trend_Task time*	**0.021**	− 7.524	− 15.720	− 0.276
trend_Efficiency	**0.006**	5.387	1.670	9.468
Controls				
trend_Time to first positioning	0.111	− 0.524	− 1.260	0.139
trend_Task time*	0.123	− 1.368	− 2.980	− 0.461
trend_Efficiency	0.270	1.598	− 1.240	4.318

The significant p-values are printed in bold

The trend separately describes the slope of the linear regression and its 95% confidence interval over three tests for each volunteer for the control and patient groups. The p value indicates whether the estimate of the slope is significantly different from zero (Wicoxon rank test). During test repetition, the task time* was significantly reduced, and the efficiency improved in the patient group

In the correlation analysis for the patient group, we found that a higher degree of disability (EDSS) was significantly related (rho = − 0.59, p = 0.017) to slower growth in efficiency throughout testing. We did not observe associations of any of the analyzed parameters to age. For within-test correlations, there was a significant positive association of test time to efficiency (rho = − 0.54, p = 0.030) and time to first positioning (rho = 0.54, p = 0.035). We found a significant relationship between improvement in task time* and time to first positioning (rho = 0.74, p = 0.002). We also observed that the time to the first positioning was related to learning expressed by the trend of the test time* (rho = − 0.59, p = 0.019) and the trend of the time to the first positioning (rho = − 0.59, p = 0.019). The strength of all correlations performed is shown in Table 6 below the diagonal.

**Table 6:** Correlation matrix of relationships among the observed parameters

	Task time*	Trend task time	Time to first positioning	Trend time to first positioning	Efficiency	Trend efficiency	Age
Task time*	–	0.043	0.623***	0.169	− 0.175	0.062	0.531**
Trend task time	− 0.118	–	0.267	0.084	− 0.346	− 0.353	− 0.198
Time to first positioning	0.535*	− 0.588*	–	0.249	− 0.154	− 0.134	0.365
Trend time to first positioning	− 0.400	0.735**	− 0.588*	–	− 0.210	− 0.118	0.283
Efficiency	− 0.542*	− 0.075	− 0.392	− 0.053	–	− 0.025	0.428*
Trend efficiency	0.159	− 0.382	0.371	− 0.376	− 0.326	–	0.211
Age	− 0.053	0.111	− 0.059	0.096	− 0.236	0.271	–
EDSS	0.282	0.220	0.154	0.088	− 0.004	− 0.587*	− 0.260

Correlations for the patient group and control group are shown below the diagonal and above the diagonal, respectively. The correlations were calculated using Spearman’s nonparametric test. The significance of the relationship is marked by stars: *p < 0.05, **p < 0.01, and ***p < 0.001

In the control group, we found a significant association between increasing volunteer age and longer task time* (rho = 0.53, p = 0.005) and reduced efficiency (rho = 0.43, p = 0.029). Among the other tests, only the association between task time and time to first positioning were significant (rho = 0.62, p < 0.001). The strength of all correlations performed is shown in Table 6 above the diagonal.

In our experiment, the calculated median of the ITR was 1.5 (1.3; 1.8) bits/s for the control group and 1.0 (0.9; 1.2) bits/s for the patients. The ITR was affected by the implementation of time delays between some sequences. Time delays were introduced to limit unwanted user commands generated by nonintentional eye movements. For example, a 2 s delay for interrupting the standby mode or 0.2 s for selecting bed segments—the selection mode and 0.5 s for execution mode. For complete results, see Additional file 4.

In a questionnaire completed after the BCET tests, all participants confirmed that they could watch a full-length film without problems (14 patients and 26 controls) or with breaks (3 patients).

Positive responses were predominant when assessing the BCET features (confidence in control, activation, information on bed setup, position selection, and clarity of control), especially in the control group. None of the groups negatively rated the device, and the number of neutral rating answers was 11/181 in the control group and 10/114 in the patient group.

**Fig. 10:**
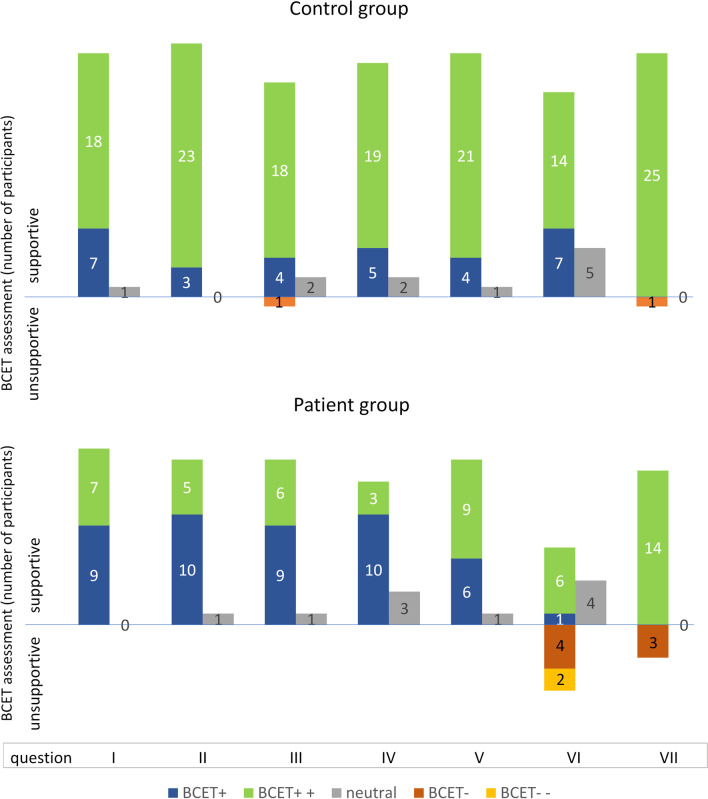
Questionnaire responses in the control group (n = 26) and patient group (n = 16). In the graph, each question is represented by bars with color-coded answers. The numbers of positive (negative) ratings are shown on the positive (negative) vertical axis. Columns I–V show the ratings of the BCET features: confidence in control (I), activation (II), information on bed setup (III), position selection (IV), and clarity of control (V). Positive ratings predominate in these categories in both groups. Six of 16 patients indicated a preference for an alternative control over the presented one (column VI). Pain during the testing (column VII) was indicated by a total of 4 participants (1 control and 3 patients) and rated as mild. The answers were on a 5-level scale: strongly supportive for BCET (++, green bars), likely supportive (+, blue bars), not sure (gray), likely unsupportive (–, orange bars), and strongly unsupportive (–, yellow bars)

In the question examining the preferred way to control the bed, patients, concerning their health condition, suggested solutions for bed control using voice, a balancing balloon in front of the mouth—a mouth mouse. Seven patients preferred the tested BCET over another control method, six did not and four were unsure*.* Three patients and one control person experienced mild pain during the test. The frequencies of responses to each question are summarized in Fig. 10.

## Discussion

The experimental approaches to foster a smoother and more seamless integration of user and assistive technology can be divided into three nonmutually exclusive areas [24]: (1) improved assistive technology mechanics, (2) improved user and physical interface, and (3) improved shared control between the user and technology. We incorporated these approaches into our design and subsequent implementation of the BCET and sought to effectively reduce the burden of long-term care. Our eye-tracking method have been compared with some references. A similar solution included a research team focused on bed positioning control and medical staff summoning [25]. They implemented external actuators in a standard mechanical bed with a GUI and eye tracker. This work was limited to the design of the control architecture and testing the functionality of the contactless positioning mechanics. In our case, we chose an additional external module (Fig. 2) that did not compromise the integrity of the bed control. We identically implemented the control of the bed position by the Arduino unit. The usability and efficiency could not be compared, because relevant data were not available in their study.

Atasoy et al. [9] used a webcam for eye-tracking to control a hospital bed with four motors in eight directions. They tested the interface’s usability on 30 subjects aged between 18 and 70 years without specifying their health status. The system, which did not require calibration, worked reliably in 90% of the subjects when the person’s distance from the camera was less than 500 mm. In our case, the distance was 750 mm because the subjects could not observe their surroundings and felt uncomfortable at closer distances. There was only one patient in our cohort of 43 subjects (2.3%) for whom BCET could not be used because the eye tracker could not detect her pupils.

In a questionnaire survey, Atasoy et al. found strong agreement that the system was not complicated, was stable, and close viewing distance was not disturbing. Survey responders were less clear regarding the ease of use and learning to operate their system. Positive experience with the technology and its function was also prevalent in our survey, yet when we explicitly asked whether respondents would prefer this option for bed control, the response was not as clear—see “Results”. We believe that testing appropriate patients and properly worded questions can contribute to developing and using eye-tracking technology.

A different approach to communication between the application and the patient was chosen in the study [26]. The system successfully measured and extracted signals related to visual stimuli from the electroencephalographic activity, and medically indisposed patients were able to control the required functions themselves. Before the experiment, the patients received instructions from the staff on how to use the system for half an hour, as in our study. More than 80% of patients from a questionnaire investigation found the system useful. It is comparable to and slightly lower than the assessment of our BCET functionality (questions I–V). They also measured ITR and gained 34.6 bits/min (i.e., 0.58 bits/s), which is lower than for our BCET (1.5 bits/s for controls and 1.0 bits/s for patients). In the BCET design, we excluded the registration of electroencephalographic activity because it requires the long-term mounting of electrodes, which is inconvenient and very difficult to implement in our patients. Another limitation is that patient movement generates extensive electrical artifacts. These artifacts must be removed to avoid limiting the patient. This situation is not trivial.

One parameter that allows a comparison of different systems is the data transfer rate. The ITR determined for controls by us 1.5 (1.3; 1.8) bits/s and patients 1.0 (0.9; 1.2) bits/s corresponds to a similar eye tracker system and task [27]. The authors achieved an ITR ranging from 1.9 to 2.5 bits/s, which they increased by adding the brain’s electrical activity to their system. Such a solution has the limitations mentioned above. Using BCI alone resulted in an ITR in the range of 0.33–0.45 bits/s [26]. Our results showed that eye-tracking-based systems have a higher ITR than BCI systems alone.

The limitation of eye-tracking is due to the user having direct visibility of an eye-tracking system containing an infrared light source and camera. The reliability of the systems is dependent on sufficient pupil view by the eye tracker and is reduced by reflections, camera glare, incorrect facial position, rimmed glasses, or visual disorders such as strabismus [28]. In our cohort, one patient with significant ptosis did not establish communication with the BCET because overly closed eyelids prevented sufficient pupil exposure. The manufacturer of eye trackers also points out this problem on their website [29]. We tried to minimize this problem by adjusting the sensor on the positioning arm to the position relative to the eyes according to the eye tracker documentation. Based on our knowledge of BCET development and in comparison with [28] and [30], we believe that the sampling rate of 60 Hz, calibration, and positioning of the eye tracker are optimal for the task at hand.

In our study, we observed better results for all observed parameters in the age-paired control group. The results are unlikely to have been significantly influenced by the patient’s pain, as only three patients in the questionnaire survey reported experiencing mild pain during testing. However, these patients may have been affected by analgesics, which may have caused a slight variation in accuracy and cognitive function [31–34].

Overall experimental time may also have been affected due to patients’ different tolerances to exertion, as discussed in [35], which investigates this issue for different degrees of immobility within the EDSS scale, confirming this finding. In our case, we tried to correct the patients’ different levels of physical exhaustion with two minutes of rest between each test. We found no significant correlation between test time and EDSS.

As a possible explanation, the task may have been influenced by the positioning of the proband in the supine position relative to the sensor, as presented in Fig. 9. Controls moved to the ideal viewing distance and angle relative to the sensor [36], and patients were affected by impaired motor skills.

As shown in many studies, age and declining cognitive capacity may have an equally important role in the change in testing time. The most important changes in cognitive function with normal aging are decreased performance on cognitive tasks that require rapid processing or transformation of information to make decisions, engaging working memory and executive functions [37, 38].

In our experiment, the learning effect was significant and evident in the group of patients in whom test repetition caused a decrease in the time to solution efficiency. We did not observe this effect in the control group. Participants probably achieved near-optimal results in the first test, and further improvement was not demonstrable.

It is clear from the literature that training eye movements using eye-tracking has other benefits. Children who received eye-tracking training showed better memory and faster learning [39]. Another study [40] that investigated stroke patients’ cognitive abilities showed that eye-tracking training significantly improved visual attention. It is possible that longer-term use of the BCET system by patients can also provide benefits at the level of visual and cognitive function.

An important part of the experiment was the subjective statements of the participants about their experience with the BCET. The results from the questionnaire were dominated by positive evaluations of the BCET, especially among the participants in the control group, who did not find it difficult to use.

Patients’ statements may have been influenced by slightly worse test results. The most varied responses were to whether they would choose any existing bedside controls over BCET. Seven patients (six definitely and one probably) would choose BCET as their preferred method of bedside control. Six patients did not prefer the BCET system: three of them chose hand control because they had retained sufficient motor skills to press the buttons, one patient suggested the use of voice control because he was familiar with the technology, another patient would use either voice or hand control, and the last patient was unable to complete the calibration. Four remaining patients were unsure of their preferences.

In an open questionnaire statement, some patients indicated that they would like to control actions using the BCET beyond bed positioning. They mentioned controlling the TV, radio, summoning an assistant, dialing contacts on a mobile phone, controlling blinds, lights, and air conditioning but also switching the pool filtration or reading an e-book. These applications have already been investigated [3, 5, 6] and might be incorporated into BCET in the future.

Some participants rated the colorful application setup (refer to Fig. 10) as very good (question V). Some studies have investigated the impact of green, blue, and gray colors [41–43]. Green is often associated with cognitive restorative effects [44], creativity [42], and safety [45]. Blue is often associated with comfort and calmness. Gray can be optimally matched for chromaticity and lightness [46].

In the future, it would be advisable to automate the system so that no external operator is needed and so that the system works autonomously according to the user’s commands and to extend it with additional features to increase the comfort of the bedside stay. Based on the results, we believe that BCET has the potential to increase the level of self-sufficiency and quality of life of patients with multiple sclerosis.

### Limitations

Although the study provides several answers regarding the feasibility and utility of bedside control using eye movements in patients with multiple sclerosis, it leaves some questions unanswered. For example, the study does not provide information about usability in individuals with other neurological diseases, such as dementia, mild cognitive impairment, or traumatic brain injury, possibly in patients after spinal cord injury. Additionally, a longer period of follow-up and use of BCET would allow for a full evaluation of the effect of training, improvements in activities of daily living and quality of life.

## Conclusion

Using custom software combined with components available on the market and a specially designed adapter, an add-on device was produced that can control the Latera positioning bed using eye movements. The user interface based on large elements with intuitive graphical meaning and the robustness of the eye movement detection were positively evaluated in terms of confidence and ease of use by both the control and the multiple sclerosis patient groups.

Important outcome of our study is that out of 16 patients who could successfully control the bed by sight, seven patients would use the technology. These were those who could not anyway operate the bed’s buttons manually. The preferences in the patient group contrasted with the group of healthy volunteers. They would all have used the device. The fundamental difference in device usability scoring needs to be considered in future studies testing the assistive technology. The results of healthy controls cannot be easily extrapolated to patients.

Participants in our control group performed the test sequence faster and more efficiently than patients. However, patients tended to improve with repetition in both parameters. The highest information transfer rate was 1.6 bits/s for the patients and 2.5 bits/s for the control group. The evaluation of efficiency in control by the patients showed that a higher disability level (EDSS) negatively correlated with efficiency. Nevertheless, all patients for whom eye movements could be registered successfully completed the test sequence. The eye-tracking was not possible in only one patient.

Based on the results achieved, we believe that BCET has the potential to increase the level of self-sufficiency and quality of life of patients with multiple sclerosis.

## Supplementary Information

**Additional file 1.** Instructions for contactless control of the adjustable bed application. Before starting the individual task, the volunteer was familiarized with the basic instructions for operating the positioning bed using a picture manual.

**Additional file 2.** LOG of patients and controls. Complete event log with described parameters evaluated during tests.

**Additional file 3.** List of questionnaires patients and controls. A sample of translated questionnaire is on the first page of the file, followed by scanned anonymized questionnaires.

**Additional file 4.** All measured and evaluated experiment data. Complete dataset for patient and control group.

## Data Availability

All data generated or analyzed during this study are included in this published article and its additional files.

## Abbreviations

EDSS: Expanded Disability Status Scale
HVAC: Heating, ventilation and air conditioning
BCET: Bed position control system using eye-tracking
FDM: Fused deposition modeling
CAD: Computer-aided design
GUI: Graphical user interface
SDK: Software Development Kit
MS: Multiple sclerosis
BCI: Brain–computer interfaces
ITR: Information data rate (information transfer rate)
