# Supplementary material for: Eye-tracking control of an adjustable electric bed: construction and validation by immobile patients with multiple sclerosis
Source: J Neuroeng Rehabil. 2023 Jun 9;20:75. doi: 10.1186/s12984-023-01193-w (PMC10251586; doi:10.1186/s12984-023-01193-w)

Instructions for contactless operation of the adjustable bed application

Standby mode	Confirmation element

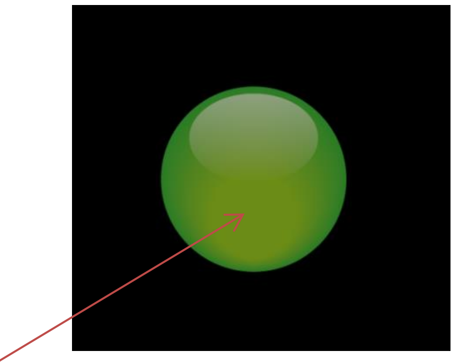

Starting the application
Looking at the flashing green ball, a confirmation element is generated.

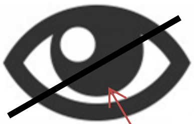

Interrupting the view returns the application to waiting

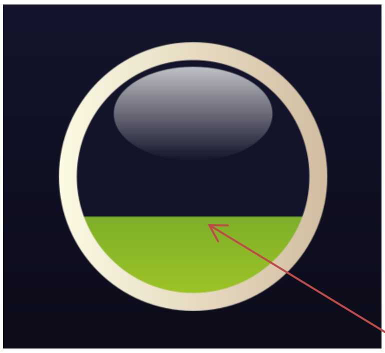

Looking at the center of the element starts its filling and then opens the segment selection (change of pos 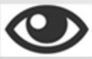

Location selection

By looking at the top and bottom dots, we control the positioning to raise/decrease the angle of adjustment of the Bed.

By keeping the gaze on the confirmation element, we change the positioning. 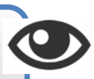

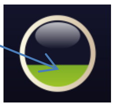

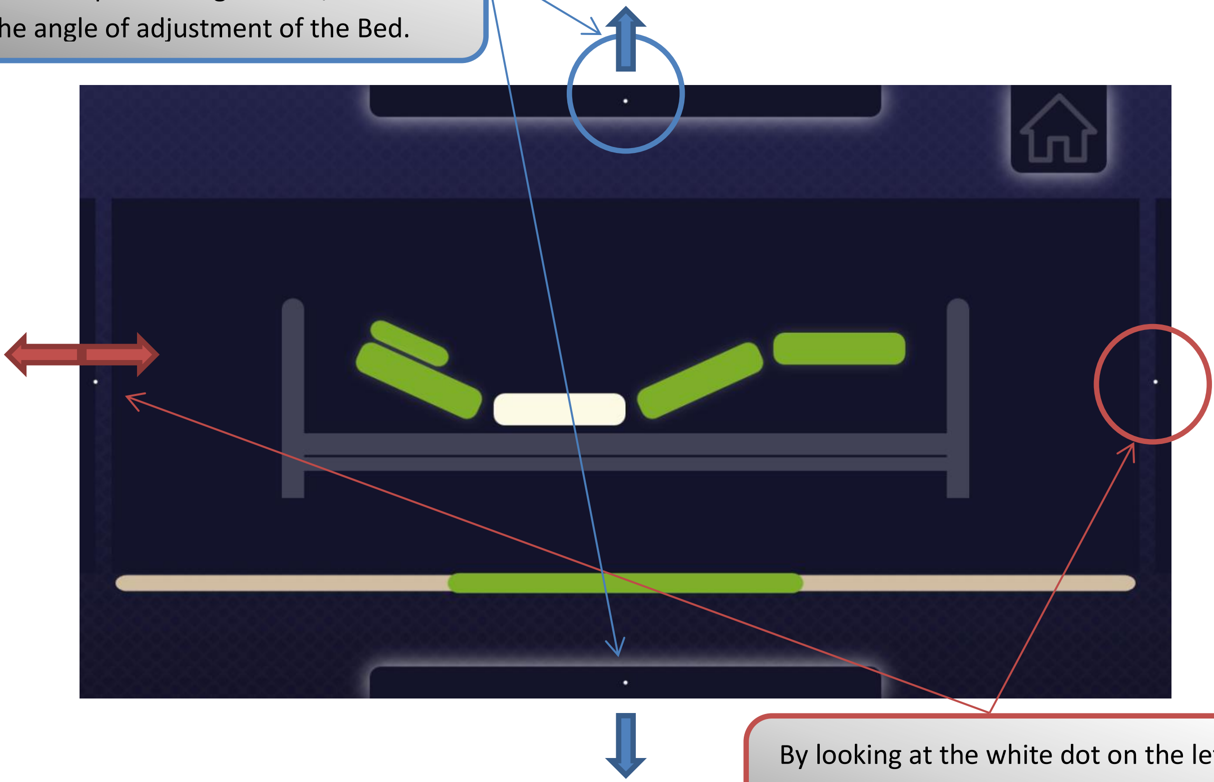

By looking at the white dot on the left or right, we scroll through the segments to the left/right.

Interrupt the positioning by closing your eyes or interrupting the view of theconfirmation element! 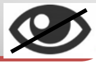

Segment selection

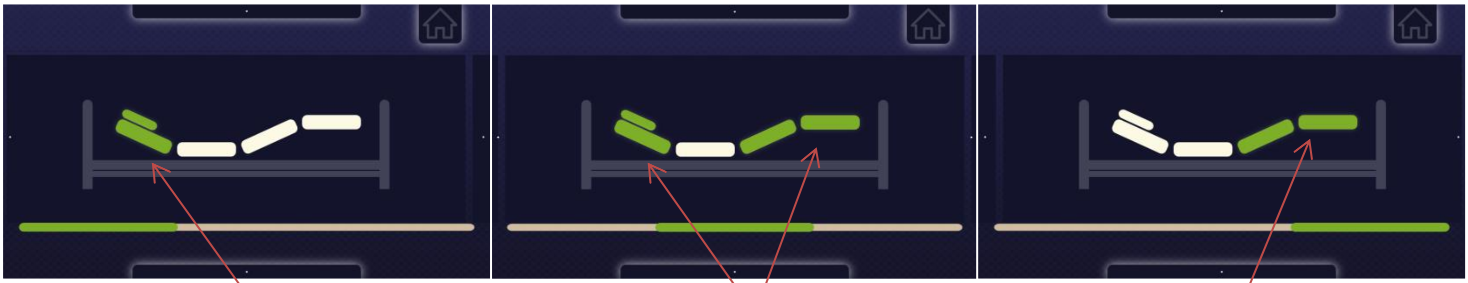

Headrest control

Headrest and leg rest control

Leg rest control

It is possible to choose from three offered positioning

Basic position (CPR)

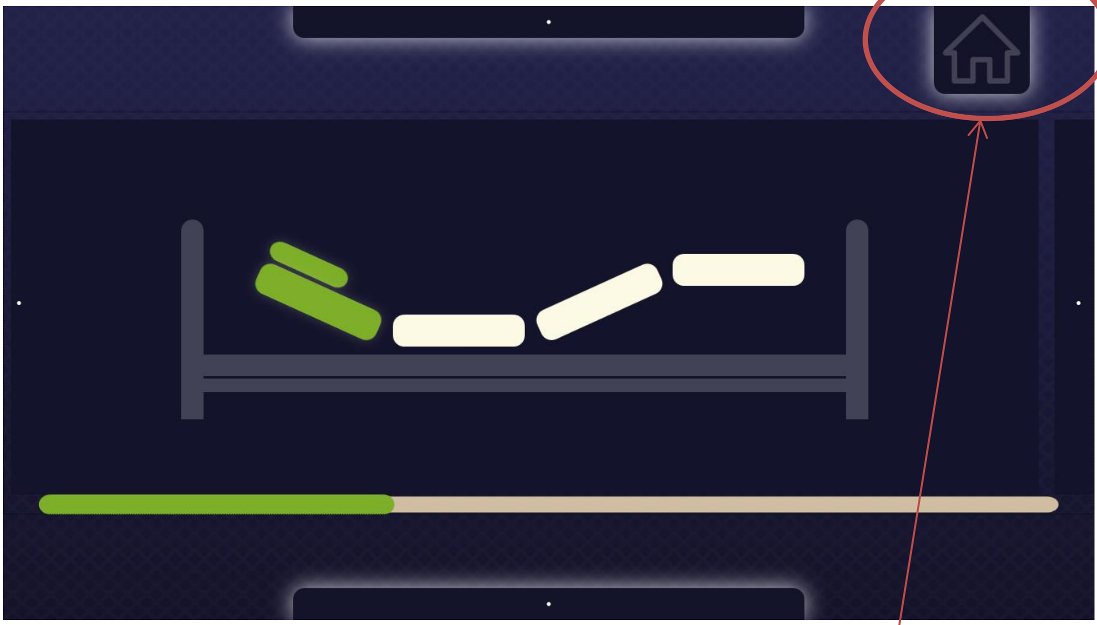

To bring the bed into the basic – straight low position, we move our eyes to the house in the upper right corner.

Confirm with the confirmationem element. 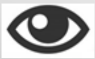

Supplement: Supplementary file 1 — Additional file 1. Instructions for contactless control of the adjustable bed application. Before starting the individual task, the volunteer was familiarized with the basic instructions for operating the positioning bed using a picture manual. [file 12984_2023_1193_MOESM1_ESM.pdf]
