# Supplementary material for: Eye-tracking control of an adjustable electric bed: construction and validation by immobile patients with multiple sclerosis
Source: J Neuroeng Rehabil. 2023 Jun 9;20:75. doi: 10.1186/s12984-023-01193-w (PMC10251586; doi:10.1186/s12984-023-01193-w)
Supplement: Supplementary file 3 — Additional file 3. List of questionnaires patients and controls. A sample of translated questionnaire is on the first page of the file, followed by scanned anonymized questionnaires. [file 12984_2023_1193_MOESM3_ESM.pdf]

Information, questionnaire and experiment protocol for

Contactless control of positioning bed (Sample page)

investigator: Martin Kopeček, MEng, kopecema@lfhk.cuni.cz

Fill in the questionnaire as soon as possible after the test.

Questions for the instructor

Day of experiment performed:

Name of instructor:

Patient name: sex: age:

Application log number:

Type of restriction: Multiple sclerosis, Paraparesis, Paraplegia, Quadriplegia, Pentaplegia, Muscular dystrophy, ALS

Other:

Momentum description:

Glasses/Contact Lenses: Yes / No

Experiment log:

1. Sensor calibration ☐
2. Explaining the principle and allowing you to gain experience with control, at least 10 minutes ☐
3. Putting applications in initial conditions – bed at zero position ☐

Time at start of testing: ....

4. **Test 1** ☐
   a) initiate eye contact with the application
   b) **raise** the leg rest (for 2-3 s)
   c) **raise** the headrest (for 2-3 s)
   d) **lower** the leg rest (for 2-3 s)
   e) **lower** the headrest (for 2-3 s)
   f) break eye contact with the app

7. Test **2** – repetition of **Test 1** ☐
8. Test **3** – repetition of **Test 1** ☐

Time at end of testing:............ ....

Instructor's notes on the experiment:

Questions for the patient

Have you gained certainty of control? *yes / rather yes/ don't know / rather no / no*

How satisfied are you with the operation of the application?

Activating control very easy / easy / ordinary / difficult / *very difficult*

Information on bed setup very easy / easy / ordinary / difficult/ *very difficult*

Position selection very easy / easy / ordinary / difficult / *very difficult*

Clarity of control very clear / clear / common / confusing / *very confusing*

Would you prefer to control in a different way? *yes / rather yes/ don't know / rather no / no*

If so, which ones (e.g. speech)? ....

Do you have any insight into the test or control?

How would you rate yourand current pain intensity on the above image scale?

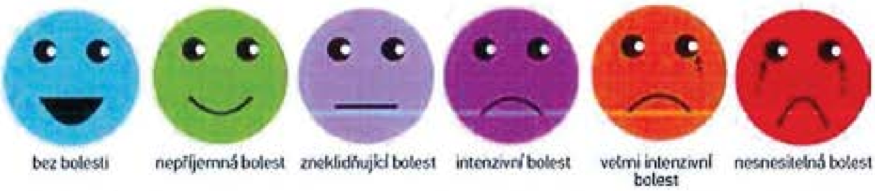

MALÁ NEBO ŽÁDNÁ BOLEST<br>Jste schopni se bolesti přizpůsobit	Velmi mírná bolest	☐	Velmi lehká, sotva znatelná bolest
	Nepříjemná bolest	☐	Menší bolest, lze ji přirovnat k lehkému štípání
	Přijatelná bolest	☐	Velmi nápadná bolest podobající se píchnutí injekce
MÍRNÁ BOLEST<br>Neslučitelná s mnoha aktivitami	Zneklidňující bolest	☐	Silná, hluboká bolest
	Velmi zneklidňující bolest	☐	Silná, hluboká, pronikavá bolest, např. vyvrknutý kotník
	Intenzivní bolest	☐	Ještě silnější bolest srovnatelná s bodnutím několika včelích žihadel
TĚŽKÁ BOLEST<br>Nejste schopni fungovat	Velmi intenzivní bolest	☐	Bolest srovnatelná s průměrnou migrénou
	Naprosto hrozná bolest	☐	Bolest srovnatelná s porodem nebo opravdu silnou migrénou
	Nesnesitelná bolest	☐	Bolest nelze ignorovat, vyžaduje léky proti bolesti, nutné navštívit lékaře

How would you assess your usual level of visual attention or stamina when watching a regular feature film?

I watch without problems / I watch with breaks / I can't watch / I don't care

Thank you for participating in the experiment and filling out the questionnaire...

19 VIDEO

P1

Informace, dotazník a protokol experimentu pro
Bezkontaktní ovládání polohovacího lůžka

řešitel: Ing. Martin Kopeček, MEng, kopecema@lfhk.cuni.cz

Dotazník vyplňte co možná nejdříve po provedení testu.

Otázky pro instruktora

Experiment proveden dne: [illegible].3.2021

Jméno instruktora: MK

Jméno pacienta: Pohlaví: M Věk: 67

Číslo logu aplikace: [illegible]

Typ omezení: Paraparéza, Paraplegie, Kvadruplegie, Pentaplegie, Svalové dystrofie, ALS,

Jiné: RS

Popis hybnosti: HYBNOST POUZE HLAVA, NNÍ NUTNOST ASISTENCE, ZVEDÁK

Brýle / kontaktní čočky: PŘI EXPERIMENTU NEBYLY ano / (ne)

Protokol experimentu:

1. Kalibrace senzoru ☑
2. Vysvětlení principu a umožnění získat zkušenost s ovládáním, alespoň 10 minut ☑
3. Uvedení aplikaci do počátečních podmínek – lůžko v bodě nula ☑

Čas při započetí testování: 11:20

4. **Test 1** ☑
   a) zahájit oční kontakt s aplikací
   b) **zvednout** podpěru nohou (po dobu 2-3 s)
   c) **zvednout** podpěru hlavy (po dobu 2-3 s)
   d) **snížit** podpěru nohou (po dobu 2-3 s)
   e) **snížit** podpěru hlavy (po dobu 2-3 s)
   f) přerušit oční kontakt s aplikací
7. **Test 2** – opakování **Testu 1** ☑
8. **Test 3** – opakování **Testu 1** ☑

Čas při ukončení testování: 11:35

Poznámky instruktora k experimentu:

KALIBRACE 2X -> ZLEPŠENÍ

MÁ EL. VOZÍK NA BRADU VE VÝROBĚ

P1

Otázky pro pacienta

Získal/a jste jistotu v ovládání? **ano** / spíše ano/ nevím / spíše ne / ne

Jak jste spokojen/a s ovládáním aplikace?

- aktivace ovládání — *velmi snadná / **snadná** / běžná / obtížná / velmi obtížná*
- způsob volby polohy — *velmi snadný / **snadný** / běžný / obtížný / velmi obtížný*
- informace o nastavení lůžka — ***velmi snadné** / snadné / běžné / obtížné / velmi obtížné*
- přehlednost ovládání — ***velmi přehledné** / přehledné/ běžné / nepřehledné/ velmi nepřehledné*

Upřednostnil/a byste ovládání jiným způsobem? *ano / spíše ano/ **nevím** / spíše ne / ne*

Pokud ano jakým (např. řeč)? ...... BUDE OVLÁDÁNÍ ŘEČÍ TV - TELEFON

Máte nějaký poznatek k testu nebo ovládání?

JAZYK OK, VELIKOST OBRAZOVKY „V POHODĚ"

Jak byste hodnotil/a Vaši aktuální intenzitu bolesti na uvedené obrazové škále?

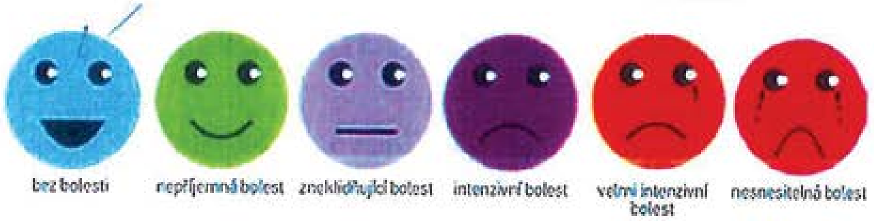

**MALÁ NEBO ŽÁDNÁ BOLEST** Jste schopni se bolesti přizpůsobit	Velmi mírná bolest	☐	Velmi lehká, sotva znatelná bolest
	Nepříjemná bolest	☐	Menší bolest, lze ji přirovnat k lehkému štípání
	Přijatelná bolest	☐	Velmi nápadná bolest podobající se píchnutí injekce
**MÍRNÁ BOLEST** Neslučitelná s mnoha aktivitami	Zneklidňující bolest	☐	Silná, hluboká bolest
	Velmi zneklidňující bolest	☐	Silná, hluboká, pronikavá bolest, např. vyvrknutý kotník
	Intenzivní bolest	☐	Ještě silnější bolest srovnatelná s bodnutím několika včelích žihadel
**TĚŽKÁ BOLEST** Nejste schopni fungovat	Velmi intenzivní bolest	☐	Bolest srovnatelná s průměrnou migrénou
	Naprosto hrozná bolest	☐	Bolest srovnatelná s porodem nebo opravdu silnou migrénou
	Nesnesitelná bolest	☐	Bolest nelze ignorovat, vyžaduje léky proti bolesti, nutné navštívit lékaře

Jak byste posoudil/a Vaši obvyklou míru zrakové pozornosti nebo výdrže při sledování běžného celovečerního filmu?

***sleduji bez problémů** / sleduji s přestávkami / nemůžu sledovat / nezajímá mne to*

Děkujeme Vám za účast v experimentu a při vyplnění dotazníku...

P2

Informace, dotazník a protokol experimentu pro
Bezkontaktní ovládání polohovacího lůžka

řešitel: Ing. Martin Kopeček, MEng, kopecema@lfhk.cuni.cz

Dotazník vyplňte co možná nejdříve po provedení testu.

Otázky pro instruktora

Experiment proveden dne: 4.5.2021

Jméno instruktora: MK

Jméno pacienta: [illegible] Pohlaví: M Věk: 73

Číslo logu aplikace: [illegible] 1,2,3

Typ omezení: Paraparéza, Paraplegie, Kvadruplegie, Pentaplegie, Svalové dystrofie, ALS,

Jiné: RS, .

Popis hybnosti: DOLNÍ KONČETINY NE, RUCE HORŠÍ, STISK A ÚCHOP TLAČÍTKA

Brýle / kontaktní čočky: (ano) / ne

Protokol experimentu:

1. Kalibrace senzoru ☑
2. Vysvětlení principu a umožnění získat zkušenost s ovládáním, alespoň 10 minut ☑
3. Uvedení aplikaci do počátečních podmínek – lůžko v bodě nula ☑

Čas při započetí testování: 12:30

4. **Test 1** ☑
   a) zahájit oční kontakt s aplikací
   b) **zvednout** podpěru nohou (po dobu 2-3 s)
   c) **zvednout** podpěru hlavy (po dobu 2-3 s)
   d) **snížit** podpěru nohou (po dobu 2-3 s)
   e) **snížit** podpěru hlavy (po dobu 2-3 s)
   f) přerušit oční kontakt s aplikací

7. **Test 2** – opakování **Testu 1** ☑
8. **Test 3** – opakování **Testu 1** ☑

Čas při ukončení testování: 12:50

Poznámky instruktora k experimentu:

P2

Otázky pro pacienta

Získal/a jste jistotu v ovládání? ano / spíše ano / nevím / spíše ne / ne

Jak jste spokojen/a s ovládáním aplikace?

- aktivace ovládání — *velmi snadná / snadná / běžná / obtížná / velmi obtížná*
- způsob volby polohy — *velmi snadný / snadný / běžný / obtížný / velmi obtížný*
- informace o nastavení lůžka — *velmi snadné / snadné / běžné / obtížné / velmi obtížné*
- přehlednost ovládání — *velmi přehledné / přehledné / běžné / nepřehledné / velmi nepřehledné*

Upřednostnil/a byste ovládání jiným způsobem? *ano / spíše ano / nevím / spíše ne / ne*

Pokud ano jakým (např. řeč)? ....

Máte nějaký poznatek k testu nebo ovládání?

DOBRÁ OSTROST → BAREVNOST OK - ZELENÁ OK
POZADÍ TROCHU SVĚTLEJŠÍ

Jak byste hodnotil/a Vaši aktuální intenzitu bolesti na uvedené obrazové škále?

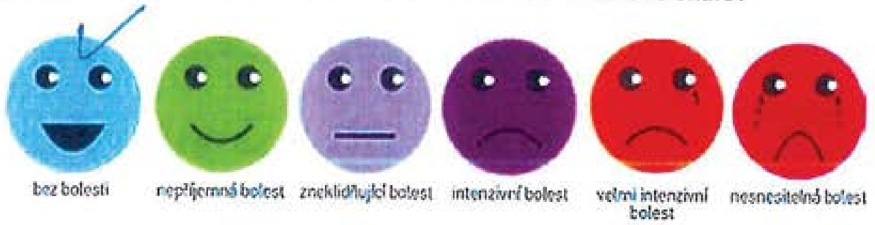

**MALÁ NEBO ŽÁDNÁ BOLEST** Jste schopni se bolesti přizpůsobit	Velmi mírná bolest	☐	Velmi lehká, sotva znatelná bolest
	Nepříjemná bolest	☐	Menší bolest, lze ji přirovnat k lehkému štípání
	Přijatelná bolest	☐	Velmi nápadná bolest podobající se píchnutí injekce
**MÍRNÁ BOLEST** Neslučitelná s mnoha aktivitami	Zneklidňující bolest	☐	Silná, hluboká bolest
	Velmi zneklidňující bolest	☐	Silná, hluboká, pronikavá bolest, např. vyvrknutý kotník
	Intenzivní bolest	☐	Ještě silnější bolest srovnatelná s bodnutím několika včelích žihadel
**TĚŽKÁ BOLEST** Nejste schopni fungovat	Velmi intenzivní bolest	☐	Bolest srovnatelná s průměrnou migrénou
	Naprosto hrozná bolest	☐	Bolest srovnatelná s porodem nebo opravdu silnou migrénou
	Nesnesitelná bolest	☐	Bolest nelze ignorovat, vyžaduje léky proti bolesti, nutné navštívit lékaře

Jak byste posoudil/a Vaši obvyklou míru zrakové pozornosti nebo výdrže při sledování běžného celovečerního filmu?

sleduji bez problémů / sleduji s přestávkami / nemůžu sledovat / nezajímá mne to

Děkujeme Vám za účast v experimentu a při vyplnění dotazníku...

P3

Informace, dotazník a protokol experimentu pro

Bezkontaktní ovládání polohovacího lůžka

řešitel: Ing. Martin Kopeček, MEng, kopecema@lfhk.cuni.cz

Dotazník vyplňte co možná nejdříve po provedení testu.

Otázky pro instruktora

Experiment proveden dne: 10.3.2021

Jméno instruktora: MK

Jméno pacienta: Pohlaví: Ž Věk: 67

Číslo logu aplikace: 1,2,3

Typ omezení: Paraparéza, Paraplegie, Kvadruplegie, Pentaplegie, Svalové dystrofie, ALS,

Jiné: RS

Popis hybnosti: RUCE - LZE ČÁSTEČNĚ OVLÁDAT OVLADAČ EL. INV. VOZÍKU

Brýle / kontaktní čočky: ano (ne)

Protokol experimentu:

1. Kalibrace senzoru ☑
2. Vysvětlení principu a umožnění získat zkušenost s ovládáním, alespoň 10 minut ☑
3. Uvedení aplikaci do počátečních podmínek – lůžko v bodě nula ☑

Čas při započetí testování: 7:45

4. Test 1 ☐
   a) zahájit oční kontakt s aplikací
   b) **zvednout** podpěru nohou (po dobu 2-3 s)
   c) **zvednout** podpěru hlavy (po dobu 2-3 s)
   d) **snížit** podpěru nohou (po dobu 2-3 s)
   e) **snížit** podpěru hlavy (po dobu 2-3 s)
   f) přerušit oční kontakt s aplikací

7. **Test 2** – opakování **Testu 1** ☑
8. **Test 3** – opakování **Testu 1** ☑

Čas při ukončení testování: 8:15

Poznámky instruktora k experimentu: LÉPE NASTAVIT SENZOR

P3

Otázky pro pacienta

Získal/a jste jistotu v ovládání? ano / spíše ano / nevím / spíše ne / ne

Jak jste spokojen/a s ovládáním aplikace?

aktivace ovládání velmi snadná / snadná / běžná / obtížná / velmi obtížná

způsob volby polohy velmi snadný / snadný / běžný / obtížný / velmi obtížný

informace o nastavení lůžka velmi snadné / snadné / běžné / obtížné / velmi obtížné

přehlednost ovládání velmi přehledné / přehledné / běžné / nepřehledné / velmi nepřehledné

Upřednostnil/a byste ovládání jiným způsobem? ano / spíše ano / nevím / spíše ne / ne

Pokud ano jakým (např. řeč)? .... POMOCÍ RUKOU ....

Máte nějaký poznatek k testu nebo ovládání? PŘI TEPLOTĚ NEOVLÁDÁ STISK

Jak byste hodnotil/a Vaši aktuální intenzitu bolesti na uvedené obrazové škále?

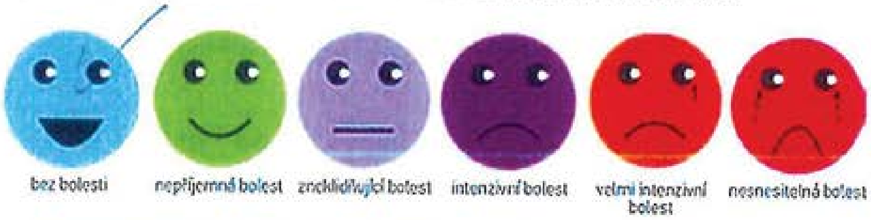

MALÁ NEBO ŽÁDNÁ BOLEST Jste schopni se bolesti přizpůsobit	Velmi mírná bolest	☐	Velmi lehká, sotva znatelná bolest
	Nepříjemná bolest	☐	Menší bolest, lze ji přirovnat k lehkému štípání
	Přijatelná bolest	☐	Velmi nápadná bolest podobající se píchnutí injekce
MÍRNÁ BOLEST Neslučitelná s mnoha aktivitami	Zneklidňující bolest	☐	Silná, hluboká bolest
	Velmi zneklidňující bolest	☐	Silná, hluboká, pronikavá bolest, např. vyvrknutý kotník
	Intenzivní bolest	☐	Ještě silnější bolest srovnatelná s bodnutím několika včelích žihadel
TĚŽKÁ BOLEST Nejste schopni fungovat	Velmi intenzivní bolest	☐	Bolest srovnatelná s průměrnou migrénou
	Naprosto hrozná bolest	☐	Bolest srovnatelná s porodem nebo opravdu silnou migrénou
	Nesnesitelná bolest	☐	Bolest nelze ignorovat, vyžaduje léky proti bolesti, nutné navštívit lékaře

Jak byste posoudil/a Vaši obvyklou míru zrakové pozornosti nebo výdrže při sledování běžného celovečerního filmu?

sleduji bez problémů / sleduji s přestávkami / nemůžu sledovat / nezajímá mne to

Děkujeme Vám za účast v experimentu a při vyplnění dotazníku...

#P P4

Informace, dotazník a protokol experimentu pro
Bezkontaktní ovládání polohovacího lůžka

řešitel: Ing. Martin Kopeček, MEng, kopecema@lfhk.cuni.cz

Dotazník vyplňte co možná nejdříve po provedení testu.

Otázky pro instruktora

Experiment proveden dne: 10. 5. 2021

Jméno instruktora: MK

Jméno pacienta: Pohlaví: M Věk: 57

Číslo logu aplikace:

Typ omezení: Paraparéza, Paraplegie, Kvadruplegie, Pentaplegie, Svalové dystrofie, ALS,

Jiné: RS, OSTEOPOROZA

Popis hybnosti: EL. VOZÍK + FR. HOLE, PŘI SPATNÉ KONSTRUKCI NELZE HOL RUKAMA

Brýle / kontaktní čočky: BRÝLE NA DÁLKU, ROZOSTŘOVÁNÍ OČÍ ano / ne (ne circled)

Protokol experimentu:

1. Kalibrace senzoru ☑
2. Vysvětlení principu a umožnění získat zkušenost s ovládáním, alespoň 10 minut ☑
3. Uvedení aplikaci do počátečních podmínek – lůžko v bodě nula ☑

Čas při započetí testování: 9^00

4. Test 1 ☑
   a) zahájit oční kontakt s aplikací
   b) **zvednout** podpěru nohou (po dobu 2-3 s)
   c) **zvednout** podpěru hlavy (po dobu 2-3 s)
   d) **snížit** podpěru nohou (po dobu 2-3 s)
   e) **snížit** podpěru hlavy (po dobu 2-3 s)
   f) přerušit oční kontakt s aplikací
7. **Test 2** – opakování **Testu 1** ☑
8. **Test 3** – opakování **Testu 1** ☑

Čas při ukončení testování: 9^19

Poznámky instruktora k experimentu:

P4

Otázky pro pacienta

Získal/a jste jistotu v ovládání? *ano / spíše ano / nevím / spíše ne / ne*

Jak jste spokojen/a s ovládáním aplikace?

aktivace ovládání *velmi snadná / snadná / běžná / obtížná / velmi obtížná*

způsob volby polohy *velmi snadný / snadný / běžný / obtížný / velmi obtížný*

informace o nastavení lůžka *velmi snadné / snadné / běžné / obtížné / velmi obtížné*

přehlednost ovládání *velmi přehledné / přehledné / běžné / nepřehledné / velmi nepřehledné*

Upřednostnil/a byste ovládání jiným způsobem? *ano / spíše ano / nevím / spíše ne / ne*

Pokud ano jakým (např. řeč)? POMOCÍ [illegible]

Máte nějaký poznatek k testu nebo ovládání?

Jak byste hodnotil/a Vaši aktuální intenzitu bolesti na uvedené obrazové škále?

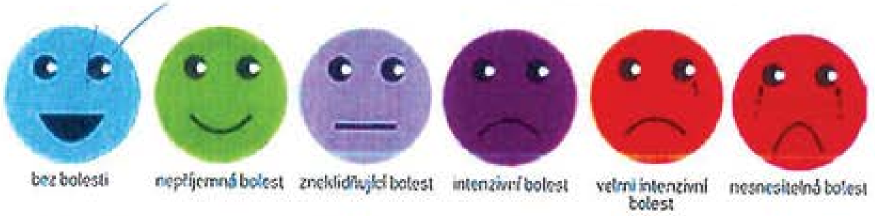

**MALÁ NEBO ŽÁDNÁ BOLEST** Jste schopni se bolesti přizpůsobit	Velmi mírná bolest	☐	Velmi lehká, sotva znatelná bolest
	Nepříjemná bolest	☐	Menší bolest, lze ji přirovnat k lehkému štípání
	Přijatelná bolest	☐	Velmi nápadná bolest podobající se píchnutí injekce
**MÍRNÁ BOLEST** Neslučitelná s mnoha aktivitami	Zneklidňující bolest	☐	Silná, hluboká bolest
	Velmi zneklidňující bolest	☐	Silná, hluboká, pronikavá bolest, např. vyvrknutý kotník
	Intenzivní bolest	☐	Ještě silnější bolest srovnatelná s bodnutím několika včelích žihadel
**TĚŽKÁ BOLEST** Nejste schopni fungovat	Velmi intenzivní bolest	☐	Bolest srovnatelná s průměrnou migrénou
	Naprosto hrozná bolest	☐	Bolest srovnatelná s porodem nebo opravdu silnou migrénou
	Nesnesitelná bolest	☐	Bolest nelze ignorovat, vyžaduje léky proti bolesti, nutné navštívit lékaře

Jak byste posoudil/a Vaši obvyklou míru zrakové pozornosti nebo výdrže při sledování běžného celovečerního filmu?

sleduji bez problémů / sleduji s přestávkami / nemůžu sledovat / nezajímá mne to

S [illegible] PAUZY

Děkujeme Vám za účast v experimentu a při vyplnění dotazníku...

ðŸ

P5

Informace, dotazník a protokol experimentu pro
Bezkontaktní ovládání polohovacího lůžka

řešitel: Ing. Martin Kopeček, MEng, kopecema@lfhk.cuni.cz

Dotazník vyplňte co možná nejdříve po provedení testu.

Otázky pro instruktora

Experiment proveden dne: 10. 5. 2021

Jméno instruktora: MK

Jméno pacienta: Pohlaví: Ž Věk: 54

Číslo logu aplikace: 1, 2, 3

Typ omezení: Paraparéza, Paraplegie, Kvadruplegie, Pentaplegie, Svalové dystrofie, ALS,

Jiné: RS

Popis hybnosti: LEVÁ RUKA PLEGICKÁ, PRAVÁ RUKA HYBNOST DOBRÁ, MECH. VOZÍK

Brýle / kontaktní čočky: JINAK BRÝLE 3D KRÁTKOZRAKOST ano / ne

Protokol experimentu:

1. Kalibrace senzoru ☑
2. Vysvětlení principu a umožnění získat zkušenost s ovládáním, alespoň 10 minut ☑
3. Uvedení aplikaci do počátečních podmínek – lůžko v bodě nula ☑

Čas při započetí testování: 13[00]

4. **Test 1** ☑
   a) zahájit oční kontakt s aplikací
   b) **zvednout** podpěru nohou (po dobu 2-3 s)
   c) **zvednout** podpěru hlavy (po dobu 2-3 s)
   d) **snížit** podpěru nohou (po dobu 2-3 s)
   e) **snížit** podpěru hlavy (po dobu 2-3 s)
   f) přerušit oční kontakt s aplikací
7. **Test 2** – opakování **Testu 1** ☑
8. **Test 3** – opakování **Testu 1** ☑

Čas při ukončení testování: 13[25]

Poznámky instruktora k experimentu:

EL. VOZÍK OBJEDNANÝ
PROBLÉM S DOLNÍ LIŠTOU
PROBLÉM UDRŽENÍ KONCENTRACE

P5

Otázky pro pacienta

Získal/a jste jistotu v ovládání? *ano / spíše ano / nevím / spíše ne / ne*

Jak jste spokojen/a s ovládáním aplikace?

- aktivace ovládání — *velmi snadná / snadná / běžná / obtížná / velmi obtížná*
- způsob volby polohy — *velmi snadný / snadný / běžný / obtížný / velmi obtížný*
- informace o nastavení lůžka — *velmi snadné / snadné / běžné / obtížné / velmi obtížné*
- přehlednost ovládání — *velmi přehledné / přehledné / běžné / nepřehledné / velmi nepřehledné*

Upřednostnil/a byste ovládání jiným způsobem? *ano / spíše ano / nevím / spíše ne / ne*

Pokud ano jakým (např. řeč)? RUČNÍ, S JAKÝM NEVÍM [illegible]

Máte nějaký poznatek k testu nebo ovládání?

[illegible] JEN TO JE

Jak byste hodnotil/a Vaši aktuální intenzitu bolesti na uvedené obrazové škále?

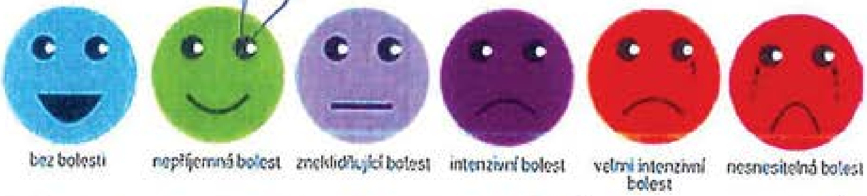

MALÁ NEBO ŽÁDNÁ BOLEST<br>Jste schopni se bolesti přizpůsobit	Velmi mírná bolest	☑	Velmi lehká, sotva znatelná bolest
	Nepříjemná bolest	☐	Menší bolest, lze ji přirovnat k lehkému štípání
	Přijatelná bolest	☐	Velmi nápadná bolest podobající se píchnutí injekce
MÍRNÁ BOLEST<br>Neslučitelná s mnoha aktivitami	Zneklidňující bolest	☐	Silná, hluboká bolest
	Velmi zneklidňující bolest	☐	Silná, hluboká, pronikavá bolest, např. vyvrknutý kotník
	Intenzivní bolest	☐	Ještě silnější bolest srovnatelná s bodnutím několika včelích žihadel
TĚŽKÁ BOLEST<br>Nejste schopni fungovat	Velmi intenzivní bolest	☐	Bolest srovnatelná s průměrnou migrénou
	Naprosto hrozná bolest	☐	Bolest srovnatelná s porodem nebo opravdu silnou migrénou
	Nesnesitelná bolest	☐	Bolest nelze ignorovat, vyžaduje léky proti bolesti, nutné navštívit lékaře

Jak byste posoudil/a Vaši obvyklou míru zrakové pozornosti nebo výdrže při sledování běžného celovečerního filmu?

sleduji bez problémů / sleduji s přestávkami / nemůžu sledovat / nezajímá mne to

ÚNAVA OČÍ

Děkujeme Vám za účast v experimentu a při vyplnění dotazníku...

2P

P6

Informace, dotazník a protokol experimentu pro
Bezkontaktní ovládání polohovacího lůžka

řešitel: Ing. Martin Kopeček, MEng, kopecema@lfhk.cuni.cz

Dotazník vyplňte co možná nejdříve po provedení testu.

Otázky pro instruktora

Experiment proveden dne: 10.5.2021

Jméno instruktora: MK

Jméno pacienta: Pohlaví: Ž Věk: 70

Číslo logu aplikace: 7,2,0

Typ omezení: Paraparéza, Paraplegie, Kvadruplegie, Pentaplegie, Svalové dystrofie, ALS,

Jiné: RS

Popis hybnosti: RUČZOK, LEVÁ HORŠÍ, ELEKT. + MECH. VOZÍK

Brýle / kontaktní čočky: ~~BIFOKÁLNÍ~~ (ano) / ne

Protokol experimentu:

1. Kalibrace senzoru ☑
2. Vysvětlení principu a umožnění získat zkušenost s ovládáním, alespoň 10 minut ☑
3. Uvedení aplikaci do počátečních podmínek – lůžko v bodě nula ☑

Čas při započetí testování: 14⁰⁰

4. **Test 1** ☑
   a) zahájit oční kontakt s aplikací
   b) **zvednout** podpěru nohou (po dobu 2-3 s)
   c) **zvednout** podpěru hlavy (po dobu 2-3 s)
   d) **snížit** podpěru nohou (po dobu 2-3 s)
   e) **snížit** podpěru hlavy (po dobu 2-3 s)
   f) přerušit oční kontakt s aplikací
7. **Test 2** – opakování **Testu 1** ☑
8. **Test 3** – opakování **Testu 1** ☑

Čas při ukončení testování: 14²⁵

Poznámky instruktora k experimentu:

P6

Otázky pro pacienta

Získal/a jste jistotu v ovládání? ano / **spíše ano** / nevím / spíše ne / ne

Jak jste spokojen/a s ovládáním aplikace?

- aktivace ovládání — *velmi snadná / **snadná** / běžná / obtížná / velmi obtížná*
- způsob volby polohy — *velmi snadný / **snadný** / běžný / obtížný / velmi obtížný*
- informace o nastavení lůžka — *velmi snadné / **snadné** / běžné / obtížné / velmi obtížné*
- přehlednost ovládání — *velmi přehledné / **přehledné** / běžné / nepřehledné / velmi nepřehledné*

Upřednostnil/a byste ovládání jiným způsobem? *ano / **spíše ano** / nevím / spíše ne / ne*

Pokud ano jakým (např. řeč)? .... POMOCÍ RUKOU ....

Máte nějaký poznatek k testu nebo ovládání?

....

....

Jak byste hodnotil/a Vaši aktuální intenzitu bolesti na uvedené obrazové škále?

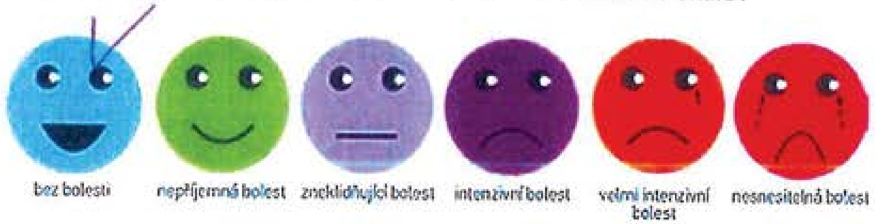

**MALÁ NEBO ŽÁDNÁ BOLEST** Jste schopni se bolesti přizpůsobit	Velmi mírná bolest	☐	Velmi lehká, sotva znatelná bolest
	Nepříjemná bolest	☐	Menší bolest, lze ji přirovnat k lehkému štípání
	Přijatelná bolest	☐	Velmi nápadná bolest podobající se píchnutí injekce
**MÍRNÁ BOLEST** Neslučitelná s mnoha aktivitami	Zneklidňující bolest	☐	Silná, hluboká bolest
	Velmi zneklidňující bolest	☐	Silná, hluboká, pronikavá bolest, např. vyvrknutý kotník
	Intenzivní bolest	☐	Ještě silnější bolest srovnatelná s bodnutím několika včelích žihadel
**TĚŽKÁ BOLEST** Nejste schopni fungovat	Velmi intenzivní bolest	☐	Bolest srovnatelná s průměrnou migrénou
	Naprosto hrozná bolest	☐	Bolest srovnatelná s porodem nebo opravdu silnou migrénou
	Nesnesitelná bolest	☐	Bolest nelze ignorovat, vyžaduje léky proti bolesti, nutné navštívit lékaře

Jak byste posoudil/a Vaši obvyklou míru zrakové pozornosti nebo výdrže při sledování běžného celovečerního filmu?

__sleduji bez problémů__ / sleduji s přestávkami / nemůžu sledovat / nezajímá mne to

Děkujeme Vám za účast v experimentu a při vyplnění dotazníku...

5.9

P7

Informace, dotazník a protokol experimentu pro
Bezkontaktní ovládání polohovacího lůžka

řešitel: Ing. Martin Kopeček, MEng, kopecema@lfhk.cuni.cz

Dotazník vyplňte co možná nejdříve po provedení testu.

Otázky pro instruktora

Experiment proveden dne: 11.5.2021

Jméno instruktora: MK

Jméno pacienta: Pohlaví: M Věk: 63

Číslo logu aplikace: 1,2,3

Typ omezení: Paraparéza, Paraplegie, Kvadruplegie, Pentaplegie, Svalové dystrofie, ALS,

Jiné: RS

Popis hybnosti: PLNÁ PARÉZA, POHYB ŽÁDNÝ

Brýle / kontaktní čočky: BRÝLE NA DIVADLO ano / ne (circled: ne)

Protokol experimentu:

1. Kalibrace senzoru ☑
2. Vysvětlení principu a umožnění získat zkušenost s ovládáním, alespoň 10 minut ☑
3. Uvedení aplikaci do počátečních podmínek – lůžko v bodě nula ☑

Čas při započetí testování: 7 30

4. **Test 1** ☑
   a) zahájit oční kontakt s aplikací
   b) **zvednout** podpěru nohou (po dobu 2-3 s)
   c) **zvednout** podpěru hlavy (po dobu 2-3 s)
   d) **snížit** podpěru nohou (po dobu 2-3 s)
   e) **snížit** podpěru hlavy (po dobu 2-3 s)
   f) přerušit oční kontakt s aplikací

7. **Test 2** – opakování **Testu 1** ☑
8. **Test 3** – opakování **Testu 1** ☑

Čas při ukončení testování: 7 40

Poznámky instruktora k experimentu:

Otázky pro pacienta

Získal/a jste jistotu v ovládání? *(ano) / spíše ano / nevím / spíše ne / ne*

Jak jste spokojen/a s ovládáním aplikace?

- aktivace ovládání — *(velmi snadná) / snadná / běžná / obtížná / velmi obtížná*
- způsob volby polohy — *velmi snadný / (snadný) / běžný / obtížný / velmi obtížný*
- informace o nastavení lůžka — *(velmi snadné) / snadné / běžné / obtížné / velmi obtížné*
- přehlednost ovládání — *velmi přehledné / (přehledné) / běžné / nepřehledné / velmi nepřehledné*

Upřednostnil/a byste ovládání jiným způsobem? *ano / (spíše ano) / nevím / spíše ne / ne*

Pokud ano jakým (např. řeč)? ŘEČ (MÁ PACIENT DOMA)

Máte nějaký poznatek k testu nebo ovládání?

MOUTH MOUSE – TV, ZÁSUVKY, [illegible] FILTRACE BAZÉN, ZVONEK, VENTILÁTOR – PŘIDAT DO OVLÁDÁNÍ ŘEČI

Jak byste hodnotil/a Vaši aktuální intenzitu bolesti na uvedené obrazové škále?

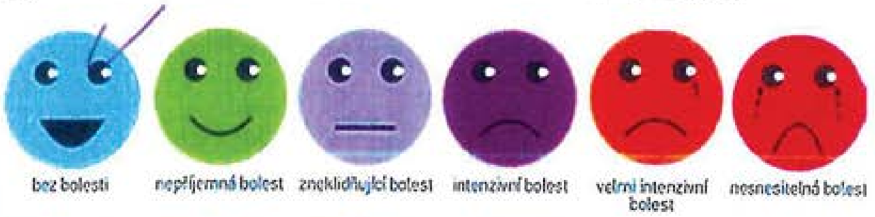

MALÁ NEBO ŽÁDNÁ BOLEST Jste schopni se bolesti přizpůsobit	Velmi mírná bolest	☐	Velmi lehká, sotva znatelná bolest
	Nepříjemná bolest	☐	Menší bolest, lze ji přirovnat k lehkému štípání
	Přijatelná bolest	☐	Velmi nápadná bolest podobající se píchnutí injekce
MÍRNÁ BOLEST Neslučitelná s mnoha aktivitami	Zneklidňující bolest	☐	Silná, hluboká bolest
	Velmi zneklidňující bolest	☐	Silná, hluboká, pronikavá bolest, např. vyvrknutý kotník
	Intenzivní bolest	☐	Ještě silnější bolest srovnatelná s bodnutím několika včelích žihadel
TĚŽKÁ BOLEST Nejste schopni fungovat	Velmi intenzivní bolest	☐	Bolest srovnatelná s průměrnou migrénou
	Naprosto hrozná bolest	☐	Bolest srovnatelná s porodem nebo opravdu silnou migrénou
	Nesnesitelná bolest	☐	Bolest nelze ignorovat, vyžaduje léky proti bolesti, nutné navštívit lékaře

Jak byste posoudil/a Vaši obvyklou míru zrakové pozornosti nebo výdrže při sledování běžného celovečerního filmu?

(sleduji bez problémů) / sleduji s přestávkami / nemůžu sledovat / nezajímá mne to

Děkujeme Vám za účast v experimentu a při vyplnění dotazníku...

8F

P8

Informace, dotazník a protokol experimentu pro
Bezkontaktní ovládání polohovacího lůžka

řešitel: Ing. Martin Kopeček, MEng, kopecema@lfhk.cuni.cz

Dotazník vyplňte co možná nejdříve po provedení testu.

Otázky pro instruktora

Experiment proveden dne: 11. 5. 2021

Jméno instruktora: MK

Jméno pacienta: [illegible] Pohlaví: Ž Věk: 64

Číslo logu aplikace: [illegible] ,2,3

Typ omezení: Paraparéza, Paraplegie, Kvadruplegie, Pentaplegie, Svalové dystrofie, ALS,

Jiné: RS

Popis hybnosti: PLNÁ PLEGIE, NEPOHYBLIVÉ RUCE A NOHY

Brýle / kontaktní čočky: JINAK NA ČTENÍ MUSÍ ano / ~~ne~~ (ne zakroužkováno)

Protokol experimentu:

1. Kalibrace senzoru ☑
2. Vysvětlení principu a umožnění získat zkušenost s ovládáním, alespoň 10 minut ☑
3. Uvedení aplikaci do počátečních podmínek – lůžko v bodě nula ☑

Čas při započetí testování: 9^{30}

4. **Test 1** ☑
   a) zahájit oční kontakt s aplikací
   b) **zvednout** podpěru nohou (po dobu 2-3 s)
   c) **zvednout** podpěru hlavy (po dobu 2-3 s)
   d) **snížit** podpěru nohou (po dobu 2-3 s)
   e) **snížit** podpěru hlavy (po dobu 2-3 s)
   f) přerušit oční kontakt s aplikací
7. **Test 2** – opakování **Testu 1** ☑
8. **Test 3** – opakování **Testu 1** ☑

Čas při ukončení testování: 9^{45}

Poznámky instruktora k experimentu:

VELMI DOBŘE SE ORIENTUJE, NEPOTŘEBUJE NAVÁDĚNÍ CO MÁ DĚLAT. VELMI RYCHLÉ UČENÍ.

P8

Otázky pro pacienta

Získal/a jste jistotu v ovládání? *ano / **spíše ano** / nevím / spíše ne / ne*

Jak jste spokojen/a s ovládáním aplikace?

- aktivace ovládání — *velmi snadná / **snadná** / běžná / obtížná / velmi obtížná*
- způsob volby polohy — *velmi snadný / snadný / **běžný** / obtížný / velmi obtížný*
- informace o nastavení lůžka — *velmi snadné / **snadné** / běžné / obtížné / velmi obtížné*
- přehlednost ovládání — ***velmi přehledné** / přehledné / běžné / nepřehledné / velmi nepřehledné*

Upřednostnil/a byste ovládání jiným způsobem? *ano / spíše ano / nevím / spíše ne / ne*

Pokud ano jakým (např. řeč)? .... ŘEČ NE → ŠPATNĚ MLUVÍ A FUNGUJE KVŮLI TOMU NEPŘESNĚ

Máte nějaký poznatek k testu nebo ovládání?

ZPOMALIT APP, REAGUJE RYCHLE ABY TO MLUVILO, TAK TO TOTÁŽ NECHCE, MÁ RÁDA KLID

Jak byste hodnotil/a Vaši aktuální intenzitu bolesti na uvedené obrazové škále?

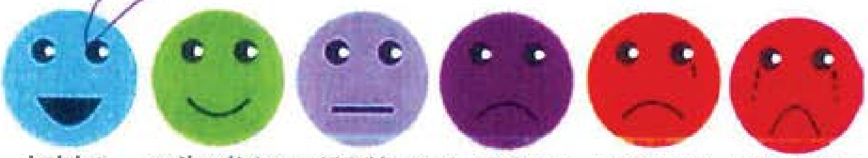

**MALÁ NEBO ŽÁDNÁ BOLEST** Jste schopni se bolesti přizpůsobit	Velmi mírná bolest	☐	Velmi lehká, sotva znatelná bolest
	Nepříjemná bolest	☐	Menší bolest, lze ji přirovnat k lehkému štípání
	Přijatelná bolest	☐	Velmi nápadná bolest podobající se píchnutí injekce
**MÍRNÁ BOLEST** Neslučitelná s mnoha aktivitami	Zneklidňující bolest	☐	Silná, hluboká bolest
	Velmi zneklidňující bolest	☐	Silná, hluboká, pronikavá bolest, např. vyvrknutý kotník
	Intenzivní bolest	☐	Ještě silnější bolest srovnatelná s bodnutím několika včelích žihadel
**TĚŽKÁ BOLEST** Nejste schopni fungovat	Velmi intenzivní bolest	☐	Bolest srovnatelná s průměrnou migrénou
	Naprosto hrozná bolest	☐	Bolest srovnatelná s porodem nebo opravdu silnou migrénou
	Nesnesitelná bolest	☐	Bolest nelze ignorovat, vyžaduje léky proti bolesti, nutné navštívit lékaře

Jak byste posoudil/a Vaši obvyklou míru zrakové pozornosti nebo výdrže při sledování běžného celovečerního filmu?

***sleduji bez problémů** / sleduji s přestávkami / nemůžu sledovat / nezajímá mne to*

Děkujeme Vám za účast v experimentu a při vyplnění dotazníku…

pf

P9

Informace, dotazník a protokol experimentu pro
Bezkontaktní ovládání polohovacího lůžka

řešitel: Ing. Martin Kopeček, MEng, kopecema@lfhk.cuni.cz

Dotazník vyplňte co možná nejdříve po provedení testu.

Otázky pro instruktora

Experiment proveden dne: 11.5.2021

Jméno instruktora: MK

Jméno pacienta: Pohlaví: M Věk: 64

Číslo logu aplikace: 1, 2, 3

Typ omezení: Paraparéza, Paraplegie, Kvadruplegie, Pentaplegie, Svalové dystrofie, ALS,

Jiné: RS

Popis hybnosti: PLNÁ PARÉZA, POHYB PAŽÍ A NOHOU ∅

Brýle / kontaktní čočky: ano / ~~ne~~ (ne zakroužkováno)

Protokol experimentu:

1. Kalibrace senzoru ☑
2. Vysvětlení principu a umožnění získat zkušenost s ovládáním, alespoň 10 minut ☑
3. Uvedení aplikaci do počátečních podmínek – lůžko v bodě nula ☑

   Čas při započetí testování: 10:10

4. **Test 1** ☑
   a) zahájit oční kontakt s aplikací
   b) **zvednout** podpěru nohou (po dobu 2-3 s)
   c) **zvednout** podpěru hlavy (po dobu 2-3 s)
   d) **snížit** podpěru nohou (po dobu 2-3 s)
   e) **snížit** podpěru hlavy (po dobu 2-3 s)
   f) přerušit oční kontakt s aplikací

7. **Test 2** – opakování **Testu 1** ☑
8. **Test 3** – opakování **Testu 1** ☑

   Čas při ukončení testování: 10:30

Poznámky instruktora k experimentu: PÁN NEMLUVÍ

P9

Otázky pro pacienta

Získal/a jste jistotu v ovládání? ano / spíše ano / nevím / spíše ne / ne

Jak jste spokojen/a s ovládáním aplikace?

- aktivace ovládání — velmi snadná / snadná / běžná / obtížná / velmi obtížná
- způsob volby polohy — velmi snadný / snadný / běžný / obtížný / velmi obtížný
- informace o nastavení lůžka — velmi snadné / snadné / běžné / obtížné / velmi obtížné
- přehlednost ovládání — velmi přehledné / přehledné / běžné / nepřehledné / velmi nepřehledné

Upřednostnil/a byste ovládání jiným způsobem? ano / spíše ano / nevím / spíše ne / ne

Pokud ano jakým (např. řeč)? ....

Máte nějaký poznatek k testu nebo ovládání? BYLO BY TO VELMI VHODNÉ

Jak byste hodnotil/a Vaši aktuální intenzitu bolesti na uvedené obrazové škále?

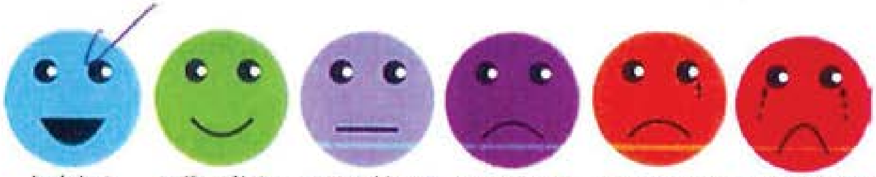

**MALÁ NEBO ŽÁDNÁ BOLEST** Jste schopni se bolesti přizpůsobit	Velmi mírná bolest	☐	Velmi lehká, sotva znatelná bolest
	Nepříjemná bolest	☐	Menší bolest, lze ji přirovnat k lehkému štípání
	Přijatelná bolest	☐	Velmi nápadná bolest podobající se píchnutí injekce
**MÍRNÁ BOLEST** Neslučitelná s mnoha aktivitami	Zneklidňující bolest	☐	Silná, hluboká bolest
	Velmi zneklidňující bolest	☐	Silná, hluboká, pronikavá bolest, např. vyvrknutý kotník
	Intenzivní bolest	☐	Ještě silnější bolest srovnatelná s bodnutím několika včelích žihadel
**TĚŽKÁ BOLEST** Nejste schopni fungovat	Velmi intenzivní bolest	☐	Bolest srovnatelná s průměrnou migrénou
	Naprosto hrozná bolest	☐	Bolest srovnatelná s porodem nebo opravdu silnou migrénou
	Nesnesitelná bolest	☐	Bolest nelze ignorovat, vyžaduje léky proti bolesti, nutné navštívit lékaře

Jak byste posoudil/a Vaši obvyklou míru zrakové pozornosti nebo výdrže při sledování běžného celovečerního filmu?

sleduji bez problémů / sleduji s přestávkami / nemůžu sledovat / nezajímá mne to

Děkujeme Vám za účast v experimentu a při vyplnění dotazníku...

P10

Informace, dotazník a protokol experimentu pro
Bezkontaktní ovládání polohovacího lůžka

řešitel: Ing. Martin Kopeček, MEng, kopecema@lfhk.cuni.cz

Dotazník vyplňte co možná nejdříve po provedení testu.

Otázky pro instruktora

Experiment proveden dne: 11.5.2021

Jméno instruktora: MK

Jméno pacienta: Pohlaví: Ž Věk: 65

Číslo logu aplikace: 1,2,3

Typ omezení: Paraparéza, Paraplegie, Kvadruplegie, Pentaplegie, Svalové dystrofie, ALS,

Jiné: RS

Popis hybnosti: PLNÁ PARÉZA, RUCE NOHY Ø

Brýle / kontaktní čočky: ŠPATNĚ NA DÁLKU ano / ne (ne circled)

Protokol experimentu:

1. Kalibrace senzoru ☑
2. Vysvětlení principu a umožnění získat zkušenost s ovládáním, alespoň 10 minut ☑
3. Uvedení aplikaci do počátečních podmínek – lůžko v bodě nula ☑

Čas při započetí testování: 11:15

4. **Test 1** ☑
   a) zahájit oční kontakt s aplikací
   b) **zvednout** podpěru nohou (po dobu 2-3 s)
   c) **zvednout** podpěru hlavy (po dobu 2-3 s)
   d) **snížit** podpěru nohou (po dobu 2-3 s)
   e) **snížit** podpěru hlavy (po dobu 2-3 s)
   f) přerušit oční kontakt s aplikací
7. **Test 2** – opakování **Testu 1** ☑
8. **Test 3** – opakování **Testu 1** ☑

Čas při ukončení testování: 11:40

Poznámky instruktora k experimentu:

P10

Otázky pro pacienta

Získal/a jste jistotu v ovládání? **ano** / spíše ano/ nevím / spíše ne / ne

Jak jste spokojen/a s ovládáním aplikace?

- aktivace ovládání — **velmi snadná** / snadná / běžná / obtížná / velmi obtížná
- způsob volby polohy — velmi snadný / **snadný** / běžný / obtížný / velmi obtížný
- informace o nastavení lůžka — velmi snadné / **snadné** / běžné / obtížné / velmi obtížné
- přehlednost ovládání — velmi přehledné / **přehledné** / běžné / nepřehledné/ velmi nepřehledné

Upřednostnil/a byste ovládání jiným způsobem? ano / spíše ano/ nevím / **spíše ne** / ne

Pokud ano jakým (např. řeč)? ZATÍM MŮŽE KLOUBEM S VYPĚTÍM SIL POMOCÍ KLOUBU POLOHOVAT

Máte nějaký poznatek k testu nebo ovládání? PŘI ZTRÁTĚ MOTORIKY Ø

OČ NATO DOBŘE VIDĚT, DÁL OD OČÍ BY BYL PROBLÉM.

Jak byste hodnotil/a Vaši aktuální intenzitu bolesti na uvedené obrazové škále?

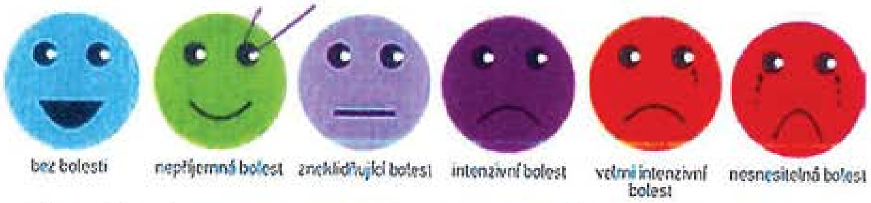

**MALÁ NEBO ŽÁDNÁ BOLEST** Jste schopni se bolesti přizpůsobit	Velmi mírná bolest	[x]	Velmi lehká, sotva znatelná bolest
	Nepříjemná bolest	[ ]	Menší bolest, lze ji přirovnat k lehkému štípání
	Přijatelná bolest	[ ]	Velmi nápadná bolest podobající se píchnutí injekce
**MÍRNÁ BOLEST** Neslučitelná s mnoha aktivitami	Zneklidňující bolest	[ ]	Silná, hluboká bolest
	Velmi zneklidňující bolest	[ ]	Silná, hluboká, pronikavá bolest, např. vyvrknutý kotník
	Intenzivní bolest	[ ]	Ještě silnější bolest srovnatelná s bodnutím několika včelích žihadel
**TĚŽKÁ BOLEST** Nejste schopni fungovat	Velmi intenzivní bolest	[ ]	Bolest srovnatelná s průměrnou migrénou
	Naprosto hrozná bolest	[ ]	Bolest srovnatelná s porodem nebo opravdu silnou migrénou
	Nesnesitelná bolest	[ ]	Bolest nelze ignorovat, vyžaduje léky proti bolesti, nutné navštívit lékaře

Jak byste posoudil/a Vaši obvyklou míru zrakové pozornosti nebo výdrže při sledování běžného celovečerního filmu?

__sleduji bez problémů__ / sleduji s přestávkami / nemůžu sledovat / nezajímá mne to

Děkujeme Vám za účast v experimentu a při vyplnění dotazníku...

MĚŘENÍ NEPROVEDENO

P11

Informace, dotazník a protokol experimentu pro
Bezkontaktní ovládání polohovacího lůžka

řešitel: Ing. Martin Kopeček, MEng, kopecema@lfhk.cuni.cz

Dotazník vyplňte co možná nejdříve po provedení testu.

Otázky pro instruktora

Experiment proveden dne: 11.5.2021

Jméno instruktora: MK

Jméno pacienta: Pohlaví: Ž Věk: 43

Číslo logu aplikace: 1,2,3

Typ omezení: Paraparéza, Paraplegie, Kvadruplegie, Pentaplegie, Svalové dystrofie, ALS,

Jiné: RS

Popis hybnosti: PLNÁ PLEGIE, RUCE + NOHY ∅
ŠPATNÝ ZRAK

Brýle / kontaktní čočky: BRÝLE PRO ano / (ne)

Protokol experimentu:

1. Kalibrace senzoru ☐ X
2. Vysvětlení principu a umožnění získat zkušenost s ovládáním, alespoň 10 minut ☒
3. Uvedení aplikaci do počátečních podmínek – lůžko v bodě nula ☒

Čas při započetí testování: ....

4. **Test 1** ☒
   a) zahájit oční kontakt s aplikací
   b) **zvednout** podpěru nohou (po dobu 2-3 s)
   c) **zvednout** podpěru hlavy (po dobu 2-3 s)
   d) **snížit** podpěru nohou (po dobu 2-3 s)
   e) **snížit** podpěru hlavy (po dobu 2-3 s)
   f) přerušit oční kontakt s aplikací
7. **Test 2** – opakování **Testu 1** ☒
8. **Test 3** – opakování **Testu 1** ☒

Čas při ukončení testování: ....

Poznámky instruktora k experimentu:

ASTIGMATISMUS + TUPOZRAKOST

NELZE NAKALIBROVAT

MOC ÚZKÁ OČNÍ ŠTĚRBINA

SEKUND. PROGRES. RS

P11

Otázky pro pacienta

Získal/a jste jistotu v ovládání? *ano / spíše ano/ nevím / spíše ne / ne*

Jak jste spokojen/a s ovládáním aplikace?

aktivace ovládání *velmi snadná / snadná / běžná / obtížná / velmi obtížná*

způsob volby polohy *velmi snadný / snadný / běžný / obtížný / velmi obtížný*

informace o nastavení lůžka *velmi snadné / snadné / běžné / obtížné / velmi obtížné*

přehlednost ovládání *velmi přehledné / přehledné/ běžné / nepřehledné/ velmi nepřehledné*

Upřednostnil/a byste ovládání jiným způsobem? *ano / spíše ano/ nevím / spíše ne / ne*

Pokud ano jakým (např. řeč)? ......DOMA NEFUNGUJE TOBII......

Máte nějaký poznatek k testu nebo ovládání?

....

....

Jak byste hodnotil/a Vaši aktuální intenzitu bolesti na uvedené obrazové škále?

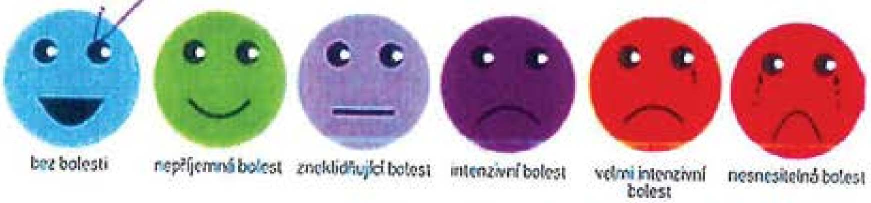

**MALÁ NEBO ŽÁDNÁ BOLEST** Jste schopni se bolesti přizpůsobit	Velmi mírná bolest	☐	Velmi lehká, sotva znatelná bolest
	Nepříjemná bolest	☐	Menší bolest, lze ji přirovnat k lehkému štípání
	Přijatelná bolest	☐	Velmi nápadná bolest podobající se píchnutí injekce
**MÍRNÁ BOLEST** Neslučitelná s mnoha aktivitami	Zneklidňující bolest	☐	Silná, hluboká bolest
	Velmi zneklidňující bolest	☐	Silná, hluboká, pronikavá bolest, např. vyvrknutý kotník
	Intenzivní bolest	☐	Ještě silnější bolest srovnatelná s bodnutím několika včelích žihadel
**TĚŽKÁ BOLEST** Nejste schopni fungovat	Velmi intenzivní bolest	☐	Bolest srovnatelná s průměrnou migrénou
	Naprosto hrozná bolest	☐	Bolest srovnatelná s porodem nebo opravdu silnou migrénou
	Nesnesitelná bolest	☐	Bolest nelze ignorovat, vyžaduje léky proti bolesti, nutné navštívit lékaře

Jak byste posoudil/a Vaši obvyklou míru zrakové pozornosti nebo výdrže při sledování běžného celovečerního filmu?

sleduji bez problémů / sleduji s přestávkami / nemůžu sledovat / nezajímá mne to

Děkujeme Vám za účast v experimentu a při vyplnění dotazníku...

P12

Informace, dotazník a protokol experimentu pro
Bezkontaktní ovládání polohovacího lůžka

řešitel: Ing. Martin Kopeček, MEng, kopecema@lfhk.cuni.cz

Dotazník vyplňte co možná nejdříve po provedení testu.

Otázky pro instruktora

Experiment proveden dne: 11.5.2021

Jméno instruktora: MK

Jméno pacienta: Pohlaví: Ž Věk: 47

Číslo logu aplikace: 1,2,3

Typ omezení: Paraparéza, Paraplegie, Kvadruplegie, Pentaplegie, Svalové dystrofie, ALS,

Jiné: RS

Popis hybnosti: ČÁSTEČNĚ POHYB RUKOU, ALE SLABÝ STISK; PŘI NEMOCI POHYB Ø

Brýle / kontaktní čočky: ano / ne (zakroužkováno: ne)

Protokol experimentu:

1. Kalibrace senzoru ☑
2. Vysvětlení principu a umožnění získat zkušenost s ovládáním, alespoň 10 minut ☑
3. Uvedení aplikaci do počátečních podmínek – lůžko v bodě nula ☑

Čas při započetí testování: 14:05

4. **Test 1** ☑
   a) zahájit oční kontakt s aplikací
   b) **zvednout** podpěru nohou (po dobu 2-3 s)
   c) **zvednout** podpěru hlavy (po dobu 2-3 s)
   d) **snížit** podpěru nohou (po dobu 2-3 s)
   e) **snížit** podpěru hlavy (po dobu 2-3 s)
   f) přerušit oční kontakt s aplikací
7. **Test 2** – opakování **Testu 1** ☑
8. **Test 3** – opakování **Testu 1** ☑

Čas při ukončení testování: 14:17

Poznámky instruktora k experimentu: VÝBORNÁ ORIENTACE

P12

Otázky pro pacienta

Získal/a jste jistotu v ovládání? ano / spíše ano / nevím / spíše ne / ne

Jak jste spokojen/a s ovládáním aplikace?

aktivace ovládání *velmi snadná / snadná / běžná / obtížná / velmi obtížná*

způsob volby polohy *velmi snadný / snadný / běžný / obtížný / velmi obtížný*

informace o nastavení lůžka *velmi snadné / snadné / běžné / obtížné / velmi obtížné*

přehlednost ovládání *velmi přehledné / přehledné / běžné / nepřehledné / velmi nepřehledné*

Upřednostnil/a byste ovládání jiným způsobem? *ano / spíše ano / nevím / spíše ne / ne*

Pokud ano jakým (např. řeč)? .... POMOCÍ RUKOU KDYBY TO ŠLO HLASOVĚ, ALE JE ZDE OMEZENÍ ŘEČI,

Máte nějaký poznatek k testu nebo ovládání?

V TÉTO POLOZE JEDNODUŠŠÍ OČIMA

Jak byste hodnotil/a Vaši aktuální intenzitu bolesti na uvedené obrazové škále?

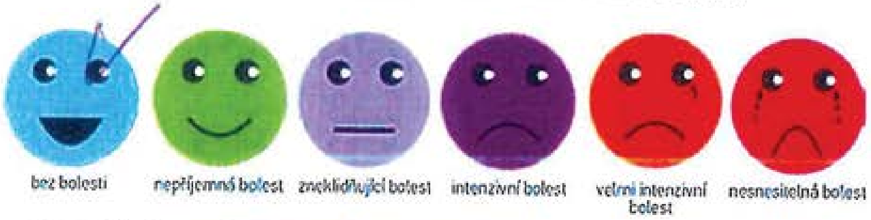

**MALÁ NEBO ŽÁDNÁ BOLEST** Jste schopni se bolesti přizpůsobit	Velmi mírná bolest	☐	Velmi lehká, sotva znatelná bolest
	Nepříjemná bolest	☐	Menší bolest, lze ji přirovnat k lehkému štípání
	Přijatelná bolest	☐	Velmi nápadná bolest podobající se píchnutí injekce
**MÍRNÁ BOLEST** Neslučitelná s mnoha aktivitami	Znekliďňující bolest	☐	Silná, hluboká bolest
	Velmi znekliďňující bolest	☐	Silná, hluboká, pronikavá bolest, např. vyvrknutý kotník
	Intenzivní bolest	☐	Ještě silnější bolest srovnatelná s bodnutím několika včelích žihadel
**TĚŽKÁ BOLEST** Nejste schopni fungovat	Velmi intenzivní bolest	☐	Bolest srovnatelná s průměrnou migrénou
	Naprosto hrozná bolest	☐	Bolest srovnatelná s porodem nebo opravdu silnou migrénou
	Nesnesitelná bolest	☐	Bolest nelze ignorovat, vyžaduje léky proti bolesti, nutné navštívit lékaře

Jak byste posoudil/a Vaši obvyklou míru zrakové pozornosti nebo výdrže při sledování běžného celovečerního filmu?

sleduji bez problémů / sleduji s přestávkami / nemůžu sledovat / nezajímá mne to

Děkujeme Vám za účast v experimentu a při vyplnění dotazníku...

P13

Informace, dotazník a protokol experimentu pro
Bezkontaktní ovládání polohovacího lůžka

řešitel: Ing. Martin Kopeček, MEng, kopecema@lfhk.cuni.cz

Dotazník vyplňte co možná nejdříve po provedení testu.

Otázky pro instruktora

Experiment proveden dne: 12.5.21

Jméno instruktora: MK

Jméno pacienta: Pohlaví: muž Věk: 63

Číslo logu aplikace: 443

Typ omezení: Paraparéza, Paraplegie, Kvadruplegie, Pentaplegie, Svalové dystrofie, ALS, RS

Jiné: RS

Popis hybnosti: DK plegie, HK část. motor, slabý úchop - menší stisk tlačítka

Brýle / kontaktní čočky: brýle jen čtení ano (ne)

Protokol experimentu:

1. Kalibrace senzoru ☑
2. Vysvětlení principu a umožnění získat zkušenost s ovládáním, alespoň 10 minut ☑
3. Uvedení aplikaci do počátečních podmínek – lůžko v bodě nula ☑

   Čas při započetí testování: 8,25

4. **Test 1** ☑
   a) zahájit oční kontakt s aplikací
   b) **zvednout** podpěru nohou (po dobu 2-3 s)
   c) **zvednout** podpěru hlavy (po dobu 2-3 s)
   d) **snížit** podpěru nohou (po dobu 2-3 s)
   e) **snížit** podpěru hlavy (po dobu 2-3 s)
   f) přerušit oční kontakt s aplikací

7. **Test 2** – opakování **Testu 1** ☑
8. **Test 3** – opakování **Testu 1** ☑

   Čas při ukončení testování: 8,35

Poznámky instruktora k experimentu:

(strabismus V. Hrabec r. 58
(LO vidí jako P - ale musí

8.   P13

Otázky pro pacienta

Získal/a jste jistotu v ovládání? *(ano) / spíše ano / nevím / spíše ne / ne*

Jak jste spokojen/a s ovládáním aplikace?

- aktivace ovládání *(velmi snadná) / snadná / běžná / obtížná / velmi obtížná*
- způsob volby polohy *(velmi snadný) / snadný / běžný / obtížný / velmi obtížný*
- informace o nastavení lůžka *(velmi snadné) / snadné / běžné / obtížné / velmi obtížné*
- přehlednost ovládání *(velmi přehledné) / přehledné / běžné / nepřehledné / velmi nepřehledné*

Raději než tlačítky

Upřednostnil/a byste ovládání jiným způsobem? *ano / spíše ano / nevím / spíše ne / (ne)*

Pokud ano jakým (např. řeč)? ....

Máte nějaký poznatek k testu nebo ovládání?

Pán byl velmi spokojen s vysvětlením, příjemný a rozumný.

Jak byste hodnotil/a Vaši aktuální intenzitu bolesti na uvedené obrazové škále?

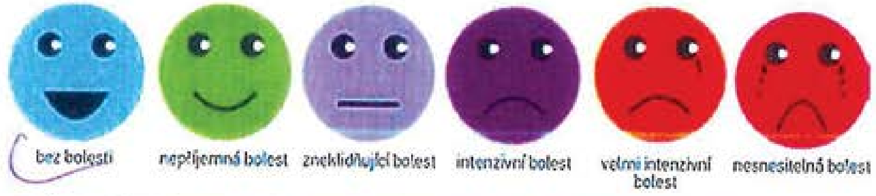

**MALÁ NEBO ŽÁDNÁ BOLEST** Jste schopni se bolesti přizpůsobit	Velmi mírná bolest	☐	Velmi lehká, sotva znatelná bolest
	Nepříjemná bolest	☐	Menší bolest, lze ji přirovnat k lehkému štípání
	Přijatelná bolest	☐	Velmi nápadná bolest podobající se píchnutí injekce
**MÍRNÁ BOLEST** Neslučitelná s mnoha aktivitami	Zneklidňující bolest	☐	Silná, hluboká bolest
	Velmi zneklidňující bolest	☐	Silná, hluboká, pronikavá bolest, např. vyvrknutý kotník
	Intenzivní bolest	☐	Ještě silnější bolest srovnatelná s bodnutím několika včelích žihadel
**TĚŽKÁ BOLEST** Nejste schopni fungovat	Velmi intenzivní bolest	☐	Bolest srovnatelná s průměrnou migrénou
	Naprosto hrozná bolest	☐	Bolest srovnatelná s porodem nebo opravdu silnou migrénou
	Nesnesitelná bolest	☐	Bolest nelze ignorovat, vyžaduje léky proti bolesti, nutné navštívit lékaře

Jak byste posoudil/a Vaši obvyklou míru zrakové pozornosti nebo výdrže při sledování běžného celovečerního filmu?

(sleduji bez problémů) / sleduji s přestávkami / nemůžu sledovat / nezajímá mne to

Děkujeme Vám za účast v experimentu a při vyplnění dotazníku...

HMP

P14

Informace, dotazník a protokol experimentu pro
Bezkontaktní ovládání polohovacího lůžka

řešitel: Ing. Martin Kopeček, MEng, kopecema@lfhk.cuni.cz

Dotazník vyplňte co možná nejdříve po provedení testu.

Otázky pro instruktora

Experiment proveden dne: 12.5.2021

Jméno instruktora: MK

Jméno pacienta: [illegible] Pohlaví: žena Věk: 60

Číslo logu aplikace: 23

Typ omezení: Paraparéza, Paraplegie, Kvadruplegie, Pentaplegie, Svalové dystrofie, ALS,

Jiné: RS operace míchy – nádor, lordóza, částečné ochrnutí

Popis hybnosti: částečné ochr., stisk rukou dobrý, DK – pleg.

Brýle / kontaktní čočky: čtení, ano / ne (zakroužkováno: ne)

Protokol experimentu:

1. Kalibrace senzoru ☑
2. Vysvětlení principu a umožnění získat zkušenost s ovládáním, alespoň 10 minut ☑
3. Uvedení aplikaci do počátečních podmínek – lůžko v bodě nula ☑

Čas při započetí testování: 9:40

4. **Test 1** ☑
   a) zahájit oční kontakt s aplikací
   b) **zvednout** podpěru nohou (po dobu 2-3 s)
   c) **zvednout** podpěru hlavy (po dobu 2-3 s)
   d) **snížit** podpěru nohou (po dobu 2-3 s)
   e) **snížit** podpěru hlavy (po dobu 2-3 s)
   f) přerušit oční kontakt s aplikací
7. **Test 2** – opakování **Testu 1** ☑
8. **Test 3** – opakování **Testu 1** ☑

Čas při ukončení testování: 9:55

Poznámky instruktora k experimentu:

P14

Otázky pro pacienta

Získal/a jste jistotu v ovládání? ano / **spíše ano** / nevím / spíše ne / ne

Jak jste spokojen/a s ovládáním aplikace?

- aktivace ovládání — velmi snadná / **snadná** / běžná / obtížná / velmi obtížná
- způsob volby polohy — velmi snadný / **snadný** / běžný / obtížný / velmi obtížný
- informace o nastavení lůžka — velmi snadné / **snadné** / běžné / obtížné / velmi obtížné
- přehlednost ovládání — **velmi přehledné** / přehledné/ běžné / nepřehledné/ velmi nepřehledné

Upřednostnil/a byste ovládání jiným způsobem? ano / spíše ano/ **nevím** / spíše ne / ne

Pokud ano jakým (např. řeč)? zatím [illegible] řešení

Máte nějaký poznatek k testu nebo ovládání?

Vše v pořádku

Jak byste hodnotil/a Vaši aktuální intenzitu bolesti na uvedené obrazové škále? duševní nepohoda (rodinné důvody)

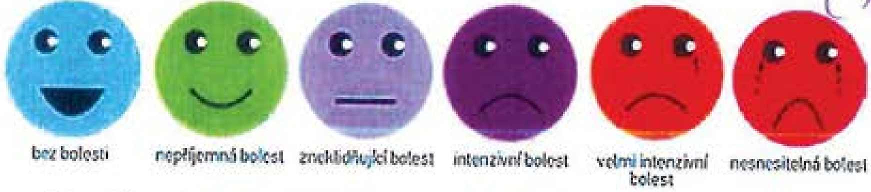

**MALÁ NEBO ŽÁDNÁ BOLEST** Jste schopni se bolesti přizpůsobit	Velmi mírná bolest	☐	Velmi lehká, sotva znatelná bolest
	Nepříjemná bolest	☐	Menší bolest, lze ji přirovnat k lehkému štípání
	Přijatelná bolest	☐	Velmi nápadná bolest podobající se píchnutí injekce
**MÍRNÁ BOLEST** Neslučitelná s mnoha aktivitami	Zneklidňující bolest	☐	Silná, hluboká bolest
	Velmi zneklidňující bolest	☐	Silná, hluboká, pronikavá bolest, např. vyvrknutý kotník
	Intenzivní bolest	☐	Ještě silnější bolest srovnatelná s bodnutím několika včelích žihadel
**TĚŽKÁ BOLEST** Nejste schopni fungovat	Velmi intenzivní bolest	☐	Bolest srovnatelná s průměrnou migrénou
	Naprosto hrozná bolest	☐	Bolest srovnatelná s porodem nebo opravdu silnou migrénou
	Nesnesitelná bolest	☐	Bolest nelze ignorovat, vyžaduje léky proti bolesti, nutné navštívit lékaře

Jak byste posoudil/a Vaši obvyklou míru zrakové pozornosti nebo výdrže při sledování běžného celovečerního filmu?

sleduji bez problémů / sleduji s přestávkami / nemůžu sledovat / nezajímá mne to

Děkujeme Vám za účast v experimentu a při vyplnění dotazníku...

P15

Bezkontaktní ovládání polohovacího lůžka

řešitel: Ing. Martin Kopeček, MEng, kopecema@lfhk.cuni.cz

Dotazník vyplňte co možná nejdříve po provedení testu.

Otázky pro instruktora

Experiment proveden dne: 12.5. 2021

Jméno instruktora: MK

Jméno pacienta: Pohlaví: muž Věk: 42

Číslo logu aplikace: 3

Typ omezení: Paraparéza, Paraplegie, Kvadruplegie, Pentaplegie, Svalové dystrofie, ALS,

Jiné: RS

Popis hybnosti: PHK dost. hybnost - mírný stisk, plegie progres

Brýle / kontaktní čočky: zúž. zor. pole (omezení - dvojité vid. stranové vidění - poruš. zor. pole) ano / ~~ne~~ (ne)

Protokol experimentu:

1. Kalibrace senzoru ☑
2. Vysvětlení principu a umožnění získat zkušenost s ovládáním, alespoň 10 minut ☑
3. Uvedení aplikaci do počátečních podmínek – lůžko v bodě nula ☑

   Čas při započetí testování: 10:22

4. **Test 1** ☑
   - a) zahájit oční kontakt s aplikací
   - b) **zvednout** podpěru nohou (po dobu 2-3 s)
   - c) **zvednout** podpěru hlavy (po dobu 2-3 s)
   - d) **snížit** podpěru nohou (po dobu 2-3 s)
   - e) **snížit** podpěru hlavy (po dobu 2-3 s)
   - f) přerušit oční kontakt s aplikací

7. **Test 2** – opakování **Testu 1** ☑
8. **Test 3** – opakování **Testu 1** ☑

   Čas při ukončení testování: 10:28

Poznámky instruktora k experimentu:

Otázky pro pacienta

Získal/a jste jistotu v ovládání? **ano** / spíše ano / nevím / spíše ne / ne

Jak jste spokojen/a s ovládáním aplikace?

- aktivace ovládání — **velmi snadná** / snadná / běžná / obtížná / velmi obtížná
- způsob volby polohy — **velmi snadný** / snadný / běžný / obtížný / velmi obtížný
- informace o nastavení lůžka — velmi snadné / **snadné** / běžné / obtížné / velmi obtížné
  prodleva při zpr. lůž. oproti virtuál. příkazu
- přehlednost ovládání — **velmi přehledné** / přehledné / běžné / nepřehledné / velmi nepřehledné

Upřednostnil/a byste ovládání jiným způsobem? ano / spíše ano / **nevím** / spíše ne / ne

Pokud ano jakým (např. řeč)? ....

Máte nějaký poznatek k testu nebo ovládání? (pac. má úst. myš., přímka oči – je tímto mucem oči těžké obrat)

velikost i barva segmentu dobrá, barvy nejsou křiklavé, což je dobře

Jak byste hodnotil/a Vaši aktuální intenzitu bolesti na uvedené obrazové škále?

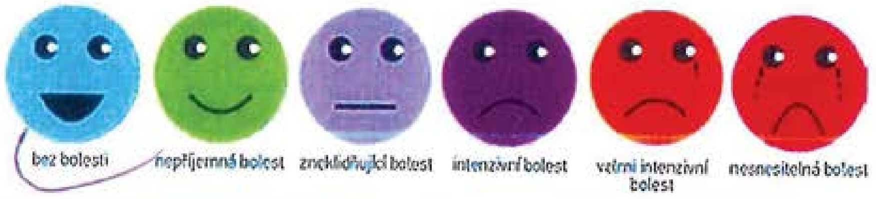

MALÁ NEBO ŽÁDNÁ BOLEST<br>Jste schopni se bolesti přizpůsobit	Velmi mírná bolest	☐	Velmi lehká, sotva znatelná bolest
	Nepříjemná bolest	☐	Menší bolest, lze ji přirovnat k lehkému štípání
	Přijatelná bolest	☐	Velmi nápadná bolest podobající se píchnutí injekce
MÍRNÁ BOLEST<br>Neslučitelná s mnoha aktivitami	Zneklidňující bolest	☐	Silná, hluboká bolest
	Velmi zneklidňující bolest	☐	Silná, hluboká, pronikavá bolest, např. vyvrknutý kotník
	Intenzivní bolest	☐	Ještě silnější bolest srovnatelná s bodnutím několika včelích žihadel
TĚŽKÁ BOLEST<br>Nejste schopni fungovat	Velmi intenzivní bolest	☐	Bolest srovnatelná s průměrnou migrénou
	Naprosto hrozná bolest	☐	Bolest srovnatelná s porodem nebo opravdu silnou migrénou
	Nesnesitelná bolest	☐	Bolest nelze ignorovat, vyžaduje léky proti bolesti, nutné navštívit lékaře

Jak byste posoudil/a Vaši obvyklou míru zrakové pozornosti nebo výdrže při sledování běžného celovečerního filmu?

sleduji bez problémů / sleduji s přestávkami / nemůžu sledovat / nezajímá mne to

Děkujeme Vám za účast v experimentu a při vyplnění dotazníku...

2MF P16

Informace, dotazník a protokol experimentu pro
Bezkontaktní ovládání polohovacího lůžka

řešitel: Ing. Martin Kopeček, MEng, kopecema@lfhk.cuni.cz

Dotazník vyplňte co možná nejdříve po provedení testu.

Otázky pro instruktora

Experiment proveden dne: 12.5. 2021

Jméno instruktora: MK

Jméno pacienta: Pohlaví: muž Věk: 68

Číslo logu aplikace: [illegible]

Typ omezení: Paraparéza, Paraplegie, Kvadruplegie, Pentaplegie, Svalové dystrofie, ALS,

Jiné: RS

Popis hybnosti: PHK - plegie
nedostatečný stisk, plegie celková

Brýle / kontaktní čočky: ano / (ne)

Protokol experimentu:

1. Kalibrace senzoru ☑
2. Vysvětlení principu a umožnění získat zkušenost s ovládáním, alespoň 10 minut ☑
3. Uvedení aplikaci do počátečních podmínek – lůžko v bodě nula ☑

   Čas při započetí testování: 13:00

4. **Test 1** ☑
   a) zahájit oční kontakt s aplikací
   b) **zvednout** podpěru nohou (po dobu 2-3 s)
   c) **zvednout** podpěru hlavy (po dobu 2-3 s)
   d) **snížit** podpěru nohou (po dobu 2-3 s)
   e) **snížit** podpěru hlavy (po dobu 2-3 s)
   f) přerušit oční kontakt s aplikací

7. **Test 2** – opakování **Testu 1** ☑
8. **Test 3** – opakování **Testu 1** ☑

   Čas při ukončení testování: 13:05

Poznámky instruktora k experimentu:

P16

Otázky pro pacienta

Získal/a jste jistotu v ovládání? ano / **spíše ano** / nevím / spíše ne / ne

Jak jste spokojen/a s ovládáním aplikace?

aktivace ovládání *velmi snadná / **snadná** / běžná / obtížná / velmi obtížná*

způsob volby polohy *velmi snadný / **snadný** / běžný / obtížný / velmi obtížný*

informace o nastavení lůžka *__velmi snadné__ / snadné / běžné / obtížné / velmi obtížné*

přehlednost ovládání *velmi přehledné / **přehledné** / běžné / nepřehledné/ velmi nepřehledné*

Upřednostnil/a byste ovládání jiným způsobem? *ano / spíše ano/ nevím / spíše ne / **ne***

Pokud ano jakým (např. řeč)? ....

Máte nějaký poznatek k testu nebo ovládání?

Spokojenost s možností nast. regulace velikost i barvy [illegible]

Jak byste hodnotil/a Vaši aktuální intenzitu bolesti na uvedené obrazové škále?

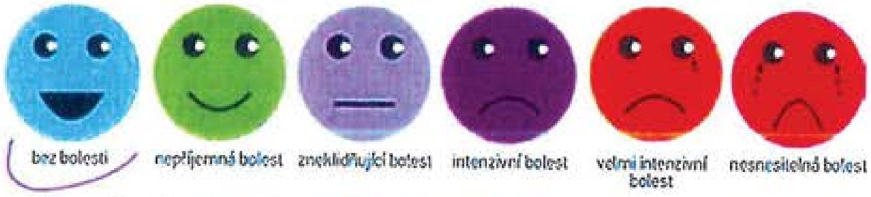

MALÁ NEBO ŽÁDNÁ BOLEST Jste schopni se bolesti přizpůsobit	Velmi mírná bolest	☐	Velmi lehká, sotva znatelná bolest
	Nepříjemná bolest	☐	Menší bolest, lze ji přirovnat k lehkému štípání
	Přijatelná bolest	☐	Velmi nápadná bolest podobající se píchnutí injekce
MÍRNÁ BOLEST Neslučitelná s mnoha aktivitami	Zneklidňující bolest	☐	Silná, hluboká bolest
	Velmi zneklidňující bolest	☐	Silná, hluboká, pronikavá bolest, např. vyvrknutý kotník
	Intenzivní bolest	☐	Ještě silnější bolest srovnatelná s bodnutím několika včelích žihadel
TĚŽKÁ BOLEST Nejste schopni fungovat	Velmi intenzivní bolest	☐	Bolest srovnatelná s průměrnou migrénou
	Naprosto hrozná bolest	☐	Bolest srovnatelná s porodem nebo opravdu silnou migrénou
	Nesnesitelná bolest	☐	Bolest nelze ignorovat, vyžaduje léky proti bolesti, nutné navštívit lékaře

Jak byste posoudil/a Vaši obvyklou míru zrakové pozornosti nebo výdrže při sledování běžného celovečerního filmu?

__sleduji bez problémů__ / sleduji s přestávkami / nemůžu sledovat / nezajímá mne to

Děkujeme Vám za účast v experimentu a při vyplnění dotazníku...

P17

Informace, dotazník a protokol experimentu pro

Bezkontaktní ovládání polohovacího lůžka

řešitel: Ing. Martin Kopeček, MEng, kopecema@lfhk.cuni.cz

Dotazník vyplňte co možná nejdříve po provedení testu.

Otázky pro instruktora

Experiment proveden dne: 12. 5. 2021

Jméno instruktora: MK

Jméno pacienta: Pohlaví: žena Věk: 58

Číslo logu aplikace: 1,2,3

Typ omezení: Paraparéza, Paraplegie, Kvadruplegie, Pentaplegie, Svalové dystrofie, ALS,

Jiné: RS

Popis hybnosti: paží zach. hybnost, stisk omezen - slabý, bez mož. stisku tlačítka (proběhlé operace - páteř, ruce) porucha nohou (částeč. par. nohou)

Brýle / kontaktní čočky: ~~dalekozrakost~~ krátkozrakost, ano (ne)

Protokol experimentu:

1. Kalibrace senzoru ☑
2. Vysvětlení principu a umožnění získat zkušenost s ovládáním, alespoň 10 minut ☑
3. Uvedení aplikaci do počátečních podmínek – lůžko v bodě nula ☑

Čas při započetí testování: 14:08

4. **Test 1** ☑
   a) zahájit oční kontakt s aplikací
   b) **zvednout** podpěru nohou (po dobu 2-3 s)
   c) **zvednout** podpěru hlavy (po dobu 2-3 s)
   d) **snížit** podpěru nohou (po dobu 2-3 s)
   e) **snížit** podpěru hlavy (po dobu 2-3 s)
   f) přerušit oční kontakt s aplikací

7. **Test 2** – opakování **Testu 1** ☑
8. **Test 3** – opakování **Testu 1** ☑

Čas při ukončení testování: 14:15

Poznámky instruktora k experimentu:

F.9 P17

Otázky pro pacienta

Získal/a jste jistotu v ovládání? ano / **(spíše ano)** / nevím / spíše ne / ne

Jak jste spokojen/a s ovládáním aplikace?

- aktivace ovládání — velmi snadná / **(snadná)** / běžná / obtížná / velmi obtížná
- způsob volby polohy — velmi snadný / **(snadný)** / běžný / obtížný / velmi obtížný
- informace o nastavení lůžka — velmi snadné / **(snadné)** / běžné / obtížné / velmi obtížné
- přehlednost ovládání — **(velmi přehledné)** / přehledné/ běžné / nepřehledné/ velmi nepřehledné

Upřednostnil/a byste ovládání jiným způsobem? ano / spíše ano/ nevím / spíše ne / **(ne)**

Pokud ano jakým (např. řeč)? ....

Máte nějaký poznatek k testu nebo ovládání?

rozšíření ovládání o další [mi bylo v pořádku] funkce (rehabilitač) - při dlouhém ležení, ...

Jak byste hodnotil/a Vaši aktuální intenzitu bolesti na uvedené obrazové škále?

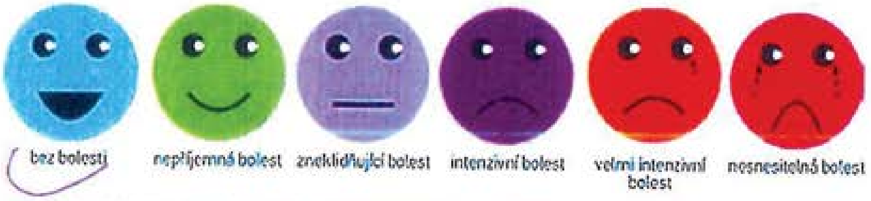

**MALÁ NEBO ŽÁDNÁ BOLEST** Jste schopni se bolesti přizpůsobit	Velmi mírná bolest	☐	Velmi lehká, sotva znatelná bolest
	Nepříjemná bolest	☐	Menší bolest, lze ji přirovnat k lehkému štípání
	Přijatelná bolest	☐	Velmi nápadná bolest podobající se píchnutí injekce
**MÍRNÁ BOLEST** Neslučitelná s mnoha aktivitami	Znekliďňující bolest	☐	Silná, hluboká bolest
	Velmi znekliďňující bolest	☐	Silná, hluboká, pronikavá bolest, např. vyvrknutý kotník
	Intenzivní bolest	☐	Ještě silnější bolest srovnatelná s bodnutím několika včelích žihadel
**TĚŽKÁ BOLEST** Nejste schopni fungovat	Velmi intenzivní bolest	☐	Bolest srovnatelná s průměrnou migrénou
	Naprosto hrozná bolest	☐	Bolest srovnatelná s porodem nebo opravdu silnou migrénou
	Nesnesitelná bolest	☐	Bolest nelze ignorovat, vyžaduje léky proti bolesti, nutné navštívit lékaře

Jak byste posoudil/a Vaši obvyklou míru zrakové pozornosti nebo výdrže při sledování běžného celovečerního filmu?

(sleduji bez problémů) / sleduji s přestávkami / nemůžu sledovat / nezajímá mne to

Děkujeme Vám za účast v experimentu a při vyplnění dotazníku...

C1

Informace, dotazník a protokol experimentu pro
Bezkontaktní ovládání polohovacího lůžka

řešitel: Ing. Martin Kopeček, MEng, kopecema@lfhk.cuni.cz

Dotazník vyplňte co možná nejdříve po provedení testu.

Otázky pro instruktora

Experiment proveden dne: 17. 06. 2020

Jméno instruktora: KOPEČEK

Jméno pacienta: Pohlaví: Ž Věk: 20

Číslo logu aplikace:

Typ omezení: Paraparéza, Paraplegie, Kvadruplegie, Pentaplegie, Svalové dystrofie, ALS,

Jiné: žádné

Popis hybnosti: plná

Brýle / kontaktní čočky: ASTIGMAT. (ano) / ne

Protokol experimentu:

1. Kalibrace senzoru ☑
2. Vysvětlení principu a umožnění získat zkušenost s ovládáním, alespoň 10 minut ☑
3. Uvedení aplikaci do počátečních podmínek – lůžko v bodě nula ☑

Čas při započetí testování: 13 45

4. **Test 1** ☑
   a) zahájit oční kontakt s aplikací
   b) **zvednout** podpěru nohou (po dobu 2-3 s)
   c) **zvednout** podpěru hlavy (po dobu 2-3 s)
   d) **snížit** podpěru nohou (po dobu 2-3 s)
   e) **snížit** podpěru hlavy (po dobu 2-3 s)
   f) přerušit oční kontakt s aplikací
7. **Test 2** – opakování **Testu 1** ☑
8. **Test 3** – opakování **Testu 1** ☑

Čas při ukončení testování: 14 00

Poznámky instruktora k experimentu:

C1

Otázky pro pacienta

Získal/a jste jistotu v ovládání? *ano / spíše ano/ nevím / spíše ne / ne*

Jak jste spokojen/a s ovládáním aplikace?

- aktivace ovládání *velmi snadná / snadná / běžná / obtížná / velmi obtížná*
- způsob volby polohy *velmi snadný / snadný / běžný / obtížný / velmi obtížný*
- informace o nastavení lůžka *velmi snadné / snadné / běžné / obtížné / velmi obtížné*
- přehlednost ovládání *velmi přehledné / přehledné/ běžné / nepřehledné/ velmi nepřehledné*

Upřednostnil/a byste ovládání jiným způsobem? *ano / spíše ano/ nevím / spíše ne / ne*

Pokud ano jakým (např. řeč)? ....

Máte nějaký poznatek k testu nebo ovládání?

____

____

Jak byste hodnotil/a Vaši aktuální intenzitu bolesti na uvedené obrazové škále?

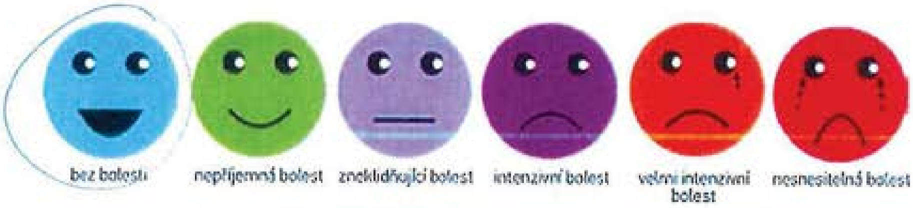

**MALÁ NEBO ŽÁDNÁ BOLEST** Jste schopni se bolesti přizpůsobit	Velmi mírná bolest	☐	Velmi lehká, sotva znatelná bolest
	Nepříjemná bolest	☐	Menší bolest, lze ji přirovnat k lehkému štípání
	Přijatelná bolest	☐	Velmi nápadná bolest podobající se píchnutí injekce
**MÍRNÁ BOLEST** Neslučitelná s mnoha aktivitami	Zneklidňující bolest	☐	Silná, hluboká bolest
	Velmi zneklidňující bolest	☐	Silná, hluboká, pronikavá bolest, např. vyvrknutý kotník
	Intenzivní bolest	☐	Ještě silnější bolest srovnatelná s bodnutím několika včelích žihadel
**TĚŽKÁ BOLEST** Nejste schopni fungovat	Velmi intenzivní bolest	☐	Bolest srovnatelná s průměrnou migrénou
	Naprosto hrozná bolest	☐	Bolest srovnatelná s porodem nebo opravdu silnou migrénou
	Nesnesitelná bolest	☐	Bolest nelze ignorovat, vyžaduje léky proti bolesti, nutné navštívit lékaře

Jak byste posoudil/a Vaši obvyklou míru zrakové pozornosti nebo výdrže při sledování běžného celovečerního filmu?

sleduji bez problémů / sleduji s přestávkami / nemůžu sledovat / nezajímá mne to

Děkujeme Vám za účast v experimentu a při vyplnění dotazníku...

39 C2

Informace, dotazník a protokol experimentu pro

Bezkontaktní ovládání polohovacího lůžka

řešitel: Ing. Martin Kopeček, MEng, kopecema@lfhk.cuni.cz

Dotazník vyplňte co možná nejdříve po provedení testu.

Otázky pro instruktora

Experiment proveden dne: 17. 6. 2020

Jméno instruktora: KOPEČEK

Jméno pacienta: [illegible] Pohlaví: Ž Věk: 20

Číslo logu aplikace: –

Typ omezení: Paraparéza, Paraplegie, Kvadruplegie, Pentaplegie, Svalové dystrofie, ALS,

Jiné: žádné

Popis hybnosti: plná

Brýle / kontaktní čočky: ano / ~~ne~~ (ne)

Protokol experimentu:

1. Kalibrace senzoru ☑
2. Vysvětlení principu a umožnění získat zkušenost s ovládáním, alespoň 10 minut ☑
3. Uvedení aplikaci do počátečních podmínek – lůžko v bodě nula ☑

   Čas při započetí testování: 12^50

4. **Test 1** ☑
   a) zahájit oční kontakt s aplikací
   b) **zvednout** podpěru nohou (po dobu 2-3 s)
   c) **zvednout** podpěru hlavy (po dobu 2-3 s)
   d) **snížit** podpěru nohou (po dobu 2-3 s)
   e) **snížit** podpěru hlavy (po dobu 2-3 s)
   f) přerušit oční kontakt s aplikací

7. **Test 2** – opakování **Testu 1** ☑
8. **Test 3** – opakování **Testu 1** ☑

   Čas při ukončení testování: 12^45

Poznámky instruktora k experimentu:

C2

Otázky pro pacienta

Získal/a jste jistotu v ovládání? ano / spíše ano/ nevím / spíše ne / ne

Jak jste spokojen/a s ovládáním aplikace?

- aktivace ovládání — velmi snadná / snadná / běžná / obtížná / velmi obtížná
- způsob volby polohy — velmi snadný / snadný / běžný / obtížný / velmi obtížný
- informace o nastavení lůžka — velmi snadné / snadné / běžné / obtížné / velmi obtížné
- přehlednost ovládání — velmi přehledné / přehledné/ běžné / nepřehledné/ velmi nepřehledné

Upřednostnil/a byste ovládání jiným způsobem? ano / spíše ano/ nevím / spíše ne / ne

Pokud ano jakým (např. řeč)? ....

Máte nějaký poznatek k testu nebo ovládání? —

Jak byste hodnotil/a Vaši aktuální intenzitu bolesti na uvedené obrazové škále?

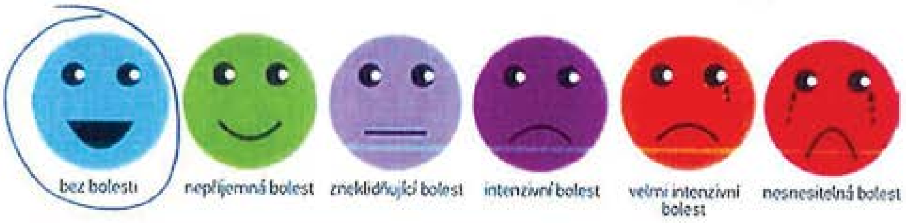

**MALÁ NEBO ŽÁDNÁ BOLEST** Jste schopni se bolesti přizpůsobit	Velmi mírná bolest	☐	Velmi lehká, sotva znatelná bolest
	Nepříjemná bolest	☐	Menší bolest, lze ji přirovnat k lehkému štípání
	Přijatelná bolest	☐	Velmi nápadná bolest podobající se píchnutí injekce
**MÍRNÁ BOLEST** Neslučitelná s mnoha aktivitami	Zneklidňující bolest	☐	Silná, hluboká bolest
	Velmi zneklidňující bolest	☐	Silná, hluboká, pronikavá bolest, např. vyvrknutý kotník
	Intenzivní bolest	☐	Ještě silnější bolest srovnatelná s bodnutím několika včelích žihadel
**TĚŽKÁ BOLEST** Nejste schopni fungovat	Velmi intenzivní bolest	☐	Bolest srovnatelná s průměrnou migrénou
	Naprosto hrozná bolest	☐	Bolest srovnatelná s porodem nebo opravdu silnou migrénou
	Nesnesitelná bolest	☐	Bolest nelze ignorovat, vyžaduje léky proti bolesti, nutné navštívit lékaře

Jak byste posoudil/a Vaši obvyklou míru zrakové pozornosti nebo výdrže při sledování běžného celovečerního filmu?

sleduji bez problémů / sleduji s přestávkami / nemůžu sledovat / nezajímá mne to

Děkujeme Vám za účast v experimentu a při vyplnění dotazníku...

C3

Informace, dotazník a protokol experimentu pro

Bezkontaktní ovládání polohovacího lůžka

řešitel: Ing. Martin Kopeček, MEng, kopecema@lfhk.cuni.cz

Dotazník vyplňte co možná nejdříve po provedení testu.

Otázky pro instruktora

Experiment proveden dne: 10. 6. 2020

Jméno instruktora: KOPEČEK

Jméno pacienta: Pohlaví: Ž Věk: 20

Číslo logu aplikace:

Typ omezení: Paraparéza, Paraplegie, Kvadruplegie, Pentaplegie, Svalové dystrofie, ALS,

Jiné: zdravá

Popis hybnosti: plná

Brýle / kontaktní čočky: ano / ne (zakroužkováno: ne)

Protokol experimentu:

1. Kalibrace senzoru ☑
2. Vysvětlení principu a umožnění získat zkušenost s ovládáním, alespoň 10 minut ☑
3. Uvedení aplikaci do počátečních podmínek – lůžko v bodě nula ☑

   Čas při započetí testování: 13:15
4. **Test 1** ☑
   a) zahájit oční kontakt s aplikací
   b) **zvednout** podpěru nohou (po dobu 2-3 s)
   c) **zvednout** podpěru hlavy (po dobu 2-3 s)
   d) **snížit** podpěru nohou (po dobu 2-3 s)
   e) **snížit** podpěru hlavy (po dobu 2-3 s)
   f) přerušit oční kontakt s aplikací
7. **Test 2** – opakování **Testu 1** ☑
8. **Test 3** – opakování **Testu 1** ☑

   Čas při ukončení testování: 13:30

Poznámky instruktora k experimentu:

C3

Otázky pro pacienta

Získal/a jste jistotu v ovládání? *ano / spíše ano / nevím / spíše ne / ne*

Jak jste spokojen/a s ovládáním aplikace?

- aktivace ovládání *velmi snadná / snadná / běžná / obtížná / velmi obtížná*
- způsob volby polohy *velmi snadný / snadný / běžný / obtížný / velmi obtížný*
- informace o nastavení lůžka *velmi snadné / snadné / běžné / obtížné / velmi obtížné*
- přehlednost ovládání *velmi přehledné / přehledné/ běžné / nepřehledné/ velmi nepřehledné*

Upřednostnil/a byste ovládání jiným způsobem? *ano / spíše ano/ nevím / spíše ne / ne*

Pokud ano jakým (např. řeč)? ....

Máte nějaký poznatek k testu nebo ovládání?

____

____

Jak byste hodnotil/a Vaši aktuální intenzitu bolesti na uvedené obrazové škále?

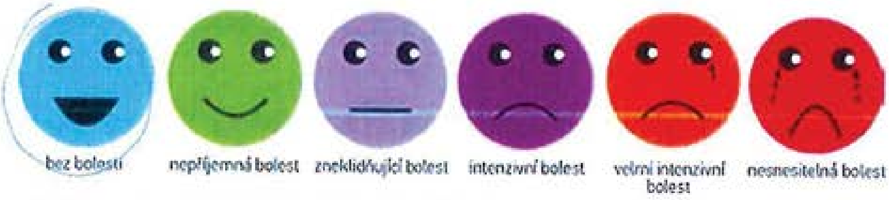

**MALÁ NEBO ŽÁDNÁ BOLEST**<br>Jste schopni se bolesti přizpůsobit	Velmi mírná bolest	☐	Velmi lehká, sotva znatelná bolest
	Nepříjemná bolest	☐	Menší bolest, lze ji přirovnat k lehkému štípání
	Přijatelná bolest	☐	Velmi nápadná bolest podobající se píchnutí injekce
**MÍRNÁ BOLEST**<br>Neslučitelná s mnoha aktivitami	Zneklidňující bolest	☐	Silná, hluboká bolest
	Velmi zneklidňující bolest	☐	Silná, hluboká, pronikavá bolest, např. vyvrknutý kotník
	Intenzivní bolest	☐	Ještě silnější bolest srovnatelná s bodnutím několika včelích žihadel
**TĚŽKÁ BOLEST**<br>Nejste schopni fungovat	Velmi intenzivní bolest	☐	Bolest srovnatelná s průměrnou migrénou
	Naprosto hrozná bolest	☐	Bolest srovnatelná s porodem nebo opravdu silnou migrénou
	Nesnesitelná bolest	☐	Bolest nelze ignorovat, vyžaduje léky proti bolesti, nutné navštívit lékaře

Jak byste posoudil/a Vaši obvyklou míru zrakové pozornosti nebo výdrže při sledování běžného celovečerního filmu?

sleduji bez problémů / sleduji s přestávkami / nemůžu sledovat / nezajímá mne to

Děkujeme Vám za účast v experimentu a při vyplnění dotazníku...

C4

Informace, dotazník a protokol experimentu pro
Bezkontaktní ovládání polohovacího lůžka

řešitel: Ing. Martin Kopeček, MEng, kopecema@lfhk.cuni.cz

Dotazník vyplňte co možná nejdříve po provedení testu.

Otázky pro instruktora

Experiment proveden dne: 10. 6. 2020

Jméno instruktora: Kopeček

Jméno pacienta: Pohlaví: Ž Věk: 19

Číslo logu aplikace:

Typ omezení: Paraparéza, Paraplegie, Kvadruplegie, Pentaplegie, Svalové dystrofie, ALS,

Jiné: žádné

Popis hybnosti: plná

Brýle / kontaktní čočky: ano / ne

Protokol experimentu:

1. Kalibrace senzoru ☑
2. Vysvětlení principu a umožnění získat zkušenost s ovládáním, alespoň 10 minut ☑
3. Uvedení aplikaci do počátečních podmínek – lůžko v bodě nula ☑

Čas při započetí testování: 13:00

4. **Test 1** ☑
   a) zahájit oční kontakt s aplikací
   b) **zvednout** podpěru nohou (po dobu 2-3 s)
   c) **zvednout** podpěru hlavy (po dobu 2-3 s)
   d) **snížit** podpěru nohou (po dobu 2-3 s)
   e) **snížit** podpěru hlavy (po dobu 2-3 s)
   f) přerušit oční kontakt s aplikací
7. **Test 2** – opakování **Testu 1** ☑
8. **Test 3** – opakování **Testu 1** ☑

Čas při ukončení testování: 13:10

Poznámky instruktora k experimentu:

#0 C4

Otázky pro pacienta

Získal/a jste jistotu v ovládání? **ano** / *spíše ano / nevím / spíše ne / ne*

Jak jste spokojen/a s ovládáním aplikace?

aktivace ovládání *velmi snadná / snadná / běžná / obtížná / velmi obtížná* (zakroužkováno: velmi snadná)

způsob volby polohy *velmi snadný / snadný / běžný / obtížný / velmi obtížný* (zakroužkováno: velmi snadný)

informace o nastavení lůžka *velmi snadné / snadné / běžné / obtížné / velmi obtížné* (zakroužkováno: velmi snadné)

přehlednost ovládání *velmi přehledné / přehledné / běžné / nepřehledné / velmi nepřehledné* (zakroužkováno: velmi přehledné)

Upřednostnil/a byste ovládání jiným způsobem? *ano / spíše ano / nevím / spíše ne / ne* (zakroužkováno: spíše ne)

Pokud ano jakým (např. řeč)? ....

Máte nějaký poznatek k testu nebo ovládání?

Úžasná metoda, pro lidi kteří nemohou svou polohu nijak jinak ovládat, stát se tak soběstačný v určování své polohy

Jak byste hodnotil/a Vaši aktuální intenzitu bolesti na uvedené obrazové škále?

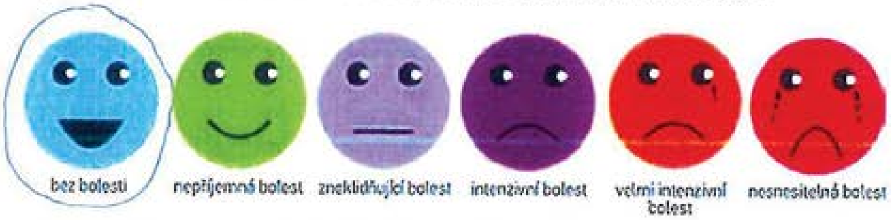

**MALÁ NEBO ŽÁDNÁ BOLEST** Jste schopni se bolesti přizpůsobit	Velmi mírná bolest	☐	Velmi lehká, sotva znatelná bolest
	Nepříjemná bolest	☐	Menší bolest, lze ji přirovnat k lehkému štípání
	Přijatelná bolest	☐	Velmi nápadná bolest podobající se píchnutí injekce
**MÍRNÁ BOLEST** Neslučitelná s mnoha aktivitami	Zneklidňující bolest	☐	Silná, hluboká bolest
	Velmi zneklidňující bolest	☐	Silná, hluboká, pronikavá bolest, např. vyvrknutý kotník
	Intenzivní bolest	☐	Ještě silnější bolest srovnatelná s bodnutím několika včelích žihadel
**TĚŽKÁ BOLEST** Nejste schopni fungovat	Velmi intenzivní bolest	☐	Bolest srovnatelná s průměrnou migrénou
	Naprosto hrozná bolest	☐	Bolest srovnatelná s porodem nebo opravdu silnou migrénou
	Nesnesitelná bolest	☐	Bolest nelze ignorovat, vyžaduje léky proti bolesti, nutné navštívit lékaře

Jak byste posoudil/a Vaši obvyklou míru zrakové pozornosti nebo výdrže při sledování běžného celovečerního filmu?

sleduji bez problémů / sleduji s přestávkami / nemůžu sledovat / nezajímá mne to (zakroužkováno: sleduji bez problémů)

Děkujeme Vám za účast v experimentu a při vyplnění dotazníku...

C5

Informace, dotazník a protokol experimentu pro
Bezkontaktní ovládání polohovacího lůžka

řešitel: Ing. Martin Kopeček, MEng, kopecema@lfhk.cuni.cz

Dotazník vyplňte co možná nejdříve po provedení testu.

Otázky pro instruktora

Experiment proveden dne: 7. 6. 2020

Jméno instruktora: Kopeček

Jméno pacienta: [illegible] Pohlaví: žena Věk: 50

Číslo logu aplikace:

Typ omezení: Paraparéza, Paraplegie, Kvadruplegie, Pentaplegie, Svalové dystrofie, ALS,

Jiné: zdravá

Popis hybnosti: plná

Brýle / kontaktní čočky: +1,5 D ano / ~~ne~~ (ne zakroužkováno)

Protokol experimentu:

1. Kalibrace senzoru ☑
2. Vysvětlení principu a umožnění získat zkušenost s ovládáním, alespoň 10 minut ☑
3. Uvedení aplikaci do počátečních podmínek – lůžko v bodě nula ☑

Čas při započetí testování: 10^{15}

4. **Test 1** ☑
   a) zahájit oční kontakt s aplikací
   b) **zvednout** podpěru nohou (po dobu 2-3 s)
   c) **zvednout** podpěru hlavy (po dobu 2-3 s)
   d) **snížit** podpěru nohou (po dobu 2-3 s)
   e) **snížit** podpěru hlavy (po dobu 2-3 s)
   f) přerušit oční kontakt s aplikací

7. **Test 2** – opakování **Testu 1** ☑
8. **Test 3** – opakování **Testu 1** ☑

Čas při ukončení testování: 10^{30}

Poznámky instruktora k experimentu:

C5

Otázky pro pacienta

Získal/a jste jistotu v ovládání? *ano / spíše ano/ nevím / spíše ne / ne*

Jak jste spokojen/a s ovládáním aplikace?

- aktivace ovládání — *velmi snadná / snadná / běžná / obtížná / velmi obtížná*
- způsob volby polohy — *velmi snadný / snadný / běžný / obtížný / velmi obtížný*
- informace o nastavení lůžka — *velmi snadné / snadné / běžné / obtížné / velmi obtížné*
- přehlednost ovládání — *velmi přehledné / přehledné/ běžné / nepřehledné/ velmi nepřehledné*

Upřednostnil/a byste ovládání jiným způsobem? *ano / spíše ano/ nevím / spíše ne / ne*

Pokud ano jakým (např. řeč)? ....

Máte nějaký poznatek k testu nebo ovládání?

Unavení oči

Jak byste hodnotil/a Vaši aktuální intenzitu bolesti na uvedené obrazové škále?

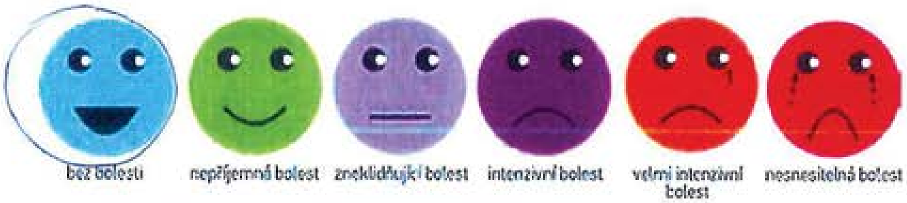

**MALÁ NEBO ŽÁDNÁ BOLEST** Jste schopni se bolesti přizpůsobit	Velmi mírná bolest	☐	Velmi lehká, sotva znatelná bolest
	Nepříjemná bolest	☐	Menší bolest, lze ji přirovnat k lehkému štípání
	Přijatelná bolest	☐	Velmi nápadná bolest podobající se píchnutí injekce
**MÍRNÁ BOLEST** Neslučitelná s mnoha aktivitami	Zneklidňující bolest	☐	Silná, hluboká bolest
	Velmi zneklidňující bolest	☐	Silná, hluboká, pronikavá bolest, např. vyvrknutý kotník
	Intenzivní bolest	☐	Ještě silnější bolest srovnatelná s bodnutím několika včelích žihadel
**TĚŽKÁ BOLEST** Nejste schopni fungovat	Velmi intenzivní bolest	☐	Bolest srovnatelná s průměrnou migrénou
	Naprosto hrozná bolest	☐	Bolest srovnatelná s porodem nebo opravdu silnou migrénou
	Nesnesitelná bolest	☐	Bolest nelze ignorovat, vyžaduje léky proti bolesti, nutné navštívit lékaře

Jak byste posoudil/a Vaši obvyklou míru zrakové pozornosti nebo výdrže při sledování běžného celovečerního filmu?

sleduji bez problémů / sleduji s přestávkami / nemůžu sledovat / nezajímá mne to

Děkujeme Vám za účast v experimentu a při vyplnění dotazníku...

C6

Informace, dotazník a protokol experimentu pro
Bezkontaktní ovládání polohovacího lůžka

řešitel: Ing. Martin Kopeček, MEng, kopecema@lfhk.cuni.cz

Dotazník vyplňte co možná nejdříve po provedení testu.

Otázky pro instruktora

Experiment proveden dne: 9. 6. 2020

Jméno instruktora: KOPEČEK

Jméno pacienta: Pohlaví: M Věk: 57

Číslo logu aplikace:

Typ omezení: Paraparéza, Paraplegie, Kvadruplegie, Pentaplegie, Svalové dystrofie, ALS,

Jiné: ŽÁDNÁ

Popis hybnosti: PLNÁ

Brýle / kontaktní čočky: ano / **ne**

Protokol experimentu:

1. Kalibrace senzoru ☑
2. Vysvětlení principu a umožnění získat zkušenost s ovládáním, alespoň 10 minut ☑
3. Uvedení aplikaci do počátečních podmínek – lůžko v bodě nula ☑

Čas při započetí testování: 10:30

4. **Test 1** ☑
   a) zahájit oční kontakt s aplikací
   b) **zvednout** podpěru nohou (po dobu 2-3 s)
   c) **zvednout** podpěru hlavy (po dobu 2-3 s)
   d) **snížit** podpěru nohou (po dobu 2-3 s)
   e) **snížit** podpěru hlavy (po dobu 2-3 s)
   f) přerušit oční kontakt s aplikací
7. **Test 2** – opakování **Testu 1** ☑
8. **Test 3** – opakování **Testu 1** ☑

Čas při ukončení testování: 11:00

Poznámky instruktora k experimentu:

20 C6

Otázky pro pacienta

Získal/a jste jistotu v ovládání? *ano / spíše ano/ nevím / spíše ne / ne*

Jak jste spokojen/a s ovládáním aplikace?

- aktivace ovládání *velmi snadná / snadná / běžná / obtížná / velmi obtížná*
- způsob volby polohy *velmi snadný / snadný / běžný / obtížný / velmi obtížný*
- informace o nastavení lůžka *velmi snadné / snadné / běžné / obtížné / velmi obtížné*
- přehlednost ovládání *velmi přehledné / přehledné/ běžné / nepřehledné/ velmi nepřehledné*

Upřednostnil/a byste ovládání jiným způsobem? *ano / spíše ano/ nevím / spíše ne / ne*

Pokud ano jakým (např. řeč)? ....

Máte nějaký poznatek k testu nebo ovládání?

ZMĚNA BARV ZELENÁ / BÍLÁ!

Jak byste hodnotil/a Vaši aktuální intenzitu bolesti na uvedené obrazové škále?

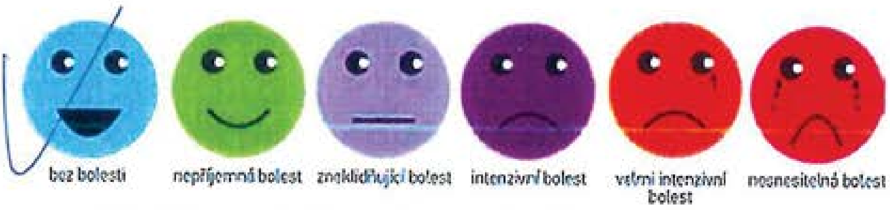

**MALÁ NEBO ŽÁDNÁ BOLEST** Jste schopni se bolesti přizpůsobit	Velmi mírná bolest	☐	Velmi lehká, sotva znatelná bolest
	Nepříjemná bolest	☐	Menší bolest, lze ji přirovnat k lehkému štípání
	Přijatelná bolest	☐	Velmi nápadná bolest podobající se píchnutí injekce
**MÍRNÁ BOLEST** Neslučitelná s mnoha aktivitami	Zneklidňující bolest	☐	Silná, hluboká bolest
	Velmi zneklidňující bolest	☐	Silná, hluboká, pronikavá bolest, např. vyvrknutý kotník
	Intenzivní bolest	☐	Ještě silnější bolest srovnatelná s bodnutím několika včelích žihadel
**TĚŽKÁ BOLEST** Nejste schopni fungovat	Velmi intenzivní bolest	☐	Bolest srovnatelná s průměrnou migrénou
	Naprosto hrozná bolest	☐	Bolest srovnatelná s porodem nebo opravdu silnou migrénou
	Nesnesitelná bolest	☐	Bolest nelze ignorovat, vyžaduje léky proti bolesti, nutné navštívit lékaře

Jak byste posoudil/a Vaši obvyklou míru zrakové pozornosti nebo výdrže při sledování běžného celovečerního filmu?

sleduji bez problémů / sleduji s přestávkami / nemůžu sledovat / nezajímá mne to

Děkujeme Vám za účast v experimentu a při vyplnění dotazníku...

F.O. C7

Informace, dotazník a protokol experimentu pro

Bezkontaktní ovládání polohovacího lůžka

řešitel: Ing. Martin Kopeček, MEng, kopecema@lfhk.cuni.cz

Dotazník vyplňte co možná nejdříve po provedení testu.

Otázky pro instruktora

Experiment proveden dne: 9. 6. 2020

Jméno instruktora: [illegible]

Jméno pacienta: Pohlaví: Ž Věk: 49

Číslo logu aplikace:

Typ omezení: Paraparéza, Paraplegie, Kvadruplegie, Pentaplegie, Svalové dystrofie, ALS,

Jiné: žádné

Popis hybnosti: plná

Brýle / kontaktní čočky: +1,5 D bez brýlí ano / ~~ne~~ (ne zakroužkováno)

Protokol experimentu:

1. Kalibrace senzoru ☑
2. Vysvětlení principu a umožnění získat zkušenost s ovládáním, alespoň 10 minut ☑
3. Uvedení aplikaci do počátečních podmínek – lůžko v bodě nula ☑

   Čas při započetí testování: 9:45

4. **Test 1** ☑
   a) zahájit oční kontakt s aplikací
   b) **zvednout** podpěru nohou (po dobu 2-3 s)
   c) **zvednout** podpěru hlavy (po dobu 2-3 s)
   d) **snížit** podpěru nohou (po dobu 2-3 s)
   e) **snížit** podpěru hlavy (po dobu 2-3 s)
   f) přerušit oční kontakt s aplikací

7. **Test 2** – opakování **Testu 1** ☑
8. **Test 3** – opakování **Testu 1** ☑

   Čas při ukončení testování: 10:00

Poznámky instruktora k experimentu:

C7

Otázky pro pacienta

Získal/a jste jistotu v ovládání? *ano / spíše ano / nevím / spíše ne / ne*

Jak jste spokojen/a s ovládáním aplikace?

- aktivace ovládání — *velmi snadná / snadná / běžná / obtížná / velmi obtížná*
- způsob volby polohy — *velmi snadný / snadný / běžný / obtížný / velmi obtížný*
- informace o nastavení lůžka — *velmi snadné / snadné / běžné / obtížné / velmi obtížné*
- přehlednost ovládání — *velmi přehledné / přehledné / běžné / nepřehledné / velmi nepřehledné*

Upřednostnil/a byste ovládání jiným způsobem? *ano / spíše ano / nevím / spíše ne / ne*

Pokud ano jakým (např. řeč)? ....

Máte nějaký poznatek k testu nebo ovládání?

Jak byste hodnotil/a Vaši aktuální intenzitu bolesti na uvedené obrazové škále?

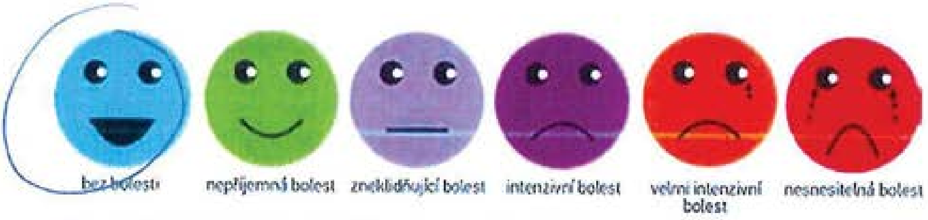

**MALÁ NEBO ŽÁDNÁ BOLEST** Jste schopni se bolesti přizpůsobit	Velmi mírná bolest	☐	Velmi lehká, sotva znatelná bolest
	Nepříjemná bolest	☐	Menší bolest, lze ji přirovnat k lehkému štípání
	Přijatelná bolest	☐	Velmi nápadná bolest podobající se píchnutí injekce
**MÍRNÁ BOLEST** Neslučitelná s mnoha aktivitami	Zneklidňující bolest	☐	Silná, hluboká bolest
	Velmi zneklidňující bolest	☐	Silná, hluboká, pronikavá bolest, např. vyvrknutý kotník
	Intenzivní bolest	☐	Ještě silnější bolest srovnatelná s bodnutím několika včelích žihadel
**TĚŽKÁ BOLEST** Nejste schopni fungovat	Velmi intenzivní bolest	☐	Bolest srovnatelná s průměrnou migrénou
	Naprosto hrozná bolest	☐	Bolest srovnatelná s porodem nebo opravdu silnou migrénou
	Nesnesitelná bolest	☐	Bolest nelze ignorovat, vyžaduje léky proti bolesti, nutné navštívit lékaře

Jak byste posoudil/a Vaši obvyklou míru zrakové pozornosti nebo výdrže při sledování běžného celovečerního filmu?

sleduji bez problémů / sleduji s přestávkami / nemůžu sledovat / nezajímá mne to

Děkujeme Vám za účast v experimentu a při vyplnění dotazníku...

80 C8

Informace, dotazník a protokol experimentu pro

Bezkontaktní ovládání polohovacího lůžka

řešitel: Ing. Martin Kopeček, MEng, kopecema@lfhk.cuni.cz

Dotazník vyplňte co možná nejdříve po provedení testu.

Otázky pro instruktora

Experiment proveden dne: 9.6.2020

Jméno instruktora: KOPŘIVA

Jméno pacienta: Pohlaví: M Věk: 65

Číslo logu aplikace:

Typ omezení: Paraparéza, Paraplegie, Kvadruplegie, Pentaplegie, Svalové dystrofie, ALS,

Jiné: ŽÁDNÉ

Popis hybnosti: PLNÁ

Brýle / kontaktní čočky: (ano) / ne

Protokol experimentu:

1. Kalibrace senzoru ☑
2. Vysvětlení principu a umožnění získat zkušenost s ovládáním, alespoň 10 minut ☑
3. Uvedení aplikaci do počátečních podmínek – lůžko v bodě nula ☑

   Čas při započetí testování: 9:15

4. **Test 1** ☑
   a) zahájit oční kontakt s aplikací
   b) **zvednout** podpěru nohou (po dobu 2-3 s)
   c) **zvednout** podpěru hlavy (po dobu 2-3 s)
   d) **snížit** podpěru nohou (po dobu 2-3 s)
   e) **snížit** podpěru hlavy (po dobu 2-3 s)
   f) přerušit oční kontakt s aplikací
7. **Test 2** – opakování **Testu 1** ☑
8. **Test 3** – opakování **Testu 1** ☑

   Čas při ukončení testování: 9:30

Poznámky instruktora k experimentu:

30 C8

Otázky pro pacienta

Získal/a jste jistotu v ovládání? *ano / spíše ano/ nevím / spíše ne / ne*

Jak jste spokojen/a s ovládáním aplikace?

aktivace ovládání *velmi snadná / snadná / běžná / obtížná / velmi obtížná*

způsob volby polohy *velmi snadný / snadný / běžný / obtížný / velmi obtížný*

informace o nastavení lůžka *velmi snadné / snadné / běžné / obtížné / velmi obtížné*

přehlednost ovládání *velmi přehledné / přehledné/ běžné / nepřehledné/ velmi nepřehledné*

Upřednostnil/a byste ovládání jiným způsobem? *ano / spíše ano/ nevím / spíše ne / ne*

Pokud ano jakým (např. řeč)? ....

Máte nějaký poznatek k testu nebo ovládání?

Funguje výborně.

Jak byste hodnotil/a Vaši aktuální intenzitu bolesti na uvedené obrazové škále?

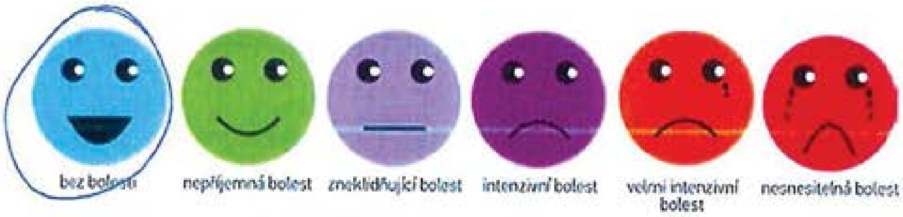

**MALÁ NEBO ŽÁDNÁ BOLEST** Jste schopni se bolesti přizpůsobit	Velmi mírná bolest	☐	Velmi lehká, sotva znatelná bolest
	Nepříjemná bolest	☐	Menší bolest, lze ji přirovnat k lehkému štípání
	Přijatelná bolest	☐	Velmi nápadná bolest podobající se píchnutí injekce
**MÍRNÁ BOLEST** Neslučitelná s mnoha aktivitami	Zneklidňující bolest	☐	Silná, hluboká bolest
	Velmi zneklidňující bolest	☐	Silná, hluboká, pronikavá bolest, např. vyvrknutý kotník
	Intenzivní bolest	☐	Ještě silnější bolest srovnatelná s bodnutím několika včelích žihadel
**TĚŽKÁ BOLEST** Nejste schopni fungovat	Velmi intenzivní bolest	☐	Bolest srovnatelná s průměrnou migrénou
	Naprosto hrozná bolest	☐	Bolest srovnatelná s porodem nebo opravdu silnou migrénou
	Nesnesitelná bolest	☐	Bolest nelze ignorovat, vyžaduje léky proti bolesti, nutné navštívit lékaře

Jak byste posoudil/a Vaši obvyklou míru zrakové pozornosti nebo výdrže při sledování běžného celovečerního filmu?

sleduji bez problémů / sleduji s přestávkami / nemůžu sledovat / nezajímá mne to

Děkujeme Vám za účast v experimentu a při vyplnění dotazníku...

P)

C9

Informace, dotazník a protokol experimentu pro
Bezkontaktní ovládání polohovacího lůžka

řešitel: Ing. Martin Kopeček, MEng, kopecema@lfhk.cuni.cz

Dotazník vyplňte co možná nejdříve po provedení testu.

Otázky pro instruktora

Experiment proveden dne: 8. 6. 2020

Jméno instruktora: KOPEČEK

Jméno pacienta: Pohlaví: M Věk: 34

Číslo logu aplikace:

Typ omezení: Paraparéza, Paraplegie, Kvadruplegie, Pentaplegie, Svalové dystrofie, ALS,

Jiné: ŽÁDNÉ

Popis hybnosti: PLNÁ

Brýle / kontaktní čočky: ano / **(ne)**

Protokol experimentu:

1. Kalibrace senzoru ☑
2. Vysvětlení principu a umožnění získat zkušenost s ovládáním, alespoň 10 minut ☑
3. Uvedení aplikaci do počátečních podmínek – lůžko v bodě nula ☑

Čas při započetí testování: 13:00

4. **Test 1** ☑
   a) zahájit oční kontakt s aplikací
   b) **zvednout** podpěru nohou (po dobu 2-3 s)
   c) **zvednout** podpěru hlavy (po dobu 2-3 s)
   d) **snížit** podpěru nohou (po dobu 2-3 s)
   e) **snížit** podpěru hlavy (po dobu 2-3 s)
   f) přerušit oční kontakt s aplikací
7. **Test 2** – opakování **Testu 1** ☑
8. **Test 3** – opakování **Testu 1** ☑

Čas při ukončení testování: 13:15

Poznámky instruktora k experimentu:

P? C9

Otázky pro pacienta

Získal/a jste jistotu v ovládání? *ano / spíše ano/ nevím / spíše ne / ne*

Jak jste spokojen/a s ovládáním aplikace?

aktivace ovládání *velmi snadná / snadná / běžná / obtížná / velmi obtížná*

způsob volby polohy *velmi snadný / snadný / běžný / obtížný / velmi obtížný*

informace o nastavení lůžka *velmi snadné / snadné / běžné / obtížné / velmi obtížné*

přehlednost ovládání *velmi přehledné / přehledné/ běžné / nepřehledné/ velmi nepřehledné*

Upřednostnil/a byste ovládání jiným způsobem? *ano / spíše ano/ nevím / spíše ne / ne*

Pokud ano jakým (např. řeč)? ....

Máte nějaký poznatek k testu nebo ovládání?

Jak byste hodnotil/a Vaši aktuální intenzitu bolesti na uvedené obrazové škále?

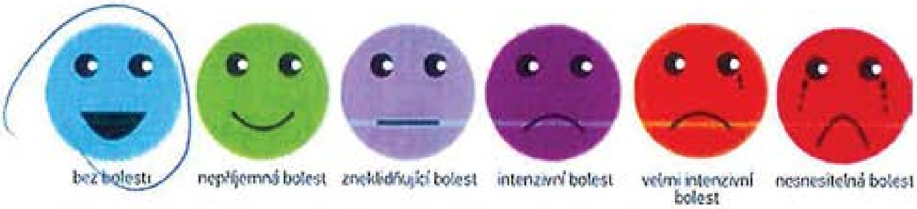

**MALÁ NEBO ŽÁDNÁ BOLEST** Jste schopni se bolesti přizpůsobit	Velmi mírná bolest	☐	Velmi lehká, sotva znatelná bolest
	Nepříjemná bolest	☐	Menší bolest, lze ji přirovnat k lehkému štípání
	Přijatelná bolest	☐	Velmi nápadná bolest podobající se píchnutí injekce
**MÍRNÁ BOLEST** Neslučitelná s mnoha aktivitami	Zneklidňující bolest	☐	Silná, hluboká bolest
	Velmi zneklidňující bolest	☐	Silná, hluboká, pronikavá bolest, např. vyvrknutý kotník
	Intenzivní bolest	☐	Ještě silnější bolest srovnatelná s bodnutím několika včelích žihadel
**TĚŽKÁ BOLEST** Nejste schopni fungovat	Velmi intenzivní bolest	☐	Bolest srovnatelná s průměrnou migrénou
	Naprosto hrozná bolest	☐	Bolest srovnatelná s porodem nebo opravdu silnou migrénou
	Nesnesitelná bolest	☐	Bolest nelze ignorovat, vyžaduje léky proti bolesti, nutné navštívit lékaře

Jak byste posoudil/a Vaši obvyklou míru zrakové pozornosti nebo výdrže při sledování běžného celovečerního filmu?

sleduji bez problémů / sleduji s přestávkami / nemůžu sledovat / nezajímá mne to

Děkujeme Vám za účast v experimentu a při vyplnění dotazníku...

C 10

Informace, dotazník a protokol experimentu pro

Bezkontaktní ovládání polohovacího lůžka

řešitel: Ing. Martin Kopeček, MEng, kopecema@lfhk.cuni.cz

Dotazník vyplňte co možná nejdříve po provedení testu.

Otázky pro instruktora

Experiment proveden dne: 8. 6. 2020

Jméno instruktora: KOTRČKĚK

Jméno pacienta: / Pohlaví: Ž Věk: 49

Číslo logu aplikace:

Typ omezení: Paraparéza, Paraplegie, Kvadruplegie, Pentaplegie, Svalové dystrofie, ALS,

Jiné: ŽÁDNÉ

Popis hybnosti: PLNÁ

Brýle / kontaktní čočky: ASTIGMAT. (ano) / ne

Protokol experimentu:

1. Kalibrace senzoru ☑
2. Vysvětlení principu a umožnění získat zkušenost s ovládáním, alespoň 10 minut ☑
3. Uvedení aplikaci do počátečních podmínek – lůžko v bodě nula ☑

Čas při započetí testování: 11:30

4. **Test 1** ☑
   a) zahájit oční kontakt s aplikací
   b) **zvednout** podpěru nohou (po dobu 2-3 s)
   c) **zvednout** podpěru hlavy (po dobu 2-3 s)
   d) **snížit** podpěru nohou (po dobu 2-3 s)
   e) **snížit** podpěru hlavy (po dobu 2-3 s)
   f) přerušit oční kontakt s aplikací
7. **Test 2** – opakování **Testu 1** ☑
8. **Test 3** – opakování **Testu 1** ☑

Čas při ukončení testování: 11:45

Poznámky instruktora k experimentu:

C10

Otázky pro pacienta

Získal/a jste jistotu v ovládání? ano / spíše ano/ nevím / spíše ne / ne

Jak jste spokojen/a s ovládáním aplikace?

aktivace ovládání velmi snadná / snadná / běžná / obtížná / velmi obtížná

způsob volby polohy velmi snadný / snadný / běžný / obtížný / velmi obtížný

informace o nastavení lůžka velmi snadné / snadné / běžné / obtížné / velmi obtížné

přehlednost ovládání *velmi přehledné / přehledné/ běžné / nepřehledné/ velmi nepřehledné*

Upřednostnil/a byste ovládání jiným způsobem? *ano / spíše ano/ nevím / spíše ne / ne*

Pokud ano jakým (např. řeč)? ....

Máte nějaký poznatek k testu nebo ovládání?

Jak byste hodnotil/a Vaši aktuální intenzitu bolesti na uvedené obrazové škále?

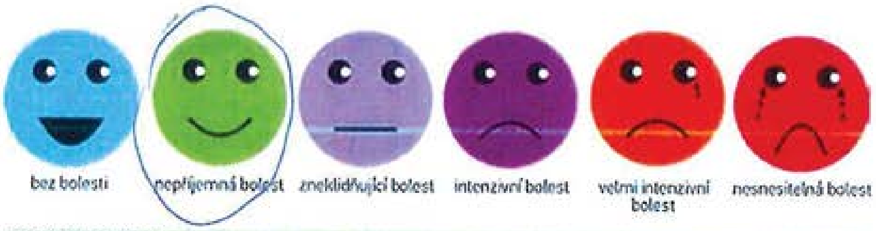

**MALÁ NEBO ŽÁDNÁ BOLEST** Jste schopni se bolesti přizpůsobit	Velmi mírná bolest	☐	Velmi lehká, sotva znatelná bolest
	Nepříjemná bolest	☐	Menší bolest, lze ji přirovnat k lehkému štípání
	Přijatelná bolest	☐	Velmi nápadná bolest podobající se píchnutí injekce
**MÍRNÁ BOLEST** Neslučitelná s mnoha aktivitami	Zneklidňující bolest	☐	Silná, hluboká bolest
	Velmi zneklidňující bolest	☐	Silná, hluboká, pronikavá bolest, např. vyvrknutý kotník
	Intenzivní bolest	☐	Ještě silnější bolest srovnatelná s bodnutím několika včelích žihadel
**TĚŽKÁ BOLEST** Nejste schopni fungovat	Velmi intenzivní bolest	☐	Bolest srovnatelná s průměrnou migrénou
	Naprosto hrozná bolest	☐	Bolest srovnatelná s porodem nebo opravdu silnou migrénou
	Nesnesitelná bolest	☐	Bolest nelze ignorovat, vyžaduje léky proti bolesti, nutné navštívit lékaře

Jak byste posoudil/a Vaši obvyklou míru zrakové pozornosti nebo výdrže při sledování běžného celovečerního filmu?

sleduji bez problémů / sleduji s přestávkami / nemůžu sledovat / nezajímá mne to

Děkujeme Vám za účast v experimentu a při vyplnění dotazníku...

M [illegible]

C11

Informace, dotazník a protokol experimentu pro
Bezkontaktní ovládání polohovacího lůžka

řešitel: Ing. Martin Kopeček, MEng, kopecema@lfhk.cuni.cz

Dotazník vyplňte co možná nejdříve po provedení testu.

Otázky pro instruktora

Experiment proveden dne: 5.6.2020

Jméno instruktora: KOPEČEK

Jméno pacienta: Pohlaví: Ž Věk: 48

Číslo logu aplikace:

Typ omezení: Paraparéza, Paraplegie, Kvadruplegie, Pentaplegie, Svalové dystrofie, ALS,

Jiné: ŽÁDNÉ

Popis hybnosti: PLNÁ

Brýle / kontaktní čočky: ano / (ne)

Protokol experimentu:

1. Kalibrace senzoru ☑
2. Vysvětlení principu a umožnění získat zkušenost s ovládáním, alespoň 10 minut ☑
3. Uvedení aplikaci do počátečních podmínek – lůžko v bodě nula ☑

   Čas při započetí testování: 10^{15}

4. **Test 1** ☑
   a) zahájit oční kontakt s aplikací
   b) **zvednout** podpěru nohou (po dobu 2-3 s)
   c) **zvednout** podpěru hlavy (po dobu 2-3 s)
   d) **snížit** podpěru nohou (po dobu 2-3 s)
   e) **snížit** podpěru hlavy (po dobu 2-3 s)
   f) přerušit oční kontakt s aplikací
7. **Test 2** – opakování **Testu 1** ☑
8. **Test 3** – opakování **Testu 1** ☑

   Čas při ukončení testování: 10^{30}

Poznámky instruktora k experimentu:

MO C11

Otázky pro pacienta

Získal/a jste jistotu v ovládání? — *ano / spíše ano / nevím / spíše ne / ne*

Jak jste spokojen/a s ovládáním aplikace?

- aktivace ovládání — *velmi snadná / snadná / běžná / obtížná / velmi obtížná*
- způsob volby polohy — *velmi snadný / snadný / běžný / obtížný / velmi obtížný*
- informace o nastavení lůžka — *velmi snadné / snadné / běžné / obtížné / velmi obtížné*
- přehlednost ovládání — *velmi přehledné / přehledné / běžné / nepřehledné / velmi nepřehledné*

Upřednostnil/a byste ovládání jiným způsobem? — *ano / spíše ano / nevím / spíše ne / ne*

Pokud ano jakým (např. řeč)? ....

Máte nějaký poznatek k testu nebo ovládání?

____

____

Jak byste hodnotil/a Vaši aktuální intenzitu bolesti na uvedené obrazové škále?

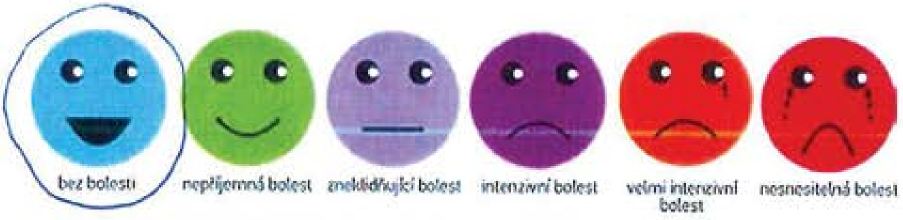

**MALÁ NEBO ŽÁDNÁ BOLEST** Jste schopni se bolesti přizpůsobit	Velmi mírná bolest	☐	Velmi lehká, sotva znatelná bolest
	Nepříjemná bolest	☐	Menší bolest, lze ji přirovnat k lehkému štípání
	Přijatelná bolest	☐	Velmi nápadná bolest podobající se píchnutí injekce
**MÍRNÁ BOLEST** Neslučitelná s mnoha aktivitami	Zneklidňující bolest	☐	Silná, hluboká bolest
	Velmi zneklidňující bolest	☐	Silná, hluboká, pronikavá bolest, např. vyvrknutý kotník
	Intenzivní bolest	☐	Ještě silnější bolest srovnatelná s bodnutím několika včelích žihadel
**TĚŽKÁ BOLEST** Nejste schopni fungovat	Velmi intenzivní bolest	☐	Bolest srovnatelná s průměrnou migrénou
	Naprosto hrozná bolest	☐	Bolest srovnatelná s porodem nebo opravdu silnou migrénou
	Nesnesitelná bolest	☐	Bolest nelze ignorovat, vyžaduje léky proti bolesti, nutné navštívit lékaře

Jak byste posoudil/a Vaši obvyklou míru zrakové pozornosti nebo výdrže při sledování běžného celovečerního filmu?

sleduji bez problémů / sleduji s přestávkami / nemůžu sledovat / nezajímá mne to

Děkujeme Vám za účast v experimentu a při vyplnění dotazníku...

C 12

Informace, dotazník a protokol experimentu pro
Bezkontaktní ovládání polohovacího lůžka

řešitel: Ing. Martin Kopeček, MEng, kopecema@lfhk.cuni.cz

Dotazník vyplňte co možná nejdříve po provedení testu.

Otázky pro instruktora

Experiment proveden dne: 5. 6. 2020

Jméno instruktora: [illegible]

Jméno pacienta: Pohlaví: M Věk: 66

Číslo logu aplikace:

Typ omezení: Paraparéza, Paraplegie, Kvadruplegie, Pentaplegie, Svalové dystrofie, ALS,

Jiné: ŽÁDNÉ

Popis hybnosti: PLNÁ

Brýle / kontaktní čočky: (ano) / ne

Protokol experimentu:

1. Kalibrace senzoru ☐
2. Vysvětlení principu a umožnění získat zkušenost s ovládáním, alespoň 10 minut ☐
3. Uvedení aplikaci do počátečních podmínek – lůžko v bodě nula ☐

Čas při započetí testování: ....

4. **Test 1** ☐
   a) zahájit oční kontakt s aplikací
   b) **zvednout** podpěru nohou (po dobu 2-3 s)
   c) **zvednout** podpěru hlavy (po dobu 2-3 s)
   d) **snížit** podpěru nohou (po dobu 2-3 s)
   e) **snížit** podpěru hlavy (po dobu 2-3 s)
   f) přerušit oční kontakt s aplikací
7. **Test 2** – opakování **Testu 1** ☐
8. **Test 3** – opakování **Testu 1** ☐

Čas při ukončení testování: ....

Poznámky instruktora k experimentu:

C12

Otázky pro pacienta

Získal/a jste jistotu v ovládání? ano / spíše ano/ nevím / spíše ne / ne

Jak jste spokojen/a s ovládáním aplikace?

- aktivace ovládání — *velmi snadná / snadná / běžná / obtížná / velmi obtížná*
- způsob volby polohy — *velmi snadný / snadný / běžný / obtížný / velmi obtížný*
- informace o nastavení lůžka — *velmi snadné / snadné / běžné / obtížné / velmi obtížné*
- přehlednost ovládání — *velmi přehledné / přehledné/ běžné / nepřehledné/ velmi nepřehledné*

Upřednostnil/a byste ovládání jiným způsobem? *ano / spíše ano/ nevím / spíše ne / ne*

Pokud ano jakým (např. řeč)? ....

Máte nějaký poznatek k testu nebo ovládání?

____

____

Jak byste hodnotil/a Vaši aktuální intenzitu bolesti na uvedené obrazové škále?

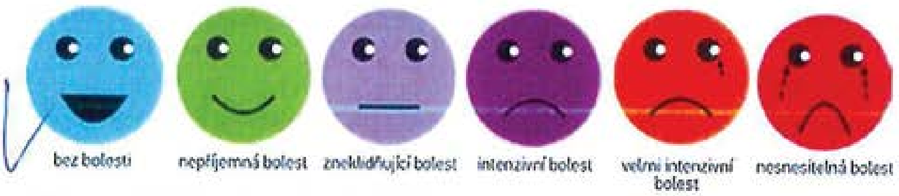

**MALÁ NEBO ŽÁDNÁ BOLEST** Jste schopni se bolesti přizpůsobit	Velmi mírná bolest	☐	Velmi lehká, sotva znatelná bolest
	Nepříjemná bolest	☐	Menší bolest, lze ji přirovnat k lehkému štípání
	Přijatelná bolest	☐	Velmi nápadná bolest podobající se píchnutí injekce
**MÍRNÁ BOLEST** Neslučitelná s mnoha aktivitami	Zneklidňující bolest	☐	Silná, hluboká bolest
	Velmi zneklidňující bolest	☐	Silná, hluboká, pronikavá bolest, např. vyvrknutý kotník
	Intenzivní bolest	☐	Ještě silnější bolest srovnatelná s bodnutím několika včelích žihadel
**TĚŽKÁ BOLEST** Nejste schopni fungovat	Velmi intenzivní bolest	☐	Bolest srovnatelná s průměrnou migrénou
	Naprosto hrozná bolest	☐	Bolest srovnatelná s porodem nebo opravdu silnou migrénou
	Nesnesitelná bolest	☐	Bolest nelze ignorovat, vyžaduje léky proti bolesti, nutné navštívit lékaře

Jak byste posoudil/a Vaši obvyklou míru zrakové pozornosti nebo výdrže při sledování běžného celovečerního filmu?

sleduji bez problémů / sleduji s přestávkami / nemůžu sledovat / nezajímá mne to

Děkujeme Vám za účast v experimentu a při vyplnění dotazníku...

C 13

Informace, dotazník a protokol experimentu pro

Bezkontaktní ovládání polohovacího lůžka

řešitel: Ing. Martin Kopeček, MEng, kopecema@lfhk.cuni.cz

Dotazník vyplňte co možná nejdříve po provedení testu.

Otázky pro instruktora

Experiment proveden dne: 5.6.2020

Jméno instruktora: KOPEČEK

Jméno pacienta: Pohlaví: Ž Věk: 50 l

Číslo logu aplikace:

Typ omezení: Paraparéza, Paraplegie, Kvadruplegie, Pentaplegie, Svalové dystrofie, ALS,

Jiné: ZÁDA

Popis hybnosti: PLNÁ

Brýle / kontaktní čočky: ano / ne (zakroužkováno: ne)

Protokol experimentu:

1. Kalibrace senzoru ☑
2. Vysvětlení principu a umožnění získat zkušenost s ovládáním, alespoň 10 minut ☑
3. Uvedení aplikaci do počátečních podmínek – lůžko v bodě nula ☑

   Čas při započetí testování: 9 15

4. **Test 1** ☑
   a) zahájit oční kontakt s aplikací
   b) **zvednout** podpěru nohou (po dobu 2-3 s)
   c) **zvednout** podpěru hlavy (po dobu 2-3 s)
   d) **snížit** podpěru nohou (po dobu 2-3 s)
   e) **snížit** podpěru hlavy (po dobu 2-3 s)
   f) přerušit oční kontakt s aplikací

7. **Test 2** – opakování **Testu 1** ☑
8. **Test 3** – opakování **Testu 1** ☑

   Čas při ukončení testování: 9 30

Poznámky instruktora k experimentu:

TEST 2 ŠPATNĚ NAVEDENÁ

C13

Otázky pro pacienta

Získal/a jste jistotu v ovládání? *ano / spíše ano / nevím / spíše ne / ne*

Jak jste spokojen/a s ovládáním aplikace?

- aktivace ovládání — *velmi snadná / snadná / běžná / obtížná / velmi obtížná*
- způsob volby polohy — *velmi snadný / snadný / běžný / obtížný / velmi obtížný*
- informace o nastavení lůžka — *velmi snadné / snadné / běžné / obtížné / velmi obtížné*
- přehlednost ovládání — *velmi přehledné / přehledné / běžné / nepřehledné / velmi nepřehledné*

Upřednostnil/a byste ovládání jiným způsobem? *ano / spíše ano / nevím / spíše ne / ne*

Pokud ano jakým (např. řeč)? ....

Máte nějaký poznatek k testu nebo ovládání?

____

____

Jak byste hodnotil/a Vaši aktuální intenzitu bolesti na uvedené obrazové škále?

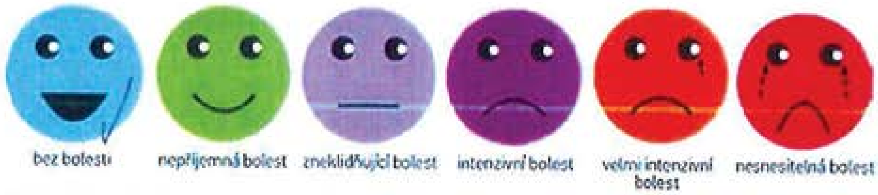

**MALÁ NEBO ŽÁDNÁ BOLEST** Jste schopni se bolesti přizpůsobit	Velmi mírná bolest	☐	Velmi lehká, sotva znatelná bolest
	Nepříjemná bolest	☐	Menší bolest, lze ji přirovnat k lehkému štípání
	Přijatelná bolest	☐	Velmi nápadná bolest podobající se píchnutí injekce
**MÍRNÁ BOLEST** Neslučitelná s mnoha aktivitami	Zneklidňující bolest	☐	Silná, hluboká bolest
	Velmi zneklidňující bolest	☐	Silná, hluboká, pronikavá bolest, např. vyvrknutý kotník
	Intenzivní bolest	☐	Ještě silnější bolest srovnatelná s bodnutím několika včelích žihadel
**TĚŽKÁ BOLEST** Nejste schopni fungovat	Velmi intenzivní bolest	☐	Bolest srovnatelná s průměrnou migrénou
	Naprosto hrozná bolest	☐	Bolest srovnatelná s porodem nebo opravdu silnou migrénou
	Nesnesitelná bolest	☐	Bolest nelze ignorovat, vyžaduje léky proti bolesti, nutné navštívit lékaře

Jak byste posoudil/a Vaši obvyklou míru zrakové pozornosti nebo výdrže při sledování běžného celovečerního filmu?

sleduji bez problémů / sleduji s přestávkami / nemůžu sledovat / nezajímá mne to

Děkujeme Vám za účast v experimentu a při vyplnění dotazníku...

[illegible] C 14

Informace, dotazník a protokol experimentu pro
Bezkontaktní ovládání polohovacího lůžka

řešitel: Ing. Martin Kopeček, MEng, kopecema@lfhk.cuni.cz

Dotazník vyplňte co možná nejdříve po provedení testu.

Otázky pro instruktora

Experiment proveden dne: 4. 6. 2020

Jméno instruktora: KOTRČÍK M.

Jméno pacienta: [illegible] ohlaví: Ž Věk: 34

Číslo logu aplikace:

Typ omezení: Paraparéza, Paraplegie, Kvadruplegie, Pentaplegie, Svalové dystrofie, ALS,

Jiné: ŽÁDNÁ

Popis hybnosti: PLNÁ

Brýle / kontaktní čočky: (ano) / ne

Protokol experimentu:

1. Kalibrace senzoru ☑
2. Vysvětlení principu a umožnění získat zkušenost s ovládáním, alespoň 10 minut ☑
3. Uvedení aplikaci do počátečních podmínek – lůžko v bodě nula ☑

Čas při započetí testování: 15:00 ☑

4. **Test 1**
   a) zahájit oční kontakt s aplikací
   b) **zvednout** podpěru nohou (po dobu 2-3 s)
   c) **zvednout** podpěru hlavy (po dobu 2-3 s)
   d) **snížit** podpěru nohou (po dobu 2-3 s)
   e) **snížit** podpěru hlavy (po dobu 2-3 s)
   f) přerušit oční kontakt s aplikací

7. **Test 2** – opakování **Testu 1** ☑
8. **Test 3** – opakování **Testu 1** ☑

Čas při ukončení testování: 15:15

Poznámky instruktora k experimentu:

C14

Otázky pro pacienta

Získal/a jste jistotu v ovládání? *ano / spíše ano/ nevím / spíše ne / ne*

Jak jste spokojen/a s ovládáním aplikace?

- aktivace ovládání — *velmi snadná / snadná / běžná / obtížná / velmi obtížná*
- způsob volby polohy — *velmi snadný / snadný / běžný / obtížný / velmi obtížný*
- informace o nastavení lůžka — *velmi snadné / snadné / běžné / obtížné / velmi obtížné*
- přehlednost ovládání — *velmi přehledné / přehledné/ běžné / nepřehledné/ velmi nepřehledné*

Upřednostnil/a byste ovládání jiným způsobem? *ano / spíše ano/ nevím / spíše ne / ne*

Pokud ano jakým (např. řeč)? ....

Máte nějaký poznatek k testu nebo ovládání?

Jak byste hodnotil/a Vaši aktuální intenzitu bolesti na uvedené obrazové škále?

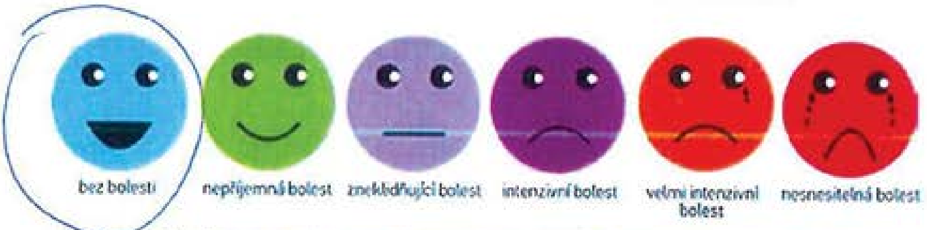

**MALÁ NEBO ŽÁDNÁ BOLEST** Jste schopni se bolesti přizpůsobit	Velmi mírná bolest	☐	Velmi lehká, sotva znatelná bolest
	Nepříjemná bolest	☐	Menší bolest, lze ji přirovnat k lehkému štípání
	Přijatelná bolest	☐	Velmi nápadná bolest podobající se píchnutí injekce
**MÍRNÁ BOLEST** Neslučitelná s mnoha aktivitami	Zneklidňující bolest	☐	Silná, hluboká bolest
	Velmi zneklidňující bolest	☐	Silná, hluboká, pronikavá bolest, např. vyvrknutý kotník
	Intenzivní bolest	☐	Ještě silnější bolest srovnatelná s bodnutím několika včelích žihadel
**TĚŽKÁ BOLEST** Nejste schopni fungovat	Velmi intenzivní bolest	☐	Bolest srovnatelná s průměrnou migrénou
	Naprosto hrozná bolest	☐	Bolest srovnatelná s porodem nebo opravdu silnou migrénou
	Nesnesitelná bolest	☐	Bolest nelze ignorovat, vyžaduje léky proti bolesti, nutné navštívit lékaře

Jak byste posoudil/a Vaši obvyklou míru zrakové pozornosti nebo výdrže při sledování běžného celovečerního filmu?

sleduji bez problémů / sleduji s přestávkami / nemůžu sledovat / nezajímá mne to

Děkujeme Vám za účast v experimentu a při vyplnění dotazníku...

C15

Informace, dotazník a protokol experimentu pro
Bezkontaktní ovládání polohovacího lůžka

řešitel: Ing. Martin Kopeček, MEng, kopecema@lfhk.cuni.cz

Dotazník vyplňte co možná nejdříve po provedení testu.

Otázky pro instruktora

Experiment proveden dne: 28.5.2020

Jméno instruktora: KoPRdza

Jméno pacienta:

Pohlaví: Ž

Věk: 24

Číslo logu aplikace:

Typ omezení: Paraparéza, Paraplegie, Kvadruplegie, Pentaplegie, Svalové dystrofie, ALS,

Jiné: ŽÁDNÉ

Popis hybnosti: PLNÁ

Brýle / kontaktní čočky: ano / ne ?

Protokol experimentu:

1. Kalibrace senzoru
2. Vysvětlení principu a umožnění získat zkušenost s ovládáním, alespoň 10 minut
3. Uvedení aplikaci do počátečních podmínek – lůžko v bodě nula

Čas při započetí testování: 8:15

4. **Test 1**
   a) zahájit oční kontakt s aplikací
   b) **zvednout** podpěru nohou (po dobu 2-3 s)
   c) **zvednout** podpěru hlavy (po dobu 2-3 s)
   d) **snížit** podpěru nohou (po dobu 2-3 s)
   e) **snížit** podpěru hlavy (po dobu 2-3 s)
   f) přerušit oční kontakt s aplikací
7. **Test 2** – opakování **Testu 1**
8. **Test 3** – opakování **Testu 1**

Čas při ukončení testování: 8:30

Poznámky instruktora k experimentu:

C15

Otázky pro pacienta

Získal/a jste jistotu v ovládání? *ano / spíše ano/ nevím / spíše ne / ne*

Jak jste spokojen/a s ovládáním aplikace?

- aktivace ovládání *velmi snadná / snadná / běžná / obtížná / velmi obtížná*
- způsob volby polohy *velmi snadný / snadný / běžný / obtížný / velmi obtížný*
- informace o nastavení lůžka *velmi snadné / snadné / běžné / obtížné / velmi obtížné*
- přehlednost ovládání *velmi přehledné / přehledné/ běžné / nepřehledné/ velmi nepřehledné*

Upřednostnil/a byste ovládání jiným způsobem? *ano / spíše ano/ nevím / spíše ne / ne*

Pokud ano jakým (např. řeč)? ....

Máte nějaký poznatek k testu nebo ovládání?

____

____

Jak byste hodnotil/a Vaši aktuální intenzitu bolesti na uvedené obrazové škále?

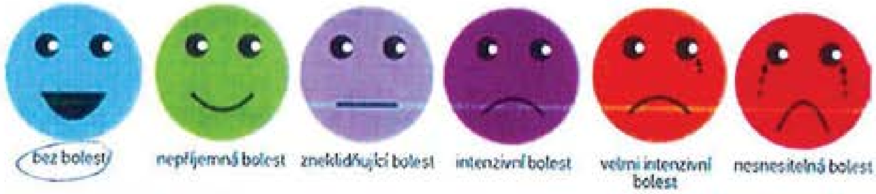

**MALÁ NEBO ŽÁDNÁ BOLEST** Jste schopni se bolesti přizpůsobit	Velmi mírná bolest	☐	Velmi lehká, sotva znatelná bolest
	Nepříjemná bolest	☐	Menší bolest, lze ji přirovnat k lehkému štípání
	Přijatelná bolest	☐	Velmi nápadná bolest podobající se píchnutí injekce
**MÍRNÁ BOLEST** Neslučitelná s mnoha aktivitami	Zneklidňující bolest	☐	Silná, hluboká bolest
	Velmi zneklidňující bolest	☐	Silná, hluboká, pronikavá bolest, např. vyvrknutý kotník
	Intenzivní bolest	☐	Ještě silnější bolest srovnatelná s bodnutím několika včelích žihadel
**TĚŽKÁ BOLEST** Nejste schopni fungovat	Velmi intenzivní bolest	☐	Bolest srovnatelná s průměrnou migrénou
	Naprosto hrozná bolest	☐	Bolest srovnatelná s porodem nebo opravdu silnou migrénou
	Nesnesitelná bolest	☐	Bolest nelze ignorovat, vyžaduje léky proti bolesti, nutné navštívit lékaře

Jak byste posoudil/a Vaši obvyklou míru zrakové pozornosti nebo výdrže při sledování běžného celovečerního filmu?

sleduji bez problémů / sleduji s přestávkami / nemůžu sledovat / nezajímá mne to

Děkujeme Vám za účast v experimentu a při vyplnění dotazníku...

C 16

Informace, dotazník a protokol experimentu pro
Bezkontaktní ovládání polohovacího lůžka
řešitel: Ing. Martin Kopeček, MEng, kopecema@lfhk.cuni.cz

Dotazník vyplňte co možná nejdříve po provedení testu.

Otázky pro instruktora

Experiment proveden dne: 13.5.2020

Jméno instruktora: Kozička

Jméno pacienta: Pohlaví: Ž Věk: 56

Číslo logu aplikace:

Typ omezení: Paraparéza, Paraplegie, Kvadruplegie, Pentaplegie, Svalové dystrofie, ALS,

Jiné: žádné

Popis hybnosti: plná

Brýle / kontaktní čočky: ano / ne (zakroužkováno: ne)

Protokol experimentu:

1. Kalibrace senzoru ☑
2. Vysvětlení principu a umožnění získat zkušenost s ovládáním, alespoň 10 minut ☑
3. Uvedení aplikaci do počátečních podmínek – lůžko v bodě nula ☑

Čas při započetí testování: 10:00

4. **Test 1** ☑
   a) zahájit oční kontakt s aplikací
   b) **zvednout** podpěru nohou (po dobu 2-3 s)
   c) **zvednout** podpěru hlavy (po dobu 2-3 s)
   d) **snížit** podpěru nohou (po dobu 2-3 s)
   e) **snížit** podpěru hlavy (po dobu 2-3 s)
   f) přerušit oční kontakt s aplikací
7. **Test 2** – opakování **Testu 1** ☑
8. **Test 3** – opakování **Testu 1** ☑

Čas při ukončení testování: 10:30

Poznámky instruktora k experimentu:

C 16

Otázky pro pacienta

Získal/a jste jistotu v ovládání? ano / spíše ano / nevím / spíše ne / ne

Jak jste spokojen/a s ovládáním aplikace?

aktivace ovládání velmi snadná / snadná / běžná / obtížná / velmi obtížná

způsob volby polohy velmi snadný / snadný / běžný / obtížný / velmi obtížný

informace o nastavení lůžka velmi snadné / snadné / běžné / obtížné / velmi obtížné

přehlednost ovládání velmi přehledné / přehledné/ běžné / nepřehledné/ velmi nepřehledné

Upřednostnil/a byste ovládání jiným způsobem? ano / spíše ano/ nevím / spíše ne / ne

Pokud ano jakým (např. řeč)? ....

Máte nějaký poznatek k testu nebo ovládání?

____

____

Jak byste hodnotil/a Vaši aktuální intenzitu bolesti na uvedené obrazové škále?

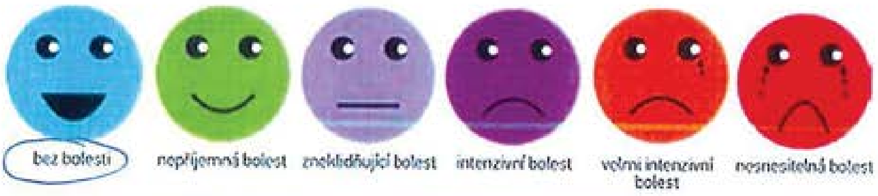

**MALÁ NEBO ŽÁDNÁ BOLEST** Jste schopni se bolesti přizpůsobit	Velmi mírná bolest	☐	Velmi lehká, sotva znatelná bolest
	Nepříjemná bolest	☐	Menší bolest, lze ji přirovnat k lehkému štípání
	Přijatelná bolest	☐	Velmi nápadná bolest podobající se píchnutí injekce
**MÍRNÁ BOLEST** Neslučitelná s mnoha aktivitami	Zneklidňující bolest	☐	Silná, hluboká bolest
	Velmi zneklidňující bolest	☐	Silná, hluboká, pronikavá bolest, např. vyvrknutý kotník
	Intenzivní bolest	☐	Ještě silnější bolest srovnatelná s bodnutím několika včelích žihadel
**TĚŽKÁ BOLEST** Nejste schopni fungovat	Velmi intenzivní bolest	☐	Bolest srovnatelná s průměrnou migrénou
	Naprosto hrozná bolest	☐	Bolest srovnatelná s porodem nebo opravdu silnou migrénou
	Nesnesitelná bolest	☐	Bolest nelze ignorovat, vyžaduje léky proti bolesti, nutné navštívit lékaře

Jak byste posoudil/a Vaši obvyklou míru zrakové pozornosti nebo výdrže při sledování běžného celovečerního filmu?

sleduji bez problémů / sleduji s přestávkami / nemůžu sledovat / nezajímá mne to

Děkujeme Vám za účast v experimentu a při vyplnění dotazníku...

F1

C17

Informace, dotazník a protokol experimentu pro
Bezkontaktní ovládání polohovacího lůžka

řešitel: Ing. Martin Kopeček, MEng, kopecema@lfhk.cuni.cz

Dotazník vyplňte co možná nejdříve po provedení testu.

Otázky pro instruktora

Experiment proveden dne:

Jméno instruktora:

Jméno pacienta: Pohlaví: Ž Věk: 38

Číslo logu aplikace:

Typ omezení: Paraparéza, Paraplegie, Kvadruplegie, Pentaplegie, Svalové dystrofie, ALS,

Jiné: [illegible]

Popis hybnosti: [illegible]

Brýle / kontaktní čočky: ano / ~~ne~~ (ne zakroužkováno)

Protokol experimentu:

1. Kalibrace senzoru ☑
2. Vysvětlení principu a umožnění získat zkušenost s ovládáním, alespoň 10 minut ☑
3. Uvedení aplikaci do počátečních podmínek – lůžko v bodě nula ☑

Čas při započetí testování: 14³⁰

4. **Test 1** ☑
   a) zahájit oční kontakt s aplikací
   b) **zvednout** podpěru nohou (po dobu 2-3 s)
   c) **zvednout** podpěru hlavy (po dobu 2-3 s)
   d) **snížit** podpěru nohou (po dobu 2-3 s)
   e) **snížit** podpěru hlavy (po dobu 2-3 s)
   f) přerušit oční kontakt s aplikací

7. **Test 2** – opakování **Testu 1** ☑
8. **Test 3** – opakování **Testu 1** ☑

Čas při ukončení testování: 14⁴⁵

Poznámky instruktora k experimentu:

C17

Otázky pro pacienta

Získal/a jste jistotu v ovládání? *ano / spíše ano / nevím / spíše ne / ne*

Jak jste spokojen/a s ovládáním aplikace?

- aktivace ovládání *velmi snadná / snadná / běžná / obtížná / velmi obtížná*
- způsob volby polohy *velmi snadný / snadný / běžný / obtížný / velmi obtížný*
- informace o nastavení lůžka *velmi snadné / snadné / běžné / obtížné / velmi obtížné*
- přehlednost ovládání *velmi přehledné / přehledné/ běžné / nepřehledné/ velmi nepřehledné*

Upřednostnil/a byste ovládání jiným způsobem? *ano / spíše ano/ nevím / spíše ne / ne*

Pokud ano jakým (např. řeč)? ....

Máte nějaký poznatek k testu nebo ovládání?

____

____

Jak byste hodnotil/a Vaši aktuální intenzitu bolesti na uvedené obrazové škále?

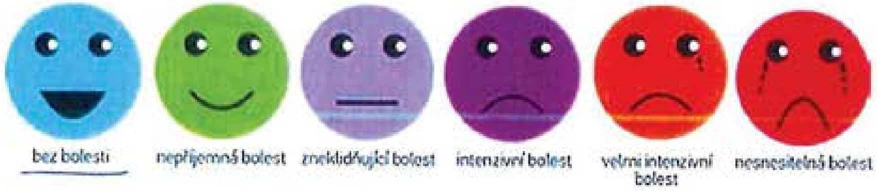

**MALÁ NEBO ŽÁDNÁ BOLEST**<br>Jste schopni se bolesti přizpůsobit	Velmi mírná bolest	☐	Velmi lehká, sotva znatelná bolest
	Nepříjemná bolest	☐	Menší bolest, lze ji přirovnat k lehkému štípání
	Přijatelná bolest	☐	Velmi nápadná bolest podobající se píchnutí injekce
**MÍRNÁ BOLEST**<br>Neslučitelná s mnoha aktivitami	Zneklidňující bolest	☐	Silná, hluboká bolest
	Velmi zneklidňující bolest	☐	Silná, hluboká, pronikavá bolest, např. vyvrknutý kotník
	Intenzivní bolest	☐	Ještě silnější bolest srovnatelná s bodnutím několika včelích žihadel
**TĚŽKÁ BOLEST**<br>Nejste schopni fungovat	Velmi intenzivní bolest	☐	Bolest srovnatelná s průměrnou migrénou
	Naprosto hrozná bolest	☐	Bolest srovnatelná s porodem nebo opravdu silnou migrénou
	Nesnesitelná bolest	☐	Bolest nelze ignorovat, vyžaduje léky proti bolesti, nutné navštívit lékaře

Jak byste posoudil/a Vaši obvyklou míru zrakové pozornosti nebo výdrže při sledování běžného celovečerního filmu?

sleduji bez problémů / sleduji s přestávkami / nemůžu sledovat / nezajímá mne to

Děkujeme Vám za účast v experimentu a při vyplnění dotazníku...

SA 0

C 18

Informace, dotazník a protokol experimentu pro

Bezkontaktní ovládání polohovacího lůžka

řešitel: Ing. Martin Kopeček, MEng, kopecema@lfhk.cuni.cz

Dotazník vyplňte co možná nejdříve po provedení testu.

Otázky pro instruktora

Experiment proveden dne: 27. 5. 2020

Jméno instruktora: [illegible]

Jméno pacienta: Pohlaví: Ž Věk: 22

Číslo logu aplikace:

Typ omezení: Paraparéza, Paraplegie, Kvadruplegie, Pentaplegie, Svalové dystrofie, ALS,

Jiné: žádné

Popis hybnosti: plná

Brýle / kontaktní čočky: ano / ne (ne zakroužkováno)

Protokol experimentu:

1. Kalibrace senzoru ☑
2. Vysvětlení principu a umožnění získat zkušenost s ovládáním, alespoň 10 minut ☑
3. Uvedení aplikaci do počátečních podmínek – lůžko v bodě nula ☑

Čas při započetí testování: 14:00 ☑

4. **Test 1** ☑
    a) zahájit oční kontakt s aplikací
    b) **zvednout** podpěru nohou (po dobu 2-3 s)
    c) **zvednout** podpěru hlavy (po dobu 2-3 s)
    d) **snížit** podpěru nohou (po dobu 2-3 s)
    e) **snížit** podpěru hlavy (po dobu 2-3 s)
    f) přerušit oční kontakt s aplikací
7. **Test 2** – opakování **Testu 1** ☑
8. **Test 3** – opakování **Testu 1** ☑

Čas při ukončení testování: 14:15

Poznámky instruktora k experimentu:

3).

C 18

Otázky pro pacienta

Získal/a jste jistotu v ovládání? — ano / spíše ano/ nevím / spíše ne / ne

Jak jste spokojen/a s ovládáním aplikace?

- aktivace ovládání — *velmi snadná / snadná / běžná / obtížná / velmi obtížná*
- způsob volby polohy — *velmi snadný / snadný / běžný / obtížný / velmi obtížný*
- informace o nastavení lůžka — *velmi snadné / snadné / běžné / obtížné / velmi obtížné*
- přehlednost ovládání — *velmi přehledné / přehledné/ běžné / nepřehledné/ velmi nepřehledné*

Upřednostnil/a byste ovládání jiným způsobem? — *ano / spíše ano/ nevím / spíše ne / ne*

Pokud ano jakým (např. řeč)? ....

Máte nějaký poznatek k testu nebo ovládání?

- jednou či dvakrát se mi stalo, že jsem byla asi trochu rychlejší v přechodu do další fáze (ze zvolení polohy po kontakt s bílou tečkou) a vyskočilo mi to - znovu jsem musela zvolit polohu

Jak byste hodnotil/a Vaši aktuální intenzitu bolesti na uvedené obrazové škále?

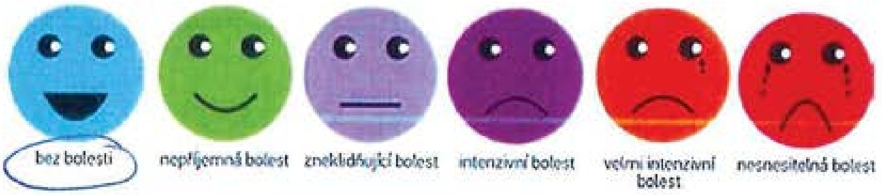

**MALÁ NEBO ŽÁDNÁ BOLEST** Jste schopni se bolesti přizpůsobit	Velmi mírná bolest	☐	Velmi lehká, sotva znatelná bolest
	Nepříjemná bolest	☐	Menší bolest, lze ji přirovnat k lehkému štípání
	Přijatelná bolest	☐	Velmi nápadná bolest podobající se píchnutí injekce
**MÍRNÁ BOLEST** Neslučitelná s mnoha aktivitami	Zneklidňující bolest	☐	Silná, hluboká bolest
	Velmi zneklidňující bolest	☐	Silná, hluboká, pronikavá bolest, např. vyvrknutý kotník
	Intenzivní bolest	☐	Ještě silnější bolest srovnatelná s bodnutím několika včelích žihadel
**TĚŽKÁ BOLEST** Nejste schopni fungovat	Velmi intenzivní bolest	☐	Bolest srovnatelná s průměrnou migrénou
	Naprosto hrozná bolest	☐	Bolest srovnatelná s porodem nebo opravdu silnou migrénou
	Nesnesitelná bolest	☐	Bolest nelze ignorovat, vyžaduje léky proti bolesti, nutné navštívit lékaře

Jak byste posoudil/a Vaši obvyklou míru zrakové pozornosti nebo výdrže při sledování běžného celovečerního filmu?

sleduji bez problémů / sleduji s přestávkami / nemůžu sledovat / nezajímá mne to

Děkujeme Vám za účast v experimentu a při vyplnění dotazníku...

P1

C19

Informace, dotazník a protokol experimentu pro
Bezkontaktní ovládání polohovacího lůžka

řešitel: Ing. Martin Kopeček, MEng, kopecema@lfhk.cuni.cz

Dotazník vyplňte co možná nejdříve po provedení testu.

Otázky pro instruktora

Experiment proveden dne: 20. 5. 2020

Jméno instruktora: KOPEČEK

Jméno pacienta: Pohlaví: Ž Věk: 29

Číslo logu aplikace:

Typ omezení: Paraparéza, Paraplegie, Kvadruplegie, Pentaplegie, Svalové dystrofie, ALS,

Jiné: ŽÁDNÉ

Popis hybnosti: PLNÁ

Brýle / kontaktní čočky: (ano) / ne

Protokol experimentu:

1. Kalibrace senzoru ☑
2. Vysvětlení principu a umožnění získat zkušenost s ovládáním, alespoň 10 minut ☑
3. Uvedení aplikaci do počátečních podmínek – lůžko v bodě nula ☑

Čas při započetí testování: 8:15

4. **Test 1** ☑
   a) zahájit oční kontakt s aplikací
   b) **zvednout** podpěru nohou (po dobu 2-3 s)
   c) **zvednout** podpěru hlavy (po dobu 2-3 s)
   d) **snížit** podpěru nohou (po dobu 2-3 s)
   e) **snížit** podpěru hlavy (po dobu 2-3 s)
   f) přerušit oční kontakt s aplikací

7. **Test 2** – opakování **Testu 1** ☑
8. **Test 3** – opakování **Testu 1** ☑

Čas při ukončení testování: 8:30

Poznámky instruktora k experimentu:

PA [illegible] C 19

Otázky pro pacienta

Získal/a jste jistotu v ovládání? *ano / spíše ano / nevím / spíše ne / ne*

Jak jste spokojen/a s ovládáním aplikace?

aktivace ovládání *velmi snadná / snadná / běžná / obtížná / velmi obtížná*

způsob volby polohy *velmi snadný / snadný / běžný / obtížný / velmi obtížný*

informace o nastavení lůžka *velmi snadné / snadné / běžné / obtížné / velmi obtížné*

přehlednost ovládání *velmi přehledné / přehledné / běžné / nepřehledné / velmi nepřehledné*

Upřednostnil/a byste ovládání jiným způsobem? *ano / spíše ano / nevím / spíše ne / ne*

Pokud ano jakým (např. řeč)? ....

Máte nějaký poznatek k testu nebo ovládání?

- display by mohl být dál od očí

Jak byste hodnotil/a Vaši aktuální intenzitu bolesti na uvedené obrazové škále?

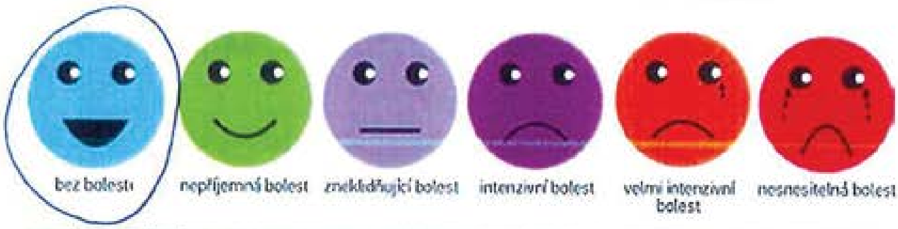

**MALÁ NEBO ŽÁDNÁ BOLEST** Jste schopni se bolesti přizpůsobit	Velmi mírná bolest	☐	Velmi lehká, sotva znatelná bolest
	Nepříjemná bolest	☐	Menší bolest, lze ji přirovnat k lehkému štípání
	Přijatelná bolest	☐	Velmi nápadná bolest podobající se píchnutí injekce
**MÍRNÁ BOLEST** Neslučitelná s mnoha aktivitami	Znekliďňující bolest	☐	Silná, hluboká bolest
	Velmi znekliďňující bolest	☐	Silná, hluboká, pronikavá bolest, např. vyvrknutý kotník
	Intenzivní bolest	☐	Ještě silnější bolest srovnatelná s bodnutím několika včelích žihadel
**TĚŽKÁ BOLEST** Nejste schopni fungovat	Velmi intenzivní bolest	☐	Bolest srovnatelná s průměrnou migrénou
	Naprosto hrozná bolest	☐	Bolest srovnatelná s porodem nebo opravdu silnou migrénou
	Nesnesitelná bolest	☐	Bolest nelze ignorovat, vyžaduje léky proti bolesti, nutné navštívit lékaře

Jak byste posoudil/a Vaši obvyklou míru zrakové pozornosti nebo výdrže při sledování běžného celovečerního filmu?

sleduji bez problémů / sleduji s přestávkami / nemůžu sledovat / nezajímá mne to

Děkujeme Vám za účast v experimentu a při vyplnění dotazníku...

03,0 C20

Informace, dotazník a protokol experimentu pro

Bezkontaktní ovládání polohovacího lůžka

řešitel: Ing. Martin Kopeček, MEng, kopecema@lfhk.cuni.cz

Dotazník vyplňte co možná nejdříve po provedení testu.

Otázky pro instruktora

Experiment proveden dne: 13.5.2020

Jméno instruktora: KOPŘIVA

Jméno pacienta: Pohlaví: M Věk: 55

Číslo logu aplikace:

Typ omezení: Paraparéza, Paraplegie, Kvadruplegie, Pentaplegie, Svalové dystrofie, ALS,

Jiné: ŽÁDNÉ

Popis hybnosti: PLNÁ (CHŮZE)

Brýle / kontaktní čočky: ano / ne (zakroužkováno: ne)

Protokol experimentu:

1. Kalibrace senzoru ☑
2. Vysvětlení principu a umožnění získat zkušenost s ovládáním, alespoň 10 minut ☑
3. Uvedení aplikaci do počátečních podmínek – lůžko v bodě nula ☑

Čas při započetí testování: 17:30

4. **Test 1** ☑
   a) zahájit oční kontakt s aplikací
   b) **zvednout** podpěru nohou (po dobu 2-3 s)
   c) **zvednout** podpěru hlavy (po dobu 2-3 s)
   d) **snížit** podpěru nohou (po dobu 2-3 s)
   e) **snížit** podpěru hlavy (po dobu 2-3 s)
   f) přerušit oční kontakt s aplikací
7. **Test 2** – opakování **Testu 1** ☑
8. **Test 3** – opakování **Testu 1** ☑

Čas při ukončení testování: 18:00

Poznámky instruktora k experimentu:

C20

Otázky pro pacienta

Získal/a jste jistotu v ovládání? *ano / spíše ano / nevím / spíše ne / ne*

Jak jste spokojen/a s ovládáním aplikace?

- aktivace ovládání *velmi snadná / snadná / běžná / obtížná / velmi obtížná*
- způsob volby polohy *velmi snadný / snadný / běžný / obtížný / velmi obtížný*
- informace o nastavení lůžka *velmi snadné / snadné / běžné / obtížné / velmi obtížné*
- přehlednost ovládání *velmi přehledné / přehledné / běžné / nepřehledné / velmi nepřehledné*

Upřednostnil/a byste ovládání jiným způsobem? *ano / spíše ano / nevím / spíše ne / ne*

Pokud ano jakým (např. řeč)? ....

Máte nějaký poznatek k testu nebo ovládání?

Jak byste hodnotil/a Vaši aktuální intenzitu bolesti na uvedené obrazové škále?

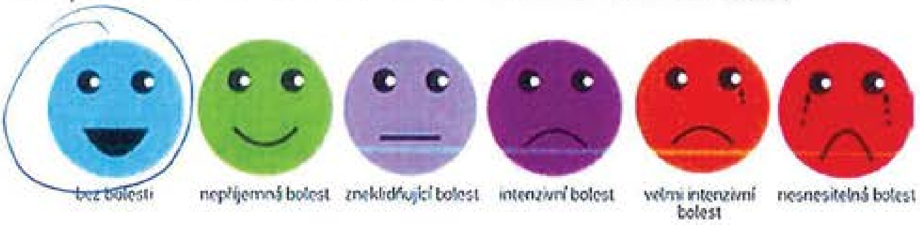

**MALÁ NEBO ŽÁDNÁ BOLEST** Jste schopni se bolesti přizpůsobit	Velmi mírná bolest	☐	Velmi lehká, sotva znatelná bolest
	Nepříjemná bolest	☐	Menší bolest, lze ji přirovnat k lehkému štípání
	Přijatelná bolest	☐	Velmi nápadná bolest podobající se píchnutí injekce
**MÍRNÁ BOLEST** Neslučitelná s mnoha aktivitami	Zneklidňující bolest	☐	Silná, hluboká bolest
	Velmi zneklidňující bolest	☐	Silná, hluboká, pronikavá bolest, např. vyvrknutý kotník
	Intenzivní bolest	☐	Ještě silnější bolest srovnatelná s bodnutím několika včelích žihadel
**TĚŽKÁ BOLEST** Nejste schopni fungovat	Velmi intenzivní bolest	☐	Bolest srovnatelná s průměrnou migrénou
	Naprosto hrozná bolest	☐	Bolest srovnatelná s porodem nebo opravdu silnou migrénou
	Nesnesitelná bolest	☐	Bolest nelze ignorovat, vyžaduje léky proti bolesti, nutné navštívit lékaře

Jak byste posoudil/a Vaši obvyklou míru zrakové pozornosti nebo výdrže při sledování běžného celovečerního filmu?

sleduji bez problémů / sleduji s přestávkami / nemůžu sledovat / nezajímá mne to

Děkujeme Vám za účast v experimentu a při vyplnění dotazníku...

C21

Informace, dotazník a protokol experimentu pro
Bezkontaktní ovládání polohovacího lůžka

řešitel: Ing. Martin Kopeček, MEng, kopecema@lfhk.cuni.cz

Dotazník vyplňte co možná nejdříve po provedení testu.

Otázky pro instruktora

Experiment proveden dne: 12. 5. 2020

Jméno instruktora: KOPEČEK

Jméno pacienta: Pohlaví: M Věk: 45

Číslo logu aplikace:

Typ omezení: Paraparéza, Paraplegie, Kvadruplegie, Pentaplegie, Svalové dystrofie, ALS,

Jiné: žádné

Popis hybnosti: plná

Brýle / kontaktní čočky: (ano) / ne

Protokol experimentu:

1. Kalibrace senzoru ☑
2. Vysvětlení principu a umožnění získat zkušenost s ovládáním, alespoň 10 minut ☑
3. Uvedení aplikaci do počátečních podmínek – lůžko v bodě nula ☑

Čas při započetí testování: 14 15

4. **Test 1** ☑
   a) zahájit oční kontakt s aplikací
   b) **zvednout** podpěru nohou (po dobu 2-3 s)
   c) **zvednout** podpěru hlavy (po dobu 2-3 s)
   d) **snížit** podpěru nohou (po dobu 2-3 s)
   e) **snížit** podpěru hlavy (po dobu 2-3 s)
   f) přerušit oční kontakt s aplikací
7. **Test 2** – opakování **Testu 1** ☑
8. **Test 3** – opakování **Testu 1** ☑

Čas při ukončení testování: 14 30

Poznámky instruktora k experimentu:

C21

Otázky pro pacienta

Získal/a jste jistotu v ovládání? *ano / spíše ano / nevím / spíše ne / ne* (zakroužkováno: spíše ano)

Jak jste spokojen/a s ovládáním aplikace?

- aktivace ovládání *velmi snadná / snadná / běžná / obtížná / velmi obtížná* (zakroužkováno: velmi snadná)
- způsob volby polohy *velmi snadný / snadný / běžný / obtížný / velmi obtížný* (zakroužkováno: běžný)
- informace o nastavení lůžka *velmi snadné / snadné / běžné / obtížné / velmi obtížné*
- přehlednost ovládání *velmi přehledné / přehledné / běžné / nepřehledné / velmi nepřehledné* (zakroužkováno: přehledné)

Upřednostnil/a byste ovládání jiným způsobem? *ano / spíše ano / nevím / spíše ne / ne* (zakroužkováno: spíše ne)

Pokud ano jakým (např. řeč)? ......EEG......

Máte nějaký poznatek k testu nebo ovládání?

PŘEPÍNÁNÍ POLOH (NOHY / HLAVA) SE OBČAS TĚŽKOPÁDNĚ, REAGUJE NA 2.-3. POKUS

Jak byste hodnotil/a Vaši aktuální intenzitu bolesti na uvedené obrazové škále?

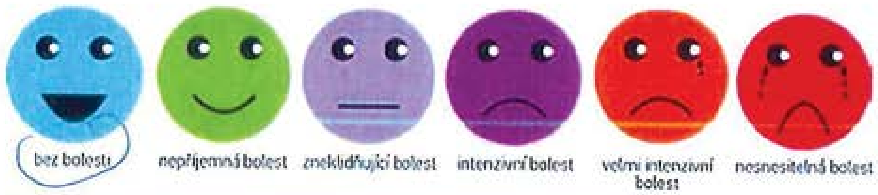

**MALÁ NEBO ŽÁDNÁ BOLEST** Jste schopni se bolesti přizpůsobit	Velmi mírná bolest	☐	Velmi lehká, sotva znatelná bolest
	Nepříjemná bolest	☐	Menší bolest, lze ji přirovnat k lehkému štípání
	Přijatelná bolest	☐	Velmi nápadná bolest podobající se píchnutí injekce
**MÍRNÁ BOLEST** Neslučitelná s mnoha aktivitami	Zneklidňující bolest	☐	Silná, hluboká bolest
	Velmi zneklidňující bolest	☐	Silná, hluboká, pronikavá bolest, např. vyvrknutý kotník
	Intenzivní bolest	☐	Ještě silnější bolest srovnatelná s bodnutím několika včelích žihadel
**TĚŽKÁ BOLEST** Nejste schopni fungovat	Velmi intenzivní bolest	☐	Bolest srovnatelná s průměrnou migrénou
	Naprosto hrozná bolest	☐	Bolest srovnatelná s porodem nebo opravdu silnou migrénou
	Nesnesitelná bolest	☐	Bolest nelze ignorovat, vyžaduje léky proti bolesti, nutné navštívit lékaře

Jak byste posoudil/a Vaši obvyklou míru zrakové pozornosti nebo výdrže při sledování běžného celovečerního filmu?

sleduji bez problémů / sleduji s přestávkami / nemůžu sledovat / nezajímá mne to (zakroužkováno: sleduji bez problémů)

Děkujeme Vám za účast v experimentu a při vyplnění dotazníku...

C22

Informace, dotazník a protokol experimentu pro

Bezkontaktní ovládání polohovacího lůžka

řešitel: Ing. Martin Kopeček, MEng, kopecema@lfhk.cuni.cz

Dotazník vyplňte co možná nejdříve po provedení testu.

Otázky pro instruktora

Experiment proveden dne: 12. 5. 2020

Jméno instruktora: KOPEČEK

Jméno pacienta: Pohlaví: M Věk: 72

Číslo logu aplikace:

Typ omezení: Paraparéza, Paraplegie, Kvadruplegie, Pentaplegie, Svalové dystrofie, ALS,

Jiné: NEMÁ

Popis hybnosti: PLNÁ

Brýle / kontaktní čočky: ano / ne (zakroužkováno: ano)

Protokol experimentu:

1. Kalibrace senzoru ☑
2. Vysvětlení principu a umožnění získat zkušenost s ovládáním, alespoň 10 minut ☑
3. Uvedení aplikaci do počátečních podmínek – lůžko v bodě nula ☑

Čas při započetí testování: 11 15

4. **Test 1** ☑
   a) zahájit oční kontakt s aplikací
   b) **zvednout** podpěru nohou (po dobu 2-3 s)
   c) **zvednout** podpěru hlavy (po dobu 2-3 s)
   d) **snížit** podpěru nohou (po dobu 2-3 s)
   e) **snížit** podpěru hlavy (po dobu 2-3 s)
   f) přerušit oční kontakt s aplikací

7. **Test 2** – opakování **Testu 1** ☑
8. **Test 3** – opakování **Testu 1** ☑

Čas při ukončení testování: 11 45

Poznámky instruktora k experimentu:

C22

Otázky pro pacienta

Získal/a jste jistotu v ovládání? *ano / spíše ano / nevím / spíše ne / ne*

Jak jste spokojen/a s ovládáním aplikace?

- aktivace ovládání — *velmi snadná / snadná / běžná / obtížná / velmi obtížná*
- způsob volby polohy — *velmi snadný / snadný / běžný / obtížný / velmi obtížný*
- informace o nastavení lůžka — *velmi snadné / snadné / běžné / obtížné / velmi obtížné*
- přehlednost ovládání — *velmi přehledné / přehledné / běžné / nepřehledné / velmi nepřehledné*

Upřednostnil/a byste ovládání jiným způsobem? *ano / spíše ano / nevím / spíše ne / ne*

Pokud ano jakým (např. řeč)? ....

Máte nějaký poznatek k testu nebo ovládání?

NE

Jak byste hodnotil/a Vaši aktuální intenzitu bolesti na uvedené obrazové škále?

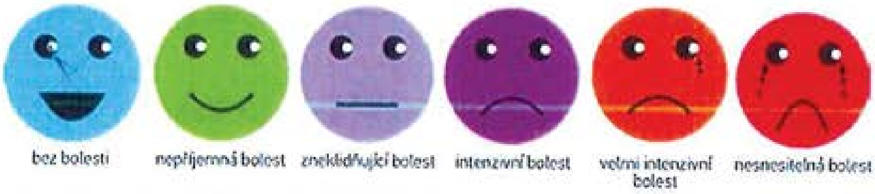

**MALÁ NEBO ŽÁDNÁ BOLEST** Jste schopni se bolesti přizpůsobit	Velmi mírná bolest	☐	Velmi lehká, sotva znatelná bolest
	Nepříjemná bolest	☐	Menší bolest, lze ji přirovnat k lehkému štípání
	Přijatelná bolest	☐	Velmi nápadná bolest podobající se píchnutí injekce
**MÍRNÁ BOLEST** Neslučitelná s mnoha aktivitami	Zneklidňující bolest	☐	Silná, hluboká bolest
	Velmi zneklidňující bolest	☐	Silná, hluboká, pronikavá bolest, např. vyvrknutý kotník
	Intenzivní bolest	☐	Ještě silnější bolest srovnatelná s bodnutím několika včelích žihadel
**TĚŽKÁ BOLEST** Nejste schopni fungovat	Velmi intenzivní bolest	☐	Bolest srovnatelná s průměrnou migrénou
	Naprosto hrozná bolest	☐	Bolest srovnatelná s porodem nebo opravdu silnou migrénou
	Nesnesitelná bolest	☐	Bolest nelze ignorovat, vyžaduje léky proti bolesti, nutné navštívit lékaře

Jak byste posoudil/a Vaši obvyklou míru zrakové pozornosti nebo výdrže při sledování běžného celovečerního filmu?

sleduji bez problémů / sleduji s přestávkami / nemůžu sledovat / nezajímá mne to

Děkujeme Vám za účast v experimentu a při vyplnění dotazníku...

C23

Informace, dotazník a protokol experimentu pro
Bezkontaktní ovládání polohovacího lůžka

řešitel: Ing. Martin Kopeček, MEng, kopecema@lfhk.cuni.cz

Dotazník vyplňte co možná nejdříve po provedení testu.

Otázky pro instruktora

Experiment proveden dne: 11.5.2020

Jméno instruktora: KOPEČEK

Jméno pacienta: Pohlaví: M Věk: 56

Číslo logu aplikace:

Typ omezení: Paraparéza, Paraplegie, Kvadruplegie, Pentaplegie, Svalové dystrofie, ALS,

Jiné: NEMA

Popis hybnosti: PLNÁ

Brýle / kontaktní čočky: (ano) / ne

Protokol experimentu:

1. Kalibrace senzoru ☑
2. Vysvětlení principu a umožnění získat zkušenost s ovládáním, alespoň 10 minut ☑
3. Uvedení aplikaci do počátečních podmínek – lůžko v bodě nula ☑

Čas při započetí testování: 13

4. **Test 1** ☑
    a) zahájit oční kontakt s aplikací
    b) **zvednout** podpěru nohou (po dobu 2-3 s)
    c) **zvednout** podpěru hlavy (po dobu 2-3 s)
    d) **snížit** podpěru nohou (po dobu 2-3 s)
    e) **snížit** podpěru hlavy (po dobu 2-3 s)
    f) přerušit oční kontakt s aplikací
7. **Test 2** – opakování **Testu 1** ☑
8. **Test 3** – opakování **Testu 1** ☑

Čas při ukončení testování: 13³⁰

Poznámky instruktora k experimentu:

C23

Otázky pro pacienta

Získal/a jste jistotu v ovládání? *ano / spíše ano/ nevím / spíše ne / ne*

Jak jste spokojen/a s ovládáním aplikace?

- aktivace ovládání *velmi snadná / snadná / běžná / obtížná / velmi obtížná*
- způsob volby polohy *velmi snadný / snadný / běžný / obtížný / velmi obtížný*
- informace o nastavení lůžka *velmi snadné / snadné / běžné / obtížné / velmi obtížné*
- přehlednost ovládání *velmi přehledné / přehledné/ běžné / nepřehledné/ velmi nepřehledné*

Upřednostnil/a byste ovládání jiným způsobem? *ano / spíše ano/ nevím / spíše ne / ne*

Pokud ano jakým (např. řeč)? ....

Máte nějaký poznatek k testu nebo ovládání? NE

Jak byste hodnotil/a Vaši aktuální intenzitu bolesti na uvedené obrazové škále?

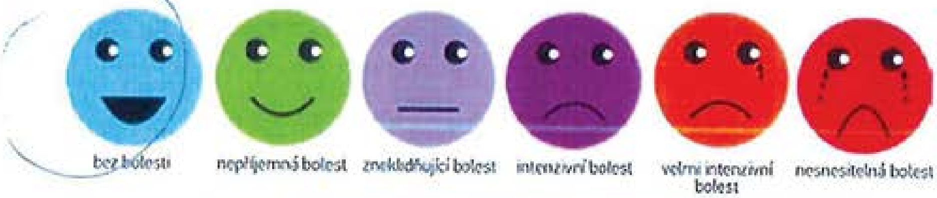

**MALÁ NEBO ŽÁDNÁ BOLEST** Jste schopni se bolesti přizpůsobit	Velmi mírná bolest	☐	Velmi lehká, sotva znatelná bolest
	Nepříjemná bolest	☐	Menší bolest, lze ji přirovnat k lehkému štípání
	Přijatelná bolest	☐	Velmi nápadná bolest podobající se píchnutí injekce
**MÍRNÁ BOLEST** Neslučitelná s mnoha aktivitami	Zneklidňující bolest	☐	Silná, hluboká bolest
	Velmi zneklidňující bolest	☐	Silná, hluboká, pronikavá bolest, např. vyvrknutý kotník
	Intenzivní bolest	☐	Ještě silnější bolest srovnatelná s bodnutím několika včelích žihadel
**TĚŽKÁ BOLEST** Nejste schopni fungovat	Velmi intenzivní bolest	☐	Bolest srovnatelná s průměrnou migrénou
	Naprosto hrozná bolest	☐	Bolest srovnatelná s porodem nebo opravdu silnou migrénou
	Nesnesitelná bolest	☐	Bolest nelze ignorovat, vyžaduje léky proti bolesti, nutné navštívit lékaře

Jak byste posoudil/a Vaši obvyklou míru zrakové pozornosti nebo výdrže při sledování běžného celovečerního filmu?

sleduji bez problémů / sleduji s přestávkami / nemůžu sledovat / nezajímá mne to

Děkujeme Vám za účast v experimentu a při vyplnění dotazníku...

[illegible] C24

Otázky pro pacienta

Získal/a jste jistotu v ovládání? *ano / spíše ano/ nevím / spíše ne / ne*

Jak jste spokojen/a s ovládáním aplikace?

- aktivace ovládání — *velmi snadná / snadná / běžná / obtížná / velmi obtížná*
- způsob volby polohy — *velmi snadný / snadný / běžný / obtížný / velmi obtížný*
- informace o nastavení lůžka ? — *velmi snadné / snadné / běžné / obtížné / velmi obtížné*
- přehlednost ovládání — *velmi přehledné / přehledné/ běžné / nepřehledné/ velmi nepřehledné*

Upřednostnil/a byste ovládání jiným způsobem? *ano / spíše ano/ nevím / spíše ne / ne*

Pokud ano jakým (např. řeč)? ....

Máte nějaký poznatek k testu nebo ovládání?

Vylepšit kontrast / informace o tom, kde se nacházím a i co dělám v průběhu potvrzování.

Jak byste hodnotil/a Vaši aktuální intenzitu bolesti na uvedené obrazové škále?

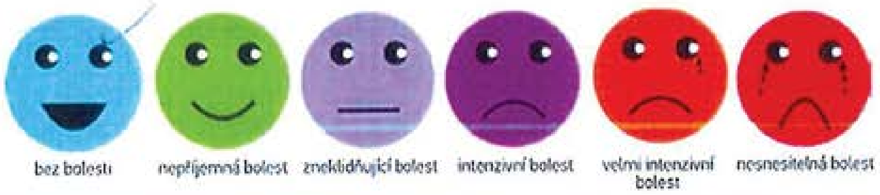

**MALÁ NEBO ŽÁDNÁ BOLEST** Jste schopni se bolesti přizpůsobit	Velmi mírná bolest	☐	Velmi lehká, sotva znatelná bolest
	Nepříjemná bolest	☐	Menší bolest, lze ji přirovnat k lehkému štípání
	Přijatelná bolest	☐	Velmi nápadná bolest podobající se píchnutí injekce
**MÍRNÁ BOLEST** Neslučitelná s mnoha aktivitami	Zneklidňující bolest	☐	Silná, hluboká bolest
	Velmi zneklidňující bolest	☐	Silná, hluboká, pronikavá bolest, např. vyvrknutý kotník
	Intenzivní bolest	☐	Ještě silnější bolest srovnatelná s bodnutím několika včelích žihadel
**TĚŽKÁ BOLEST** Nejste schopni fungovat	Velmi intenzivní bolest	☐	Bolest srovnatelná s průměrnou migrénou
	Naprosto hrozná bolest	☐	Bolest srovnatelná s porodem nebo opravdu silnou migrénou
	Nesnesitelná bolest	☐	Bolest nelze ignorovat, vyžaduje léky proti bolesti, nutné navštívit lékaře

Jak byste posoudil/a Vaši obvyklou míru zrakové pozornosti nebo výdrže při sledování běžného celovečerního filmu?

sleduji bez problémů / sleduji s přestávkami / nemůžu sledovat / nezajímá mne to

Děkujeme Vám za účast v experimentu a při vyplnění dotazníku...

#!?

C24

Informace, dotazník a protokol experimentu pro

Bezkontaktní ovládání polohovacího lůžka

řešitel: Ing. Martin Kopeček, MEng, kopecema@lfhk.cuni.cz

Dotazník vyplňte co možná nejdříve po provedení testu.

Otázky pro instruktora

Experiment proveden dne: 5. 5. 2020

Jméno instruktora: KOPEČEK M.

Jméno pacienta: Pohlaví: M Věk: 41

Číslo logu aplikace:

Typ omezení: Paraparéza, Paraplegie, Kvadruplegie, Pentaplegie, Svalové dystrofie, ALS,

Jiné: ŽÁDNÉ

Popis hybnosti: PLNÁ

Brýle / kontaktní čočky: ano / ne (zakroužkováno: ne)

Protokol experimentu:

1. Kalibrace senzoru ☑
2. Vysvětlení principu a umožnění získat zkušenost s ovládáním, alespoň 10 minut ☑
3. Uvedení aplikaci do počátečních podmínek – lůžko v bodě nula ☑

Čas při započetí testování: 12:15

4. **Test 1** ☑
   a) zahájit oční kontakt s aplikací
   b) **zvednout** podpěru nohou (po dobu 2-3 s)
   c) **zvednout** podpěru hlavy (po dobu 2-3 s)
   d) **snížit** podpěru nohou (po dobu 2-3 s)
   e) **snížit** podpěru hlavy (po dobu 2-3 s)
   f) přerušit oční kontakt s aplikací
7. **Test 2** – opakování **Testu 1** ☑
8. **Test 3** – opakování **Testu 1** ☑

Čas při ukončení testování: 12:30

Poznámky instruktora k experimentu:

C25

Informace, dotazník a protokol experimentu pro

Bezkontaktní ovládání polohovacího lůžka

řešitel: Ing. Martin Kopeček, MEng, kopecema@lfhk.cuni.cz

Dotazník vyplňte co možná nejdříve po provedení testu.

Otázky pro instruktora

Experiment proveden dne: 5. 5. 2020

Jméno instruktora: KOPEČEK M.

Jméno pacienta: M 52

Číslo logu aplikace: 2,1,2

Typ omezení: Paraparéza, Paraplegie, Kvadruplegie, Pentaplegie, Svalové dystrofie, ALS,

jiné: ŽÁDNÉ

Popis hybnosti: PLNÁ

Brýle / kontaktní čočky: ano / ne (zakroužkováno: ne)

Protokol experimentu:

1. Kalibrace senzoru ☑
2. Vysvětlení principu a umožnění získat zkušenost s ovládáním, alespoň 10 minut ☑
3. Uvedení aplikaci do počátečních podmínek – lůžko v bodě nula ☑

Čas při započetí testování: 9⁰⁰

4. **Test 1** ☑
   a) zahájit oční kontakt s aplikací
   b) **zvednout** podpěru nohou (po dobu 2-3 s)
   c) **zvednout** podpěru hlavy (po dobu 2-3 s)
   d) **snížit** podpěru nohou (po dobu 2-3 s)
   e) **snížit** podpěru hlavy (po dobu 2-3 s)
   f) přerušit oční kontakt s aplikací

7. **Test 2** – opakování **Testu 1** ☑

8. **Test 3** – opakování **Testu 1** ☑

Čas při ukončení testování: 11⁰⁰

Poznámky instruktora k experimentu:

C25

Otázky pro pacienta

Získal/a jste jistotu v ovládání? *ano / spíše ano / nevím / spíše ne / ne*

Jak jste spokojen/a s ovládáním aplikace?

- aktivace ovládání — *velmi snadná / snadná / běžná / obtížná / velmi obtížná*
- způsob volby polohy — *velmi snadný / snadný / běžný / obtížný / velmi obtížný*
- informace o nastavení lůžka — *velmi snadné / snadné / běžné / obtížné / velmi obtížné*
- přehlednost ovládání — *velmi přehledné / přehledné / běžné / nepřehledné/ velmi nepřehledné*

Upřednostnil/a byste ovládání jiným způsobem? *ano / spíše ano / nevím / spíše ne / ne*

Pokud ano jakým (např. řeč)? ....

Máte nějaký poznatek k testu nebo ovládání? Pomohl by mi kurzor, který by byl v místě pohledu.

Jak byste hodnotil/a Vaši aktuální intenzitu bolesti na uvedené obrazové škále?

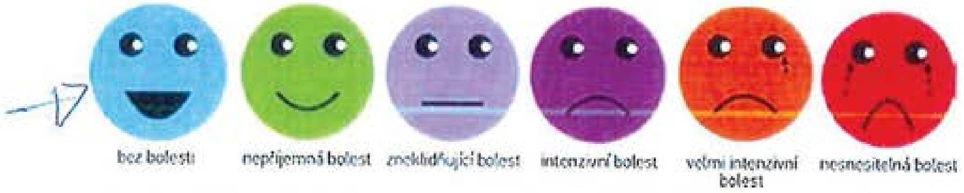

**MALÁ NEBO ŽÁDNÁ BOLEST** Jste schopni se bolesti přizpůsobit	Velmi mírná bolest	☐	Velmi lehká, sotva znatelná bolest
	Nepříjemná bolest	☐	Menší bolest, lze ji přirovnat k lehkému štípání
	Přijatelná bolest	☐	Velmi nápadná bolest podobající se píchnutí injekce
**MÍRNÁ BOLEST** Neslučitelná s mnoha aktivitami	Zneklidňující bolest	☐	Silná, hluboká bolest
	Velmi zneklidňující bolest	☐	Silná, hluboká, pronikavá bolest, např. vyvrknutý kotník
	Intenzivní bolest	☐	Ještě silnější bolest srovnatelná s bodnutím několika včelích žihadel
**TĚŽKÁ BOLEST** Nejste schopni fungovat	Velmi intenzivní bolest	☐	Bolest srovnatelná s průměrnou migrénou
	Naprosto hrozná bolest	☐	Bolest srovnatelná s porodem nebo opravdu silnou migrénou
	Nesnesitelná bolest	☐	Bolest nelze ignorovat, vyžaduje léky proti bolesti, nutné navštívit lékaře

Jak byste posoudil/a Vaši obvyklou míru zrakové pozornosti nebo výdrže při sledování běžného celovečerního filmu?

sleduji bez problémů / sleduji s přestávkami / nemůžu sledovat / nezajímá mne to

Děkujeme Vám za účast v experimentu a při vyplnění dotazníku... ..prosím :)

[illegible] C26

Informace, dotazník a protokol experimentu pro

Bezkontaktní ovládání polohovacího lůžka

řešitel: Ing. Martin Kopeček, MEng, kopecema@lfhk.cuni.cz

Dotazník vyplňte co možná nejdříve po provedení testu.

Otázky pro instruktora

Experiment proveden dne:

Jméno instruktora:

Jméno pacienta: Pohlaví: ž Věk: 59

Číslo logu aplikace: 1,2,3

Typ omezení: Paraparéza, Paraplegie, Kvadruplegie, Pentaplegie, Svalové dystrofie, ALS, —

Jiné: —

Popis hybnosti: PLNÁ

Brýle / kontaktní čočky: ano / (ne)

Protokol experimentu:

1. Kalibrace senzoru ☑
2. Vysvětlení principu a umožnění získat zkušenost s ovládáním, alespoň 10 minut ☑
3. Uvedení aplikaci do počátečních podmínek – lůžko v bodě nula ☑

Čas při započetí testování: 11:45

4. **Test 1** ☑
   a) zahájit oční kontakt s aplikací
   b) **zvednout** podpěru nohou (po dobu 2-3 s)
   c) **zvednout** podpěru hlavy (po dobu 2-3 s)
   d) **snížit** podpěru nohou (po dobu 2-3 s)
   e) **snížit** podpěru hlavy (po dobu 2-3 s)
   f) přerušit oční kontakt s aplikací
7. **Test 2** – opakování **Testu 1** ☑
8. **Test 3** – opakování **Testu 1** ☑

Čas při ukončení testování: 11:53

Poznámky instruktora k experimentu: OK

C26

Otázky pro pacienta

Získal/a jste jistotu v ovládání? *ano / spíše ano/ nevím / spíše ne / ne*

Jak jste spokojen/a s ovládáním aplikace?

- aktivace ovládání — *velmi snadná / snadná / běžná / obtížná / velmi obtížná*
- způsob volby polohy — *velmi snadný / snadný / běžný / obtížný / velmi obtížný*
- informace o nastavení lůžka — *velmi snadné / snadné / běžné / obtížné / velmi obtížné*
- přehlednost ovládání — *velmi přehledné / přehledné/ běžné / nepřehledné/ velmi nepřehledné*

Upřednostnil/a byste ovládání jiným způsobem? *ano / spíše ano/ nevím / spíše ne / ne*

Pokud ano jakým (např. řeč)? ....

Máte nějaký poznatek k testu nebo ovládání?

Jak byste hodnotil/a Vaši aktuální intenzitu bolesti na uvedené obrazové škále?

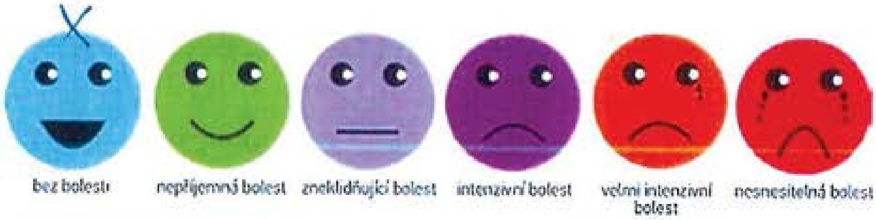

**MALÁ NEBO ŽÁDNÁ BOLEST** Jste schopni se bolesti přizpůsobit	Velmi mírná bolest	☐	Velmi lehká, sotva znatelná bolest
	Nepříjemná bolest	☐	Menší bolest, lze ji přirovnat k lehkému štípání
	Přijatelná bolest	☐	Velmi nápadná bolest podobající se píchnutí injekce
**MÍRNÁ BOLEST** Neslučitelná s mnoha aktivitami	Zneklidňující bolest	☐	Silná, hluboká bolest
	Velmi zneklidňující bolest	☐	Silná, hluboká, pronikavá bolest, např. vyvrknutý kotník
	Intenzivní bolest	☐	Ještě silnější bolest srovnatelná s bodnutím několika včelích žihadel
**TĚŽKÁ BOLEST** Nejste schopni fungovat	Velmi intenzivní bolest	☐	Bolest srovnatelná s průměrnou migrénou
	Naprosto hrozná bolest	☐	Bolest srovnatelná s porodem nebo opravdu silnou migrénou
	Nesnesitelná bolest	☐	Bolest nelze ignorovat, vyžaduje léky proti bolesti, nutné navštívit lékaře

Jak byste posoudil/a Vaši obvyklou míru zrakové pozornosti nebo výdrže při sledování běžného celovečerního filmu?

sleduji bez problémů / sleduji s přestávkami / nemůžu sledovat / nezajímá mne to

Děkujeme Vám za účast v experimentu a při vyplnění dotazníku...
